# Temporal and Cell‐Specific Regulation of Synaptic Homeostasis by the Chromatin Remodeler *Chd1*

**DOI:** 10.1002/advs.202510538

**Published:** 2026-03-15

**Authors:** Danielle T. Morency, Tao Cui, Yimei Cai, Chloe Lok, Rachel E. Nokku, Ruoxian Huang, Grace L. Chu, Yumeng Xie, Saleem W. Abu‐Tayeh, Kaikai He, Chengjie Qiu, Junyi Wang, Paxton M. Paganelli, Ting Wang, Gabrielle Williams, Sreejith Nair, Huadong Pei, Dion K. Dickman, Stefano Vicini, Tingting Wang

**Affiliations:** ^1^ Department of Pharmacology & Physiology Georgetown University Medical Center Washington, D.C. USA; ^2^ Interdisciplinary Program in Neuroscience Georgetown University Medical Center Washington, D.C. USA; ^3^ Biology Department Georgetown University Washington, D.C. USA; ^4^ Department of Human Science School of Health Georgetown University Washington, D.C. USA; ^5^ Department of Neurobiology University of Southern California Los Angeles CA USA; ^6^ Department of Oncology Georgetown Lombardi Comprehensive Cancer Center Georgetown University Medical Center Washington, D.C. USA

**Keywords:** autism, Chd1, epigenetic regulation, epilepsy, glia, neuromuscular junction, presynaptic homeostatic plasticity

## Abstract

Disruptions in chromatin remodelers and synaptic proteins represent major genetic risk factors for autism spectrum disorder (ASD), yet how these distinct gene classes converge to impair circuit function remains unclear. *CHD2*, a chromatin remodeler linked to ASD, epilepsy, and intellectual disability, regulates gene expression through epigenetic mechanisms. In *Drosophila*, its homologue *Chd1* functions as a key regulator of presynaptic homeostatic potentiation (PHP), a conserved form of synaptic plasticity that stabilizes neurotransmission. Electrophysiology, calcium imaging, super‐resolution microscopy, behavioral assays, and machine learning‐based analysis reveal that *Chd1* acts in a temporal and cell type‐specific manner: it is required in perineurial glia for rapid PHP induction and in motoneurons, muscle, and glia for long‐term maintenance. *Chd1* controls presynaptic calcium influx and expansion of the readily releasable vesicle pool, both core features of homeostatic compensation. An electrophysiology‐based genetic screen guided by unsupervised machine learning identifies 14 *Chd1*‐dependent genes necessary for acute PHP, including the glial‐specific effector *Cadherin 74A*. Loss of *Chd1* increases seizure susceptibility and disrupts motor function, mirroring phenotypes observed in *CHD2*‐related neurodevelopmental disorders. These findings establish a mechanistic connection between chromatin remodeling and synaptic homeostasis and identify glial epigenetic regulation as a critical modulator of circuit stability in health and disease.

## Graphical abstract

*Chd1*, the *Drosophila* homologue of mammalian *CHD2* ‐ a gene linked to autism, epilepsy, and intellectual disability, is required for synaptic homeostatic plasticity. *Chd1* in glia is necessary for the rapid induction of synaptic homeostasis, whereas *Chd1* in motoneurons, muscle, and glia is critical for long‐term maintenance. We identified *Chd1*‐dependent downstream signaling pathways that regulate synaptic homeostasis through a genetic screen.

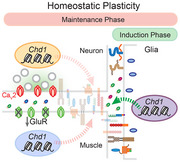

## 1 Introduction

Autism Spectrum Disorder (ASD) is a common neurodevelopmental condition frequently accompanied by epilepsy and intellectual disability [1, 2, 3]. The convergence of symptoms across these disorders suggests shared underlying mechanisms [1, 4]. Among the most prominent genetic risk factors for ASD are chromatin remodelers and synaptic proteins. Notably, members of the Chromodomain Helicase DNA‐binding (CHD) family, such as *CHD2* and *CHD8*, have emerged as recurrent ASD risk genes [5, 6, 7, 8, 9]. Although chromatin remodeling is known to regulate neurodevelopment and memory formation, how disruption of this process affects specific brain cell types and how this contributes to circuit dysfunction remains poorly understood [10, 11, 12, 13]. Recent studies have highlighted glial cells as critical regulators of synaptic development, transmission, and plasticity. Glia not only express ASD‐associated genes but also influence neuronal function via non‐cell‐autonomous mechanisms [14, 15, 16, 17, 18, 19]. Yet, the interplay between neuronal and glial epigenetic regulation at synapses remains largely unexplored.

Synapses are dynamic structures essential for information processing, learning, and behavior. To maintain stability in the face of ongoing plasticity, the nervous system relies on homeostatic mechanisms that buffer synaptic activity within physiological bounds [20, 21, 22]. Disruption of these stabilizing processes contributes to a range of neurological conditions, including ASD, epilepsy, schizophrenia, and neurodegeneration [23, 24, 25, 26, 27, 28]. While synaptic proteins represent a major class of ASD risk factors [5, 7, 29], the connection between chromatin remodelers and synaptic homeostatic plasticity, and their broader implications for ASD, epilepsy, and intellectual disability, is unclear.

Presynaptic homeostatic potentiation (PHP), a form of plasticity that compensates for weakened postsynaptic receptor function by increasing presynaptic neurotransmitter release, is a conserved mechanism from *Drosophila* to humans [20, 28, 30, 31]. When postsynaptic glutamate receptor function is compromised, PHP is induced through an increase in presynaptic calcium influx and the readily releasable vesicle pool (RRP), leading to enhanced neurotransmitter release [32, 33, 34, 35]. This compensatory response restores postsynaptic excitation to baseline levels despite receptor deficits. PHP plays a critical role in stabilizing synaptic function across both the central and peripheral nervous systems [35, 36, 37, 38]. However, dissecting the contributions of chromatin remodelers remains challenging due to the complexity of their downstream gene networks. The *Drosophila* neuromuscular junction (NMJ), a glutamatergic synapse exhibiting robust homeostatic plasticity, serves as a powerful model for electrophysiology‐based genetic screens aimed at identifying genes required for PHP [25, 37, 39, 40]. Recent studies demonstrated that epigenetic regulation of gene expression in peripheral glia is essential for PHP at the *Drosophila* NMJ [39, 41, 42, 43]. These findings revealed that dysfunction of glial epigenetic regulators impairs synaptic homeostatic plasticity, prompting further investigations into the cell type‐specific roles of chromatin remodelers in maintaining synaptic stability.

Among chromatin remodelers, *CHD2* is of particular interest. It encodes an ATP‐dependent enzyme that facilitates chromatin accessibility and histone H3.3 incorporation, regulating gene expression during development and differentiation [44, 45, 46, 47, 48]. *CHD2* insufficiency results in repressive chromatin states and downregulation of key developmental genes  [44, 45, 46]. Notably, *CHD2* regulates transcriptional programs critical for neurodevelopment  [49, 50, 51], including genes encoding synaptic proteins and signaling molecules [51, 52]. *De novo CHD2* mutations have been associated with ASD, epileptic encephalopathy, intellectual disability, and motor dysfunction [5, 6, 53, 54, 55, 56], highlighting its essential role in the nervous system. While studies in mice and human embryonic stem cells indicate a role for *CHD2* in interneuron development [49, 50, 51, 52], its cell type‐specific functions and contributions to synaptic plasticity in the nervous system remain uncharacterized.

Here, we demonstrate that loss‐of‐function mutations in *Drosophila Chd1* increase seizure susceptibility and impair motor function, phenotypes mirroring clinical symptoms observed in patients with *CHD2* mutations [5, 55, 56], suggesting conserved behavioral roles. To investigate the cell type‐specific function of *Chd1* in regulating homeostatic plasticity at the NMJ, we employed electrophysiology, calcium imaging, super‐resolution microscopy, and computational approaches. Our findings reveal that *Chd1* is required in perineurial glia for the rapid induction of PHP, and in motoneurons, muscle, and glia for its long‐term maintenance. Mechanistically, *Chd1* loss disrupts the compensatory increase in presynaptic calcium influx and expansion of the readily releasable vesicle pool, both hallmarks of PHP. Through an electrophysiology‐based genetic screen guided by machine learning, we identified *Cadherin 74A* (*Cad74A*) as one of the glial‐specific targets of *Chd1* that is required for the rapid induction of PHP. Like *Chd1* mutants, *Cad74A* mutants also exhibit severe motor impairments, highlighting the importance of glial epigenetic regulation in maintaining synaptic function. These findings establish *Chd1* as a key integrator of epigenetic control and synaptic homeostasis, revealing how cell type‐specific chromatin remodeling mechanisms contribute to neural circuit stability and offering new insights into the molecular basis of ASD and related disorders.

## 2 Results

### 2.1 *Chd1* Mutants Display Increased Seizure Susceptibility and Impaired Motor Function

**FIGURE 1:**
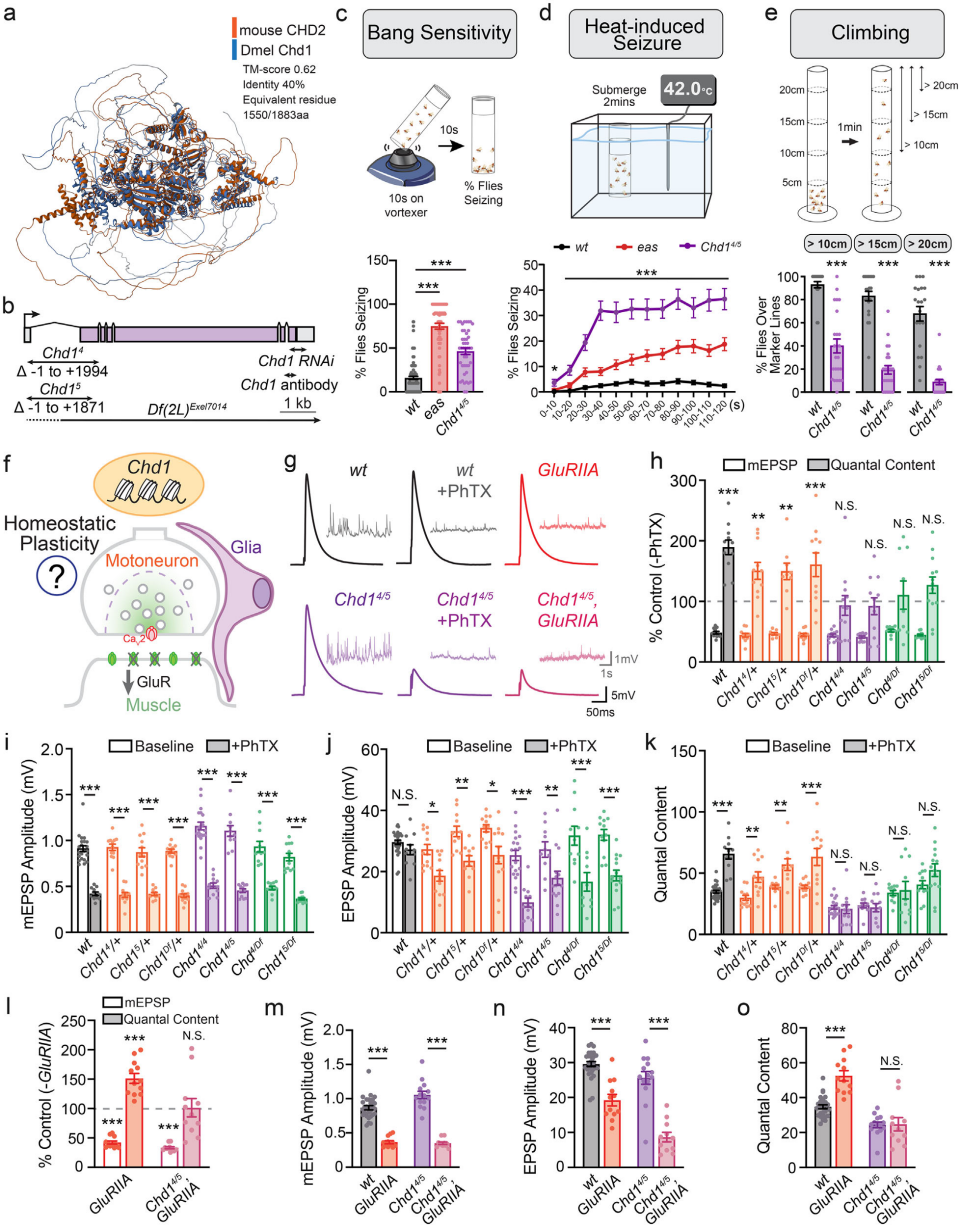
*Chd1* mutants display deficits in behavior and homeostatic plasticity. (a) Alignment of mouse CHD2 (orange) and *Drosophila* Chd1 (blue) protein structures predicted by AlphaFold. Sequence similarities are indicated. (b) Diagram of the *Drosophila Chd1* gene showing the deletions and RNAi target used in this study to disrupt *Chd1* expression, as well as the epitope for the C‐terminal antibody generated against Chd1. (c–e) *Chd1* mutants exhibit increased seizure susceptibility and impaired motor function. (c) Percentage of *wild‐type* (*wt*, *n* = 99 trials), *eas* mutants (*n* = 73), and *Chd1^4/5^* mutants (*n* = 45) exhibiting seizure activity in the bang sensitivity assay. Mean ± SEM; ***p < 0.001; Kruskal–Wallis with Dunn's test for multiple comparisons. (d) Percentage of *wild‐type* (*wt*, *n* = 110), *eas* mutants (*n* = 76), and *Chd1^4/5^* mutants (*n* = 51) exhibiting seizure activity in the heat‐induced seizure assay. Mean ± SEM; *p < 0.05, ***p < 0.001; two‐way ANOVA with Bonferroni test for multiple comparisons. *p*‐values shown for *wild‐type vs. Chd1^4/5^* comparison. (e) Percentage of *wild‐type* (*wt*, *n* = 20) and *Chd1^4/5^* mutants (*n* = 24) able to climb past markers at different distances. Mean ± SEM; ***p < 0.001; unpaired Student's *t*‐test. (f) Schematic of presynaptic homeostatic potentiation (PHP) at the *Drosophila* neuromuscular junction (NMJ). Pharmacological or genetic inhibition of postsynaptic glutamate receptors triggers a compensatory increase in presynaptic neurotransmitter release, which offsets the reduction in mEPSP amplitude and restores the EPSP to baseline. Glial epigenetic signaling is required for PHP. The role of *Chd1* in PHP remains to be elucidated. (g) Representative mEPSP and EPSP traces from *wild‐type* (*wt*) and *Chd1^4/5^* mutants in the absence and presence (+PhTX) of philanthotoxin. *GluRIIA* mutants and *Chd1^4/5^,GluRIIA* double mutants are shown at far right. (h) Average mEPSP amplitude (open bars) and presynaptic release (quantal content, filled bars), expressed as percent change with PhTX relative to baseline (‐PhTX). Genotypes and sample sizes: *wild‐type* (*wt*, *n* = 22, 11 for ‐PhTX and +PhTX, respectively), *Chd1^4^/+* (*n* = 12, 12), *Chd1^5^/+* (*n* = 12, 10), *Chd1^DfExel7014^/+* (*n* = 12, 12), *Chd1^4/4^* (*n* = 19, 12), *Chd1^4/5^* (*n* = 11, 12), *Chd1^4/Df^* (*n* = 11, 10), *Chd1^5/Df^* (*n* = 12, 14). Mean ± SEM; **p < 0.01, ***p < 0.001, N.S. not significant; one‐way ANOVA with Bonferroni test for multiple comparisons. Non‐normalized raw data were used for statistical analysis. (i–k) Non‐normalized values corresponding to (h). Average mEPSP amplitude (i), EPSP amplitude (j), and quantal content (k) in the absence (open bars) and presence (filled bars) of PhTX. Mean ± SEM; *p < 0.05, **p < 0.01, ***p < 0.001, N.S. not significant; one‐way ANOVA with Bonferroni test for multiple comparisons. (l) Average mEPSP amplitude (open bars) and presynaptic release (quantal content, filled bars), expressed as percent change in *GluRIIA* mutants compared to the same genotype without the *GluRIIA* mutation. Genotypes and sample sizes: *wild‐type* (*wt*, *n* = 28), *GluRIIA* (*n* = 12), *Chd1^4/5^* (*n* = 14), *Chd1^4/5^,GluRIIA* (*n* = 11). Mean ± SEM; ***p < 0.001, N.S. not significant; one‐way ANOVA with Bonferroni test for multiple comparisons. (m–o) Non‐normalized values corresponding to (l). Average mEPSP amplitude (m), EPSP amplitude (n), and quantal content (o) in the absence (open bars) and presence (filled bars) of the *GluRIIA* mutation. Mean ± SEM; ***p < 0.001, N.S. not significant; one‐way ANOVA with Bonferroni test for multiple comparisons.

While *Chd1* has been identified as the *Drosophila* homologue of mammalian *CHD2*  [57, 58, 59, 60], structural conservation between the two proteins had not been previously assessed. To address this, we compared the protein sequences and AlphaFold‐predicted structures of *Drosophila* Chd1 and mouse CHD2 [61, 62, 63]. *Drosophila* Chd1 shares 40% sequence identity with mouse CHD2, with 1550 aligned residues out of 1883 total amino acids. Structural alignment yielded a template modeling (TM) score of 0.62, which exceeds the threshold of 0.5 commonly used to indicate proteins with similar folds [63]. These findings indicate substantial structural similarity and evolutionary conservation between *Drosophila* Chd1 and mouse CHD2, supporting the use of *Drosophila* as a model for studying *CHD2* function (Figure 1a). Although *Chd1* is known to regulate gene expression, productivity, stress responses, and lifespan [60, 64, 65], its role in neuronal stabilization in *Drosophila* remains uncharacterized. To investigate this, we analyzed two *Chd1* mutant alleles, which delete the gene's first exon (*Chd1^4^* and *Chd1^5^* [59], Figure  1b). To control for background genetic variation, these mutants were analyzed in trans‐allelic combination with a deficiency line (*Df(3R)Exel7014* indicated as *Chd1^Df^*) [59, 60]. Additionally, we generated a Chd1‐specific antibody targeting the protein's C‐terminal tail (Figure 1b, see Methods for details).

Next, we asked whether *Drosophila Chd1* loss‐of‐function mutants recapitulate the epilepsy‐related and motor function phenotypes observed in human patients with *CHD2* haploinsufficiency [55, 56]. We assessed seizure susceptibility in adult flies using mechanical stimulation (bang sensitivity [66, 67], Figure 1c) and heat‐induced seizure assays  [68] (Figure  1d), and evaluated motor performance using the negative geotaxis (climbing) assay [69] (Figure 1e). As expected, homozygous mutations in *easily shocked* (*eas*), a well‐established epilepsy gene [70, 71], led to a significant increase in seizure incidence in response to both mechanical and heat stimuli, validating the sensitivity of these assays (Figure  1c,d). Notably, *Chd1* trans‐allelic homozygous mutants (*Chd1^4/5^*) also exhibited a marked increase in seizure susceptibility under both conditions compared to *wild‐type* controls (Figure  1c,d). In addition, *Chd1* mutants showed significantly impaired climbing ability, indicative of motor dysfunction (Figure 1e). These phenotypes closely mirror the clinical features of *CHD2* mutation carriers, including increased seizure susceptibility and motor deficits [55, 56]. Together, these results support a conserved role for *Chd1* in regulating neural excitability and motor function in *Drosophila*.

### 2.2 *Chd1* Is Required for the Rapid Induction and Long‐Term Maintenance of PHP

PHP is a highly conserved mechanism that stabilizes synaptic function in response to perturbations. Disruptions in PHP have been associated with imbalances in neuronal excitation and inhibition and are implicated in epilepsy, ASD, and neurodegenerative diseases [28, 39, 42, 72, 73]. To determine whether *Chd1* is required for homeostatic plasticity, we first examined its role in the rapid induction phase of PHP [32, 74] (Figure 1f–k). We applied a pharmacological antagonist Philanthotoxin‐433 (PhTX, 20 µm), of glutamate receptor, for 10 min, and performed electrophysiological recordings at the *Drosophila* third instar larval NMJ. PhTX treatment reduced miniature excitatory postsynaptic potential (mEPSP) amplitude by approximately 50% across all genotypes (Figure 1g–i). In *wild‐type* controls, this postsynaptic perturbation triggered a rapid and robust increase in presynaptic neurotransmitter release (quantal content), effectively restoring excitatory postsynaptic potential (EPSP) amplitude to baseline levels within 10 min (Figure 1g,h,j,k).

When PHP was induced with PhTX in *Chd1* heterozygous mutants (*Chd1^4^/+*, *Chd1^5^/+*, and *Chd1^Df^/+*), we observed a significant increase in quantal content across all genotypes, although EPSP amplitude was not fully restored to baseline levels (Figure 1h,j,k). In contrast, *Chd1* homozygous mutants (*Chd1^4/4^*, *Chd1^4/5^*, *Chd1^4/Df^*, and *Chd1^5/Df^*) showed no increase in quantal content following acute PhTX application, and EPSP amplitude was markedly reduced compared to baseline, indicating a complete loss of PHP (Figure 1g–k). These results demonstrate that *Chd1* is essential for the rapid induction of PHP elicited by PhTX. Baseline EPSP amplitude remains unchanged in both heterozygous and homozygous *Chd1* mutants compared to *wild‐type* controls (Figure S1b). We observed a modest increase in mEPSP amplitude and a corresponding decrease in quantal content in *Chd1^4/4^* and *Chd1^4/5^* homozygotes at baseline (Figure S1a,c). Notably, however, these baseline changes were absent in the trans‐allelic deficiency homozygous mutants (*Chd1^4/Df^* and *Chd1^5/Df^*), suggesting that the mild quantal content deficit in *Chd1^4/4^* and *Chd1^4/5^* may be due to background genetic effects. Given *Chd1^4/Df^* and *Chd1^5/Df^* mutants exhibit normal baseline synaptic transmission, we conclude that *Chd1* is specifically required for the rapid induction of PHP, and that the PHP deficit is not caused by underlying defects in basal synaptic transmission. While *Chd^4/Df^* and *Chd^5/Df^* do not exhibit baseline impairments in synaptic transmission (Figure S1), the large deletion carried by the deficiency line could nonetheless produce enhanced or confounding phenotypes [59]. Therefore, we used the trans‐allelic *Chd1^4/5^* mutants for all complementary electrophysiology, imaging, and behavioral experiments throughout the study.

Next, we assessed whether *Chd1* is required for the long‐term maintenance of PHP using a genetic deletion of the postsynaptic glutamate receptor subunit *GluRIIA*, which is specifically expressed in muscle (*GluRIIA^SP16^*, [75]). Because *GluRIIA* is genetically deleted, the resulting postsynaptic perturbation persists throughout the animal's lifespan, making it a widely used model for studying the long‐term maintenance phase of PHP [37, 74]. As expected, *GluRIIA* mutants exhibited a significant reduction in mEPSP amplitude, similar to the effect observed with PhTX application (Figure 1l,m). This decrease was accompanied by a robust increase in quantal content, effectively driving EPSP amplitude toward *wild‐type* levels and indicating a compensatory enhancement in presynaptic neurotransmitter release (Figure 1l–o). In contrast, *Chd1^4/5^,GluRIIA* double homozygous mutants failed to express chronic PHP, as shown by the absence of a significant increase in quantal content relative to *Chd1^4/5^* single mutants (Figure 1l–o). The EPSP amplitude in *GluRIIA* mutants alone remained slightly below *wild‐type* levels, consistent with incomplete compensation. This could be due to the large upstream deletion in the *GluRIIA^sp16^* allele or differences in genetic background. Nonetheless, the additional reduction in EPSP amplitude observed in *Chd1^4/5^,GluRIIA* double mutants relative to *Chd1^4/5^* single mutants indicates a severe PHP deficit (Figure 1n). Together, these results demonstrate that *Chd1* is essential for both the rapid induction and the long‐term maintenance of PHP in *Drosophila*.

### 2.3 *Chd1* Is Expressed in Motoneurons, Muscle, and Perineurial Glia in *Drosophila*

**FIGURE 2:**
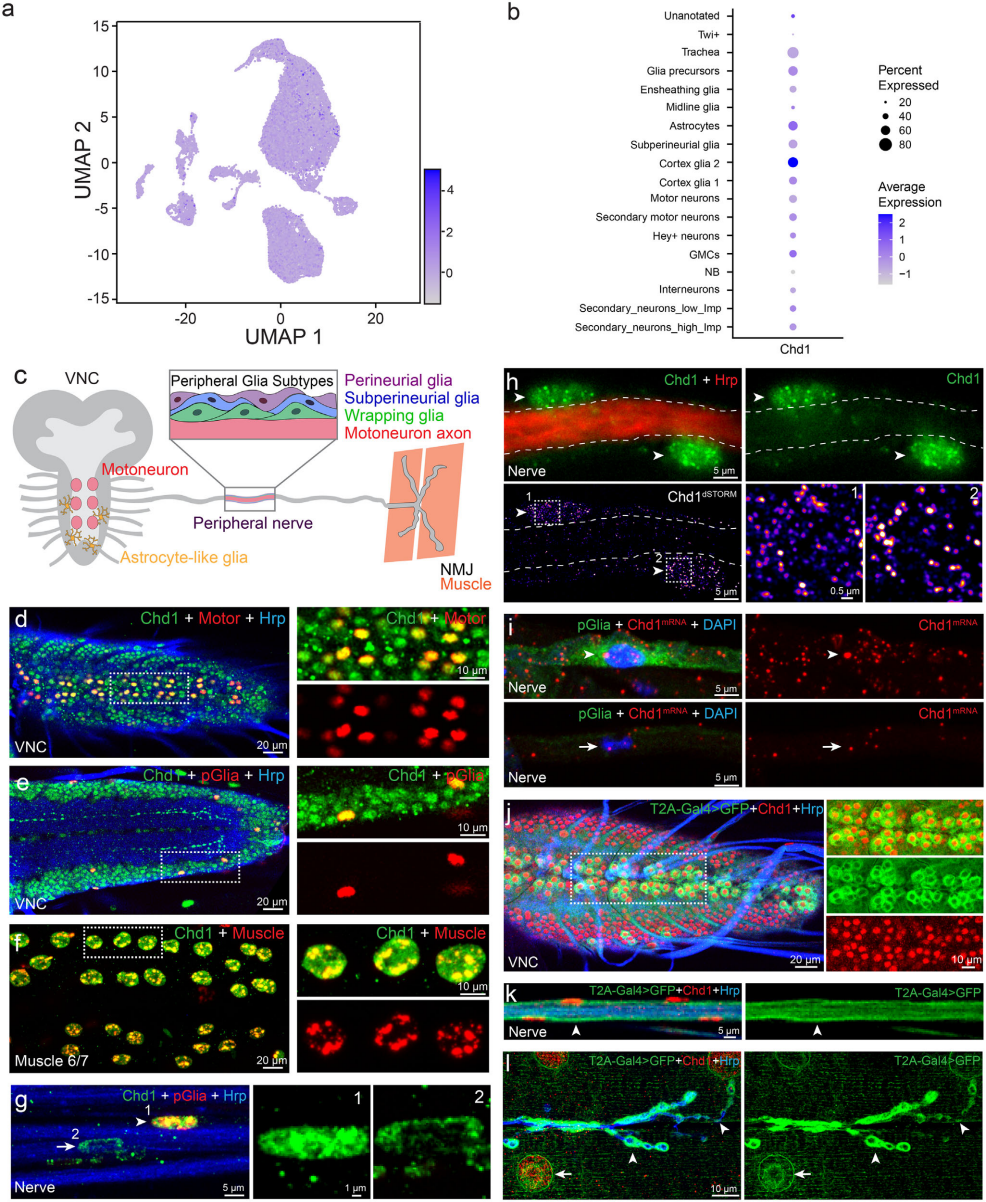
*Chd1* is expressed in motoneurons, muscle, and glia in *Drosophila* larvae. (a) Expression of *Chd1* in the *Drosophila* larval ventral nerve cord visualized by UMAP plots. Expression levels are indicated in blue [134]. (b) Expression of *Chd1* in different cell types in larval ventral nerve cord [134]. Percent of cells and average expression level are shown. (c) Schematic of the *Drosophila* larval ventral nerve cord (VNC, gray), motoneurons (red), astrocyte‐like glia (yellow), peripheral nerves and peripheral glial subtypes, perineurial glia (purple), subperineurial glia (blue), and wrapping glia (green), as well as the neuromuscular junction (NMJ, muscle in orange). (d–f) Confocal images of motoneurons (d, motor) and perineurial glia (e, pGlia) in the ventral nerve cord (VNC) and muscle (f, muscle) in third instar larvae. *UAS‐RedStinger.nls* is expressed in the nuclei of motoneurons (d, *OK371‐Gal4>UAS‐RedStinger.nls*, red), perineurial glia (e, *NP6293‐Gal4>UAS‐RedStinger.nls*, red), or muscles (f, *MHC‐Gal4>UAS‐RedStinger.nls*, red) to label specific cell types. Chd1 protein (green) and neuronal membranes (HRP, blue) are shown. Regions outlined by white boxes in the left panels are shown at higher magnification in the right panels. (g) Confocal images showing perineurial glial nuclei (pGlia, *NP6293‐Gal4>UAS‐RedStinger.nls*, red) and Chd1 protein (green) on peripheral nerves (HRP, blue) in third instar larvae. Perineurial glial nuclei (arrowhead) and other glial subtypes (arrow) are shown at higher magnification in panels 1 and 2. (h) Epifluorescence (top panels) and dSTORM super‐resolution microscopy images (bottom panels) of peripheral nerves from *wild‐type* third instar larvae immunolabeled for Chd1 protein (green) and neuronal membranes (HRP, red, outlined with dashed lines). Subnuclear localization of Chd1 (arrowheads) is shown at higher magnification in panels 1 and 2. (i) Dual in situ hybridization for *Chd1* mRNA (red) and immunolabeling of perineurial glia (pGlia, green, *NP6293‐Gal4>UAS‐GFP.nls*) in third instar larvae. *Chd1* mRNA is detected in perineurial glial nuclei (arrowheads, top panels), compared to other glial subtypes (non‐green nuclei, arrows, bottom panels) on the peripheral nerve. (j–l) Confocal images showing CD8‐GFP expression in the ventral nerve cord (VNC, j), peripheral glia along the nerve (k), and at presynaptic boutons and in muscle (l) of third instar larvae under the control of *T2A‐Gal4* inserted into a coding intron of the *Chd1* gene (*T2A‐Gal4>UAS‐CD8‐GFP*, green). Chd1 protein (red) and neuronal membranes (HRP, blue) are shown. CD8‐GFP is detected in the nuclei of neurons along the midline of the VNC (j). Regions outlined by white boxes in the left panels are shown at higher magnification in the right panels (j). CD8‐GFP‐positive glia are localized on the surface of the peripheral nerve (arrowheads, k). CD8‐GFP is present in both Ib and Is boutons (arrowheads, l) and in muscle, where it is enriched at the nuclear envelope surrounding Chd1‐labeled nuclei (arrows, l).

To characterize the cell type‐specific expression profile of *Drosophila Chd1*, we first reanalyzed previously published single‐cell RNA sequencing (scRNA‐seq) datasets. Examining *Chd1* expression in the larval ventral nerve cord (VNC), we found that *Chd1* is broadly expressed, with non‐zero expression detected in 15 867 of 31 040 cells, spanning all identified cell types (Figure 2a,b). To evaluate whether this expression pattern is conserved across species and developmental stages, we compared mouse *CHD2* expression (cortex; [76], Figure S2a) with *Drosophila Chd1* expression in adult heads ([77], Figure S2b). In both datasets, *CHD2/Chd1* is broadly expressed across diverse neuronal and glial cell types. This is consistent with prior reports documenting widespread *CHD2* expression in multiple neuronal and glial subtypes in the mouse brain [16].

To further assess *Chd1* expression, we generated and validated an antibody specific to the C‐terminus of Chd1 (Methods and Figure S2c–e). Among the peripheral glial cell types located along the nerve and at the NMJ, including perineurial glia, subperineurial glia, and wrapping glia, our previous work identified a critical role for epigenetic regulation in perineurial glia in mediating PHP [39, 41, 42, 78]. Based on this, we examined Chd1 expression in motoneurons, muscle, and perineurial glia (Figure 2c). Using immunolabeling, we co‐immunolabeled Chd1 with tissue‐specific nuclear markers driven by *OK371‐Gal4* (motoneurons), *NP6293‐Gal4* (perineurial glia), and *MHC‐Gal4* (muscle), each driving *UAS‐Redstinger.nls* in the larval VNC or muscle (Figure 2d–f). We found that Chd1 is robustly expressed in motoneurons (Figure 2d), perineurial glia (Figure 2e), and muscle (Figure 2f) in third instar larvae. The expression of Chd1 in motoneurons within the VNC was further validated using five additional motoneuron‐specific *Gal4* drivers (*OK6‐Gal4* [79, 80], *OK319‐Gal4* [80, 81], *D42‐Gal4* [80], *Ib‐Gal4* (*dHb9‐Gal4*, [80]), and *Is‐Gal4* (*GMR27E09‐Gal4*, [80]); Figure S3). Notably, we also observed Chd1 expression in both classes of motoneurons that innervate muscle 6/7, those forming Ib (*dHb9‐Gal4*, [80]) and Is (*GMR27E09‐Gal4*, [80]) synapses, suggesting that *Chd1* in these motoneurons may contribute to the regulation of synaptic function (Figure S3d,e).

Given that perineurial glia are localized along peripheral nerves, we examined Chd1 expression in perineurial glial nuclei in the periphery and found that Chd1 is enriched in these nuclei (Figure 2g). To further investigate subnuclear localization, we employed direct stochastic optical reconstruction microscopy (dSTORM), a super‐resolution imaging technique. This analysis revealed that Chd1 protein forms punctate structures within the nucleus, consistent with its role as a chromatin remodeler (Figure 2h). To validate *Chd1* mRNA expression in perineurial glia, we performed dual in situ hybridization and immunohistochemistry on peripheral nerves. Perineurial glial nuclei were labeled with GFP (*NP6293‐Gal4>UAS‐GFP.nls*), and individual *Chd1* transcripts were visualized using an RNA‐scope probe. Consistent with protein expression data, *Chd1* mRNA was also enriched in perineurial glia (Figure 2i).

Next, to further determine the cell types that express *Chd1*, we used a *T2A‐Gal4* inserted in a coding‐intron of the endogenous *Chd1* gene ([82]; Figure 2j–l; Figure S4). We expressed *UAS‐CD8‐GFP* under the control of this *T2A‐Gal4* to label the plasma membrane of *Chd1*‐expressing cells (*T2A‐Gal4>UAS‐CD8‐GFP*) and performed co‐immunostaining for Chd1 (Figure 2j–l). CD8‐GFP localized to the cell surface surrounding endogenous Chd1, which is nuclear, confirming that the *T2A‐Gal4* accurately reports *Chd1* expression. We observed CD8‐GFP on the cell surface of neurons in the VNC (Figure 2j), peripheral glia along the nerves (Figure 2k), motoneuron Ib and Is boutons at the NMJ (Figure 2l), and in muscle cells (Figure 2l). Together, these results demonstrate that *Chd1* is expressed in motoneurons, muscle, and peripheral glia, with particularly strong expression in perineurial glia.

### 2.4 *Chd1* Exhibits Cell Type‐Specific Roles in Acute and Chronic PHP

**FIGURE 3:**
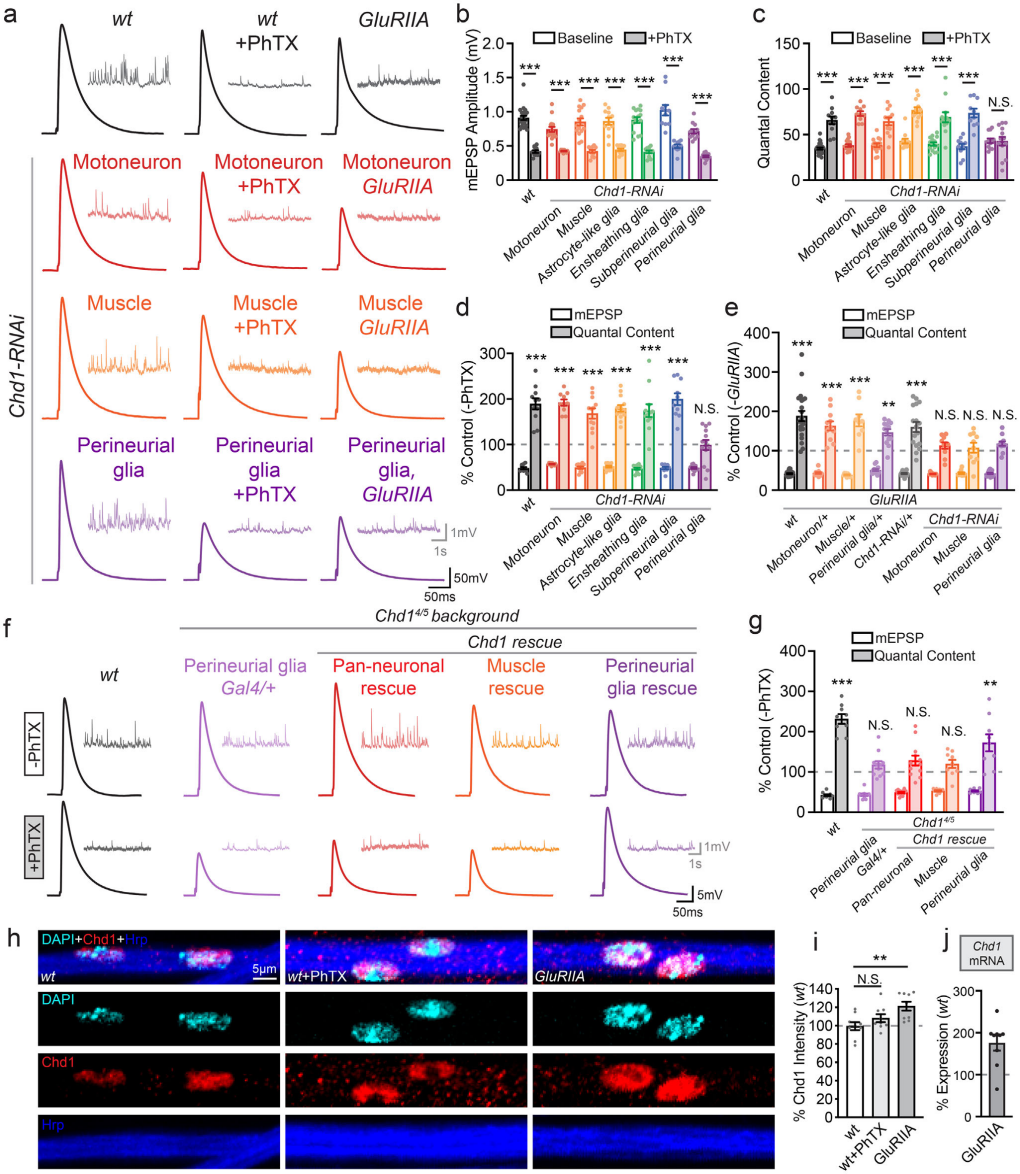
Cell type‐specific function of *Chd1* in the rapid induction and sustained expression of PHP. (a) Representative mEPSP and EPSP traces from *wild‐type* (*wt*) and RNAi‐mediated knockdown of *Chd1* in motoneurons (*OK371‐Gal4>UAS‐Chd1‐RNAi*), muscle (*MHC‐Gal4>UAS‐Chd1‐RNAi*), and perineurial glia (*NP6293‐Gal4>UAS‐Chd1‐RNAi*) in the absence and presence (+PhTX) of philanthotoxin. At far right, *GluRIIA* mutants and *GluRIIA;Chd1‐RNAi* double mutants are shown. (b,c) Non‐normalized raw data for average mEPSP amplitude (b) and presynaptic release (quantal content, c) in the absence (baseline, open bars) and presence (+PhTX, filled bars) of PhTX. Genotypes and sample sizes: *wild‐type* (*wt*, *n* = 22, 11 for −PhTX and +PhTX, respectively); *Chd1* knockdown in motoneurons (*OK371‐Gal4>UAS‐Chd1‐RNAi*, *n* = 17, 10), muscle (*MHC‐Gal4>UAS‐Chd1‐RNAi*, *n* = 15, 12), astrocyte‐like glia (*Alrm‐Gal4>UAS‐Chd1‐RNAi*, *n* = 12, 13), ensheathing glia (*Mz709‐Gal4>UAS‐Chd1‐RNAi*, *n* = 15, 12), subperineurial glia (*Spg‐Gal4>UAS‐Chd1‐RNAi*, *n* = 11, 10), and perineurial glia (*NP6293‐Gal4>UAS‐Chd1‐RNAi*, *n* = 12, 13). Mean ± SEM; ***p < 0.001, N.S. not significant; one‐way ANOVA with Bonferroni test for multiple comparisons. Non‐normalized raw data were used for statistical analysis. (d) Normalized average mEPSP amplitude (open bars) and presynaptic release (quantal content, filled bars), expressed as the percent change in the presence of PhTX compared to the same genotype recorded in the absence of PhTX. Genotypes and statistics are as in (b) and (c). (e) Normalized average mEPSP amplitude (open bars) and presynaptic release (quantal content, filled bars), presented as the percent change in the presence of the *GluRIIA* mutation compared to the same genotype without the mutation. Genotypes and sample sizes: *wild‐type* (*wt*, *n* = 30, 22 for −*GluRIIA* and +*GluRIIA*, respectively), motoneuron *Gal4* control (*OK371‐Gal4/+*, *n* = 10, 11), muscle *Gal4* control (*MHC‐Gal4/+*, *n* = 10, 9), perineurial glial *Gal4* control (*NP6293‐Gal4/+*, *n* = 10, 13), *UAS‐Chd1‐RNAi* control (*Chd1‐RNAi/+*, *n* = 17, 17), motoneuron‐specific knockdown (*OK371‐Gal4>UAS‐Chd1‐RNAi*, *n* = 11, 10), muscle‐specific knockdown (*MHC‐Gal4>UAS‐Chd1‐RNAi*, *n* = 12, 10), and perineurial glial‐specific knockdown (*NP6293‐Gal4>UAS‐Chd1‐RNAi*, *n* = 11, 13). Mean ± SEM; **p < 0.01, ***p < 0.001, N.S. not significant; one‐way ANOVA with Bonferroni test for multiple comparisons. Non‐normalized raw data were used for statistical analysis. (f) Representative mEPSP and EPSP traces from *wild‐type* (*wt*), perineurial glial *Gal4* control (*NP6293‐Gal4/+;Chd1^4/5^*), pan‐neuronal‐specific rescue (*elav^C155^‐Gal4>UAS‐Chd1;Chd1^4/5^*), muscle‐specific rescue (*MHC‐Gal4>UAS‐Chd1;Chd1^4/5^*), and perineurial glial‐specific rescue (*NP6293‐Gal4>UAS‐Chd1;Chd1^4/5^*) in the *Chd1^4/5^* mutant background, in the absence (−PhTX) and presence (+PhTX) of philanthotoxin. (g) Normalized average mEPSP amplitude (open bars) and presynaptic release (quantal content, filled bars), expressed as the percent change in the presence of PhTX compared to the same genotype recorded in the absence of PhTX. Genotypes and sample sizes: *wild‐type* (*wt*, *n* = 16, 9 for −PhTX and +PhTX, respectively), perineurial glial *Gal4* control (*NP6293‐Gal4/+;Chd1^4/5^*, *n* = 13, 11), pan‐neuronal‐specific rescue (*elav^C155^‐Gal4>UAS‐Chd1;Chd1^4/5^*, *n* = 11, 12), muscle‐specific rescue (*MHC‐Gal4>UAS‐Chd1;Chd1^4/5^*, *n* = 10, 9), and perineurial glial‐specific rescue (*NP6293‐Gal4>UAS‐Chd1;Chd1^4/5^*, *n* = 9, 9). Mean ± SEM; **p < 0.01, ***p < 0.001, N.S. not significant; one‐way ANOVA with Bonferroni test for multiple comparisons. Non‐normalized raw data were used for statistical analysis. (h) Representative confocal images of peripheral glial nuclei (DAPI, cyan), Chd1 (red), and neuronal membrane (HRP, blue) are shown for *wild‐type* (*wt*), *wild‐type* treated with PhTX for 30 min (*wt*+PhTX), and *GluRIIA* mutants. (i) Quantification of average Chd1 fluorescence intensity within DAPI positive glial nuclei. Average Chd1 fluorescence intensities for *wild‐type* (*wt*, *n* = 9 animals), *wild‐type* treated with PhTX for 30 min (*wt*+PhTX, *n* = 9), and *GluRIIA* mutants (*GluRIIA*, *n* = 9) are normalized to the *wild‐type* control. Mean ± SEM; **p < 0.01, N.S. not significant; one‐way ANOVA with Bonferroni test for multiple comparisons. Non‐normalized raw data were used for statistical analysis. (j) mRNA expression levels of *Chd1* in *wild‐type* and *GluRIIA* mutants, shown as fold change relative to the housekeeping gene *Rpl32*. mRNA expression was normalized to *wild‐type*. Sample sizes: *n* = 9 per genotype. Mean ± SEM.

To identify the specific cell types in which *Chd1* functions to mediate the rapid induction and long‐term maintenance of PHP, we performed tissue‐specific knockdown of *Chd1* using *UAS‐Chd1‐RNAi* driven by *Gal4* lines targeting distinct cell types. We knocked down *Chd1* in motoneurons (*OK371‐Gal4*), muscle *(MHC‐Gal4*), two key glial subtypes in the brain, astrocyte‐like glia [83, 84] (*Alrm‐Gal4*) and ensheathing glia [85, 86] (*Mz709‐Gal4*), and two major glial subtypes in the periphery, subperineurial glia [78] (*Spg‐Gal4*) and perineurial glia [41, 87] (*NP6293‐Gal4*; Figure  3, Figures S5–S9). Knockdown efficiency of the *UAS‐Chd1‐RNAi* was validated by immunohistochemistry and confocal imaging (Figure S5). We first examined the rapid induction of PHP using electrophysiological recordings. When *Chd1* was knocked down in motoneurons, muscle, astrocyte‐like glia, or subperineurial glia, presynaptic neurotransmitter release increased significantly following PHP induction with PhTX, and EPSP amplitudes recovered to baseline. These results indicate that *Chd1* is not required in these cell types for acute PHP (Figure 3a–d; Figure S6g). In contrast, perineurial glia‐specific knockdown of *Chd1* (*NP6293‐Gal4>UAS‐Chd1‐RNAi*) completely blocked the PhTX‐induced increase in quantal content (Figure 3a–d; Figure S6g). Knockdown of *Chd1* in ensheathing glia produced a significant increase in quantal content in the presence of PhTX, but EPSP amplitude was not fully restored to baseline (Figure S6g). Notably, perineurial glia‐specific *Chd1* knockdown markedly reduced EPSP amplitude after PhTX treatment, supporting the conclusion that although *Chd1* in ensheathing glia may contribute modestly, *Chd1* functions primarily in perineurial glia to regulate acute PHP (Figure S6g).

As a critical control, all heterozygous *Gal4* driver lines exhibited normal baseline transmission and acute PHP responses (Figure S6a–d), and baseline synaptic transmission was largely unaffected across all *Chd1‐RNAi* knockdown conditions compared to *wild‐type* (Figure S6e–g). To further evaluate the cell type‐specific role of *Chd1* in regulating acute PHP, we knocked down *Chd1* in motoneurons using an additional motoneuron‐specific *Gal4* driver, *D42‐Gal4*. PhTX‐induced acute PHP remained intact under the motoneuron‐specific knockdown condition (*D42‐Gal4>UAS‐Chd1‐RNAi*; Figure S7). Together, these results demonstrate that *Chd1* is specifically required in perineurial glia, but not in motoneurons or muscle, for the rapid induction of PHP.

Next, we assessed the cell type‐specific requirement for *Chd1* in the long‐term maintenance of PHP by driving *UAS‐Chd1‐RNAi* in motoneurons (*OK371‐Gal4*), muscle (*MHC‐Gal4*), and perineurial glia (*NP6293‐Gal4*) in the *GluRIIA* mutant background. When *Chd1* was knocked down in any of these cell types, quantal content failed to increase, resulting in a significant reduction in EPSP amplitude in the *GluRIIA;Chd1‐RNAi* double mutants compared to *Chd1‐RNAi* knockdown alone (Figure 3a,e; Figure S8). These results indicate that *Chd1* is required in motoneurons, muscle, and perineurial glia for the sustained expression of PHP. As a critical control, we examined *GluRIIA* double mutants carrying either heterozygous *Gal4* drivers (without *UAS‐RNAi*) or heterozygous *Chd1‐RNAi* (without *Gal4* drivers). In both cases, quantal content increased significantly, and EPSP amplitude was restored toward baseline levels (Figure 3e; Figure S8), confirming that PHP remains intact in these conditions. These controls validate that the PHP deficits observed in the double mutants are specifically due to *Chd1* knockdown in targeted cell types, rather than nonspecific effects of *Gal4* drivers or leaky RNAi expression. Furthermore, we knocked down *Chd1* in motoneurons using the additional *D42‐Gal4* driver in the *GluRIIA* mutant background and assessed chronic PHP. We found that *Chd1* is required in motoneurons for the long‐term expression of PHP (Figure S9). Together, these findings reveal distinct, cell‐type‐specific roles for *Chd1* in the temporal regulation of PHP: it is required specifically in perineurial glia for acute PHP, whereas long‐term maintenance of PHP requires its function in perineurial glia, motoneurons, and muscle.

Finally, to further validate our finding that *Chd1* is specifically required in perineurial glia for the rapid induction of PHP, we performed tissue‐specific rescue experiments. We generated a *UAS‐Chd1* transgenic line (see Methods for details) and overexpressed *Chd1* pan‐neuronally (*elav^C155^‐Gal4*), in muscle (*MHC‐Gal4*), or in perineurial glia (*NP6293‐Gal4*) in the homozygous *Chd1^4/5^* mutant background. Only *Chd1* expression in perineurial glia rescued PHP in *Chd1^4/5^* mutants, as indicated by a significant increase in quantal content and restoration of EPSP amplitude to baseline levels (Figure 3f,g; Figure S10a–c). The ATPase activity of *Chd1* is essential for its chromatin remodeling function [88, 89]. To test whether *Chd1* indeed acts as a chromatin remodeler in regulating PHP, we generated an ATPase‐inactive version of *UAS‐Chd1* in which lysine 559 is mutated to arginine and tagged with HA (*UAS‐Chd1^KR^‐3HA*; [88, 89] and see Methods for details). We found that perineurial glial expression of *wild‐type UAS‐Chd1‐3HA* in the *Chd1^4/5^* mutant background rescued acute PHP, whereas the ATPase‐deficient form (*UAS‐Chd1^KR^‐3HA*) failed to rescue the PHP deficits (Figure S10d–h). These results indicate that the ATPase‐dependent chromatin remodeling activity of *Chd1* is required for synaptic homeostatic plasticity. In summary, these tissue‐specific knockdown and rescue experiments strongly support the conclusion that *Chd1* functions in distinct cell types to regulate different temporal phases of PHP. Our results provide the first direct evidence that glial *Chd1*, particularly in perineurial glia, plays a critical role in mediating homeostatic synaptic plasticity.

### 2.5 *Chd1* Expression is Upregulated in Chronic PHP

The Spt‐Ada‐Gcn5 acetyltransferase and deubiquitinase (SAGA) complex is essential for histone acetylation and gene transcription [90]. Our previous work demonstrated that the SAGA complex functions in perineurial glia to control both the rapid induction and long‐term expression of homeostatic plasticity [41]. Consistent with this role, the open chromatin markers H3K9Ac (acetylation of lysine 9 site on H3) and H3K14Ac (acetylation of lysine 14 site on H3) are significantly increased in peripheral glia during chronic PHP, a process mediated by the SAGA complex [41]. We next asked whether peripheral glia can detect changes arising from muscle perturbations, such as glutamate receptor inhibition, and dynamically regulate *Chd1* expression across the rapid induction and chronic phases of PHP.

To address this, we examined *Chd1* expression in peripheral glia following 30 min of PhTX treatment and in *GluRIIA* mutants using Chd1 immunolabeling and confocal imaging (Figure 3h,i). Chd1 intensity showed an upward trend but did not reach statistical significance after 30 min of PhTX treatment. In contrast, Chd1 protein expression increased significantly by approximately 20% in peripheral glia of *GluRIIA* mutants (Figure 3h,i). To test whether *Chd1* is transcriptionally regulated during chronic PHP, we quantified *Chd1* mRNA levels in *GluRIIA* mutants using quantitative PCR (qPCR). Tissues were collected from third‐instar larval VNCs and peripheral nerves, matching the developmental stage used in our electrophysiology and imaging experiments. We found a ∼80% increase in *Chd1* transcript levels in *GluRIIA* mutants compared with *wild‐type* controls (Figure 3j). Together, these results suggest that peripheral glia can detect muscle‐derived changes and that *Chd1* expression is dynamically regulated during chronic PHP.

We next asked whether *Chd1* acts in concert with the SAGA complex in glia to control PHP. We overexpressed an essential SAGA component *Ada2b* in perineurial glia in the *Chd1* homozygous mutants (*Chd1^4/5^,NP6293‐Gal4>UAS‐Ada2b‐3HA*) and tested acute induction of PHP. We found that perineurial glial expression of *Ada2b* is not sufficient to rescue the PHP deficit in the *Chd1^4/5^* mutant (Figure S10d–h). Next, we tested for genetic interactions between *Chd1* and two SAGA components, *Gcn5* and *Ada2b* ([41, 91, 92]; Figure S11). Heterozygous mutations in *Chd1*, *Gcn5*, or *Ada2b* alone had no effect on the rapid induction of PHP; all three heterozygous mutants exhibited a significant increase in quantal content in the presence of PhTX (Figure S11a–e). The Chd1^5^/+;Gcn5^Q186*^/+ trans‐heterozygous mutants also showed normal PHP, whereas the *Chd1^5^/+;Ada2b^1^/+* trans‐heterozygous mutants displayed impaired PHP (Figure S11a–e). The absence of a consistent genetic interaction across both SAGA components suggests that *Chd1* may function in a parallel pathway or may act together with specific SAGA subunits rather than with the SAGA complex as a whole.

To investigate whether *Chd1* directly interacts with *Ada2b*, we performed immunoprecipitation followed by western blotting, but did not detect a physical interaction between the two proteins (Figure S11f). However, given that the SAGA complex contains roughly twenty subunits, we cannot completely rule out potential interactions with other components. Finally, to test whether *Chd1* operates downstream of the SAGA complex, we examined *Chd1* mRNA expression in *Ada2b* and *Gcn5* homozygous mutants using qPCR. We observed an approximately 20% reduction in *Chd1* expression in *Ada2b* mutants, whereas *Chd1* expression increased by ∼60% in *Gcn5* mutants (Figure S11g). These inconsistent changes suggest that *Chd1* expression is not reliably regulated by the SAGA complex. In summary, these findings suggest that *Chd1* likely functions independently of the SAGA complex to regulate gene expression and PHP. Nevertheless, *Chd1* and the SAGA pathway may share overlapping downstream effectors, as indicated by the genetic interaction observed with *Ada2b*.

### 2.6 *Chd1* is Required for Normal Larval Motor Function but Not NMJ Morphogenesis

**FIGURE 4:**
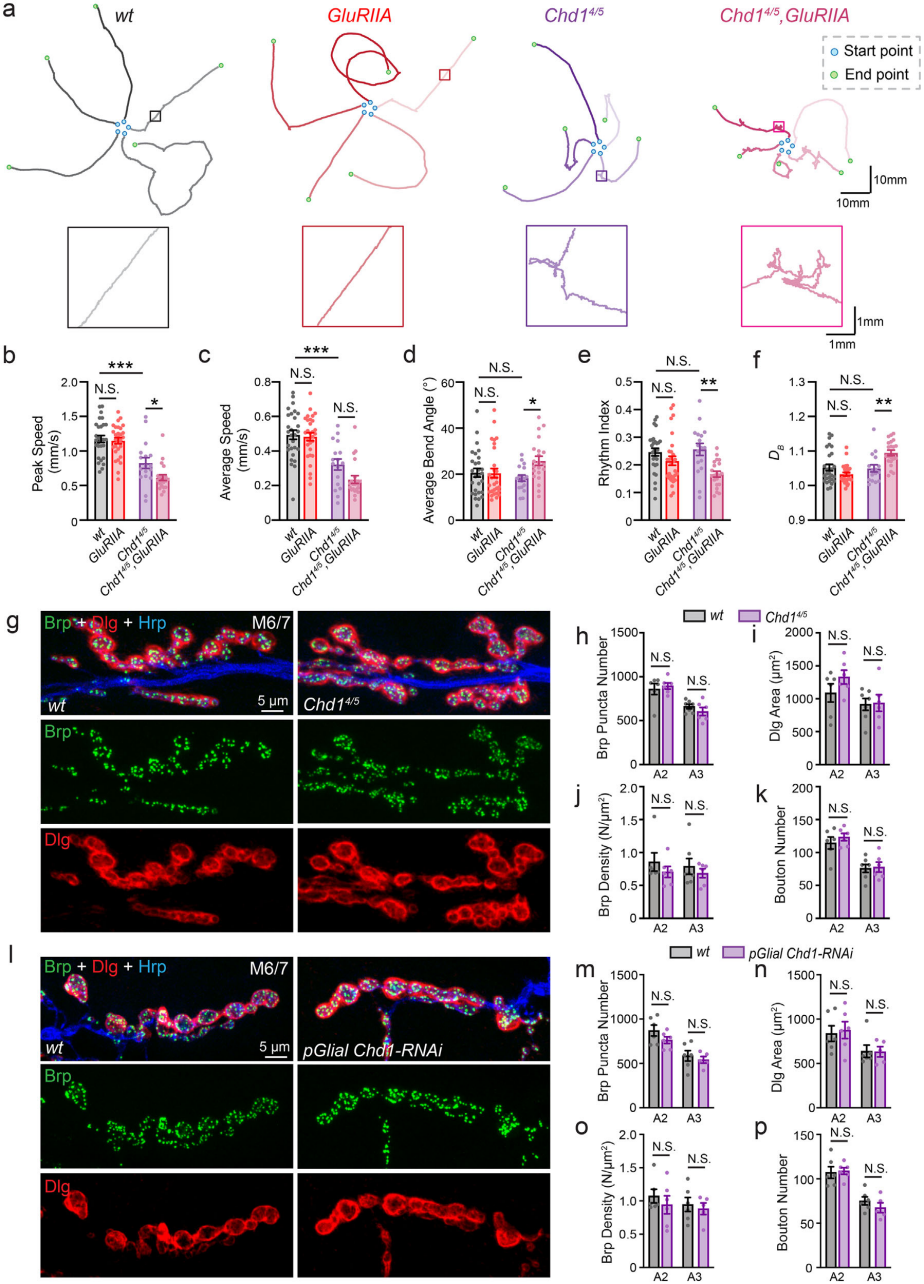
Loss of *Chd1* impairs crawling behavior but does not affect gross synapse morphology. (a) Representative larval crawling traces from *wild‐type* (*wt*), *GluRIIA* mutants, *Chd1^4/5^* mutants, and *Chd1^4/5^,GluRIIA* double mutants (upper panels). Start points (blue dots) and endpoints (green dots) are marked for each crawling trace. Movement paths are shown at higher magnification in the lower panels. (b–f) Quantification of larval crawling behavior: peak speed (b), average speed (c), average bend angle (d), rhythm index (e), and fractal dimension (box‐counting D_B_, f) in *wild‐type* (*wt*, *n* = 28), *GluRIIA* mutants (*n* = 29), *Chd1^4/5^* mutants (*n* = 17), and *Chd1^4/5^,GluRIIA* double mutants (*n* = 21). Mean ± SEM; *p < 0.05, **p < 0.01, ***p < 0.001, N.S. not significant; one‐way ANOVA with Bonferroni test for multiple comparisons. (g) Representative confocal images of the NMJ at muscle 6/7 (abdominal segment 2) in *wild‐type* (*wt*) and *Chd1^4/5^* mutants. NMJs were immunolabeled with anti‐Bruchpilot (Brp, green), anti‐Discs large (Dlg, red), and neuronal membrane marker (HRP, blue). (h–k) Quantification of NMJ structure at muscle 6/7 in abdominal segment 2 (A2) and segment 3 (A3) in *wild‐type* (*wt*) and *Chd1^4/5^* mutants: total number of presynaptic Brp puncta (h), total postsynaptic Dlg area (i), Brp density (Brp puncta/Dlg area, j), and total number of synaptic boutons (k). Sample sizes: *wild‐type* (*wt*, *n* = 6, 7 synapses for A2 and A3, respectively) and *Chd1^4/5^* mutants (*n* = 6, 6 synapses for A2 and A3, respectively). Mean ± SEM; N.S. not significant; unpaired Student's *t*‐test comparing *wild‐type* and *Chd1^4/5^* mutant NMJs within the same muscle segment. (l) Representative confocal images of the NMJ at muscle 6/7 (abdominal segment 2) in *wild‐type* (*wt*) and perineurial glia‐specific knockdown of *Chd1* (*pGlial Chd1‐RNAi*, *NP6293‐Gal4>UAS‐Chd1‐RNAi*). NMJs were immunolabeled with anti‐Bruchpilot (Brp, green), anti‐Discs large (Dlg, red), and neuronal membrane marker (HRP, blue). (m–p) Quantification of NMJ structure in *wild‐type* (*wt*) and perineurial glia‐specific *Chd1* knockdown animals (*pGlial Chd1‐RNAi*, *NP6293‐Gal4>UAS‐Chd1‐RNAi*): total number of presynaptic Brp puncta (m), total postsynaptic Dlg area (n), Brp density (Brp puncta/Dlg area, o), and total number of synaptic boutons (p) at muscle 6/7 in abdominal segment 2 (A2) and segment 3 (A3). Sample sizes: *wild‐type* (*wt*, *n* = 6, 6 synapses for A2 and A3, respectively) and *NP6293‐Gal4>UAS‐Chd1‐RNAi* (*n* = 6, 5 synapses for A2 and A3, respectively). Mean ± SEM; N.S. not significant; unpaired Student's *t*‐test comparing *wild‐type* and *Chd1‐RNAi* knockdown groups within the same muscle segment.

Given that *Chd1* is required for homeostatic plasticity at the *Drosophila* NMJ and that haploinsufficiency mutations in *CHD2* are associated with hypotonia and motor dysfunction in human patients [56], we asked whether impairments in the long‐term maintenance of PHP are linked to motor function deficits in *Drosophila* larvae. We recorded larval crawling behavior using a commodity camera and quantified multiple parameters in third instar larvae, including average speed, peak speed, stride frequency, bend angle, rhythm index, and movement complexity (assessed by fractal dimension, Figure 4a–f; Figure S12; see Methods for details). *GluRIIA* mutants exhibited motor behavior comparable to *wild‐type* controls, with no significant differences across any measured parameter. These results suggest that PHP effectively compensates for glutamate receptor disruption in the muscle, thereby preserving motor output (Figure 4a–f; Figure S12; see also [93]).

In contrast, *Chd1^4/5^* homozygous mutants displayed significantly reduced peak and average crawling speeds, indicating impaired motor performance (Figure 4b,c). Notably, *Chd1^4/5^,GluRIIA* double mutants showed a further reduction in crawling peak speed and rhythm index compared to *Chd1^4/5^* single mutants (Figure 4b,e). Additionally, *Chd1^4/5^,GluRIIA* double mutants exhibited increased bend angle and elevated fractal dimension values, indicating not only slower movement but also more irregular and complex motor trajectories, possibly reflecting disrupted motor coordination and increased occurrence of involuntary muscle contractions or aberrant neuromuscular activity (Figure 4d,f). Together, these findings suggest that disruption of the long‐term maintenance of PHP in the *Chd1^4/5^,GluRIIA* mutant is linked to exacerbated motor function deficits in *Drosophila*.

Previous studies have shown that mammalian *CHD2* is critical for embryonic neurogenesis and interneuron development [49, 51, 52]. This prompted us to ask whether the PHP deficits and motor impairments observed in *Drosophila Chd1* homozygous mutants result from defects in glutamatergic synapse morphogenesis at the NMJ. Additionally, to determine whether *Chd1* expression in perineurial glia is required for synapse development, we examined NMJ morphology following *Chd1* knockdown specifically in perineurial glia using the perineurial glial *Gal4* driver (*NP6293‐Gal4*). We immunolabeled presynaptic active zones with Bruchpilot (Brp, green), postsynaptic scaffolding proteins with Discs‐large (Dlg, red), and the neuronal membrane with HRP (blue) at the NMJ on muscles 6/7 in abdominal segments A2 and A3 (Figure 4g–p). Confocal imaging revealed no significant differences in synapse morphology between *Chd1^4/5^* homozygous mutants (Figure 4g–k) or *NP6293‐Gal4>UAS‐Chd1‐RNAi* knockdown animals (Figure 4l–p) compared to *wild‐type*. Quantification of Brp puncta (Figure 4h,m), postsynaptic Dlg area (Figure 4i,n), active zone density (Figure 4j,o), and total bouton number per synapse (Figure 4k,p) showed no significant differences across genotypes. These results indicate that loss of *Chd1* does not cause major defects in NMJ synapse morphogenesis. Thus, the PHP deficits and motor function impairment observed in *Chd1* mutants are not attributable to developmental abnormalities in synaptic structure.

### 2.7 *Chd1* is Required for the Compensatory Presynaptic Calcium Influx during PHP

**FIGURE 5:**
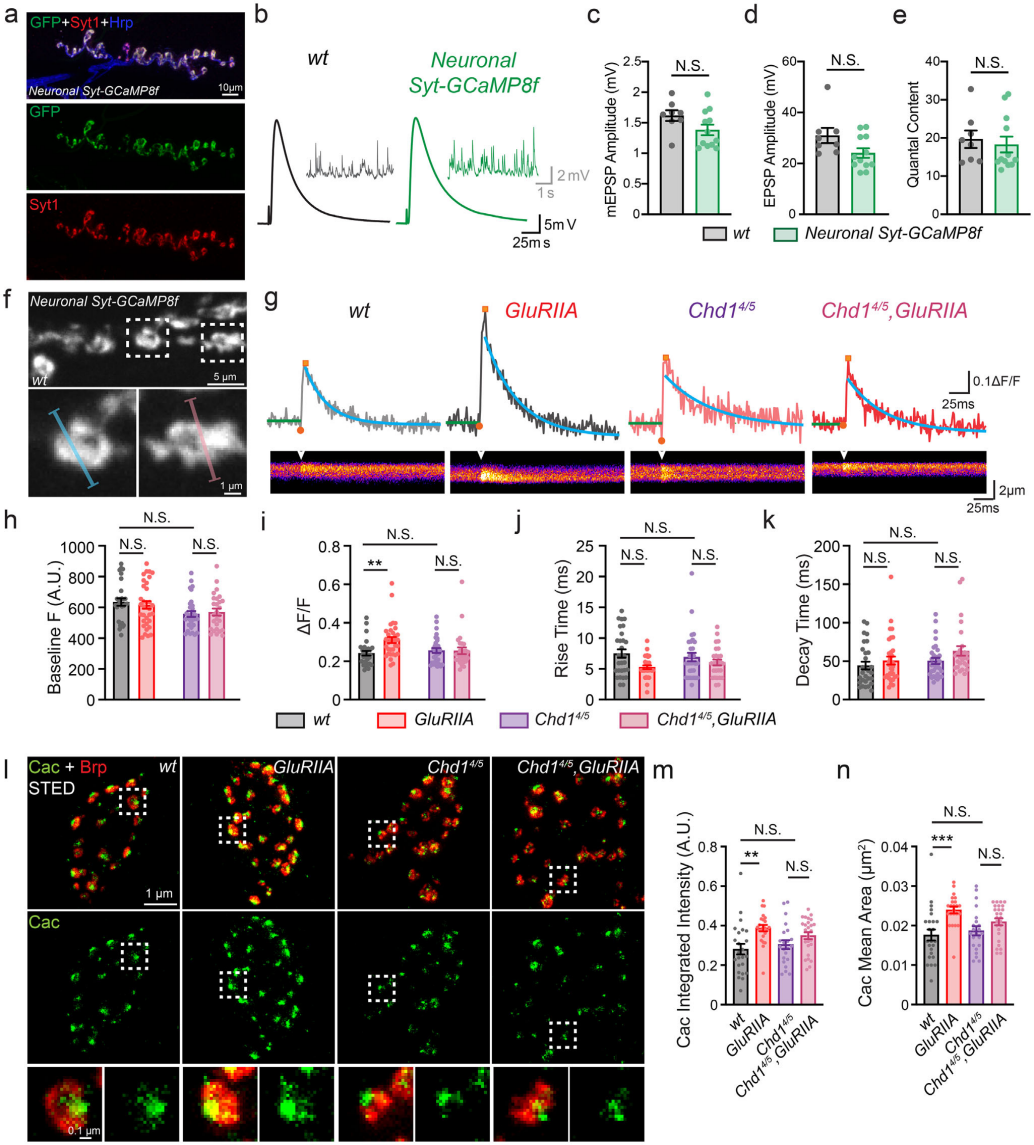
*Chd1* is required for the increase in presynaptic calcium transients and calcium channel abundance during chronic PHP. (a) Representative confocal images of the *Drosophila* NMJ expressing *UAS‐Syt‐GCaMP8f* in neurons (*elav^C155^‐Gal4>UAS‐Syt‐GCaMP8f*). NMJs were immunolabeled with anti‐GFP (GFP, green), anti‐Synaptotagmin1 (Syt1, red), and neuronal membrane marker (HRP, blue). (b) Representative mEPSP and EPSP traces from *wild‐type* (*wt*) and neuronal expression of *UAS‐Syt‐GCaMP8f* (*elav^C155^‐Gal4>UAS‐Syt‐GCaMP8f*). (c–e) Average mEPSP amplitude (c), EPSP amplitude (d), and quantal content (e) in *wild‐type* (*wt*, *n* = 8) and neuronal expression of *UAS‐Syt‐GCaMP8f* (*elav^C155^‐Gal4>UAS‐Syt‐GCaMP8f*, *n* = 12). Mean ± SEM; N.S. not significant; Unpaired Student's *t*‐test comparing *wild‐type* and neuronal expression groups. (f) Representative confocal images of the *Drosophila* NMJ expressing *UAS‐Syt‐GCaMP8f* in neurons (*elav^C155^‐Gal4>UAS‐Syt‐GCaMP8f*). Confocal line scans across single Ib boutons (shown at higher magnification in lower panels) are indicated by blue and red lines. (g) Representative traces of single action potential (AP)‐evoked, spatially averaged calcium transients obtained by line scans across a single 1b bouton from *wild‐type* (*wt*), *GluRIIA* mutants, *Chd1^4/5^* mutants, and *Chd1^4/5^,GluRIIA* double mutants expressing *UAS‐Syt‐GCaMP8f* (*elav^C155^‐Gal4>UAS‐Syt‐GCaMP8f*). The start point (orange circle), peak (orange box), and decay phase (blue line, exponential fit) of the calcium transient are shown. (h–k) Quantification of *Syt‐GCaMP8f* calcium imaging data: average baseline fluorescence (h), peak amplitude of single AP‐evoked calcium transients (ΔF/F, i), rise time (j), and decay time (k) for *wild‐type* (*wt*, *n* = 29), *GluRIIA* mutants (*n* = 33), *Chd1^4/5^* mutants (*n* = 31), and *Chd1^4/5^,GluRIIA* double mutants (*n* = 27). Mean ± SEM; **p < 0.01, N.S. not significant; Kruskal–Wallis test with Dunn's test for multiple comparisons. (l) Representative STED super‐resolution microscopy images of the endogenous calcium channel Cac^sfGFP^ (green) and presynaptic active zones labeled with anti‐Bruchpilot (Brp, red) from individual 1b boutons in *wild‐type* (*wt*), *GluRIIA* mutants, *Chd1^4/5^* mutants, and *Chd1^4/5^,GluRIIA* mutant animals. Higher magnification images of individual active zones are shown in lower panels. (m,n) Quantification of calcium channel content at active zones: average Cac integrated intensity (m) and Cac mean area (n) within Brp‐positive presynaptic active zones in *wild‐type* (*wt*, *n* = 24), *GluRIIA* mutants (*n* = 23), *Chd1^4/5^* mutants (*n* = 22), and *Chd1^4/5^,GluRIIA* double mutants (*n* = 26). Mean ± SEM; **p < 0.01, ***p < 0.001, N.S. not significant; one‐way ANOVA with Bonferroni test for multiple comparisons.

Next, we performed a series of experiments to investigate the cellular mechanisms underlying *Chd1*‐mediated regulation of PHP. PHP is a calcium‐dependent process that requires an increase in presynaptic calcium influx to enhance neurotransmitter release in response to postsynaptic receptor inhibition [33, 94, 95]. To measure presynaptic calcium influx during PHP, we developed a Synaptotagmin‐tagged calcium sensor, *UAS‐Syt‐GCaMP8f* (see Methods for details; also see [96, 97] for a similar tool). This fast calcium indicator is fused to the synaptic vesicle protein Synaptotagmin, enabling precise localization at neurotransmitter release sites. We expressed *UAS‐Syt‐GCaMP8f* in neurons using *elav^C155^‐Gal4* and confirmed its localization to presynaptic boutons (Figure 5a). To ensure that overexpression of Synaptotagmin or GCaMP8f does not interfere with normal synapse development or function, we examined synaptic transmission and NMJ morphology in neurons expressing *UAS‐Syt‐GCaMP8f*. Synaptic transmission was normal in both low (0.4 mm [Ca^2^^+^]_e_, Figure 5b–e) and physiological (1 mm [Ca^2^^+^]_e_, Figure S13a–d) external calcium, and NMJs exhibited normal active zone number, postsynaptic Dlg area, and bouton number (Figure S13e–i). These results indicate that presynaptic expression of *UAS‐Syt‐GCaMP8f* does not perturb normal synaptic morphology or function.

Next, we used high‐frequency confocal line scans across boutons to measure calcium transients evoked by single action potentials (AP). Presynaptic calcium influx was assessed at Ib boutons in *wild‐type*, *GluRIIA*, *Chd1^4/5^*, and *Chd1^4/5^,GluRIIA* double mutants (Figure 5f–k). As expected, *GluRIIA* mutants showed increased calcium transients, evidenced by elevated ΔF/F amplitude, consistent with functional PHP (Figure 5g,i, [33, 94]). In contrast, *Chd1^4/5^,GluRIIA* double mutants exhibited calcium dynamics indistinguishable from *Chd1^4/5^* single mutants, with no compensatory increase in calcium influx (Figure 5g,i). These findings suggest that the failure of PHP in *Chd1^4/5^,GluRIIA* mutants is due to a lack of the typical upregulation of presynaptic calcium entry observed during chronic PHP. Importantly, baseline fluorescence, rise time, and decay time of calcium transients were comparable in *Chd1^4^* ^/5^ and *wild‐type*, indicating that the observed differences are specific to the compensatory mechanisms of PHP rather than baseline defects in single AP‐evoked calcium transients in the *Chd1^4^* ^/5^ mutant (Figure 5h–k).

Previous studies have shown that PHP involves a significant increase in calcium channel levels at presynaptic release sites [98, 99, 100, 101]. A potential mechanism underlying the impaired increase in presynaptic calcium influx observed in *Chd1^4/5^,GluRIIA* double mutants during chronic PHP is a failure to upregulate presynaptic calcium channel abundance. To investigate whether *Chd1* regulates this process, we utilized a CRISPR knock‐in allele of *Cacophony* (*Cac*), in which the endogenous voltage‐gated calcium channel α1 subunit is tagged with GFP [99]. We performed immunolabeling for Cac‐GFP and the active zone marker Bruchpilot (Brp), followed by stimulated emission depletion (STED) super‐resolution imaging to visualize presynaptic calcium channels (Figure 5l). As expected, Cac‐GFP localized to the center of presynaptic active zones (Figure 5l). Consistent with previous reports, *GluRIIA* mutants showed a significant increase in Cac‐GFP intensity compared to *wild‐type*, reflecting an upregulation of calcium channels during PHP (Figure 5l–n). However, this increase was abolished in *Chd1^4/5^,GluRIIA* double mutants, in which Cac‐GFP intensity was unchanged relative to *Chd1^4/5^* single mutants. These results indicate that *Chd1* is required for the activity‐dependent upregulation of presynaptic calcium channel abundance during chronic PHP. Importantly, baseline Cac‐GFP levels in *Chd1^4^* ^/5^ mutants were comparable to *wild‐type* (Figure 5m,n), consistent with our calcium imaging data showing no deficits in baseline calcium influx (Figure 5g,i). Although we observed a trend toward increased Brp intensity, statistical analysis revealed no significant differences in Brp intensity or active zone area among *wild‐type*, *GluRIIA*, *Chd1^4/5^*, and *Chd1^4/5^,GluRIIA* genotypes (Figure S14), suggesting that active zone structure remains largely unchanged.

### 2.8 *Chd1* is Required for the PHP‐Induced Expansion of the RRP

**FIGURE 6:**
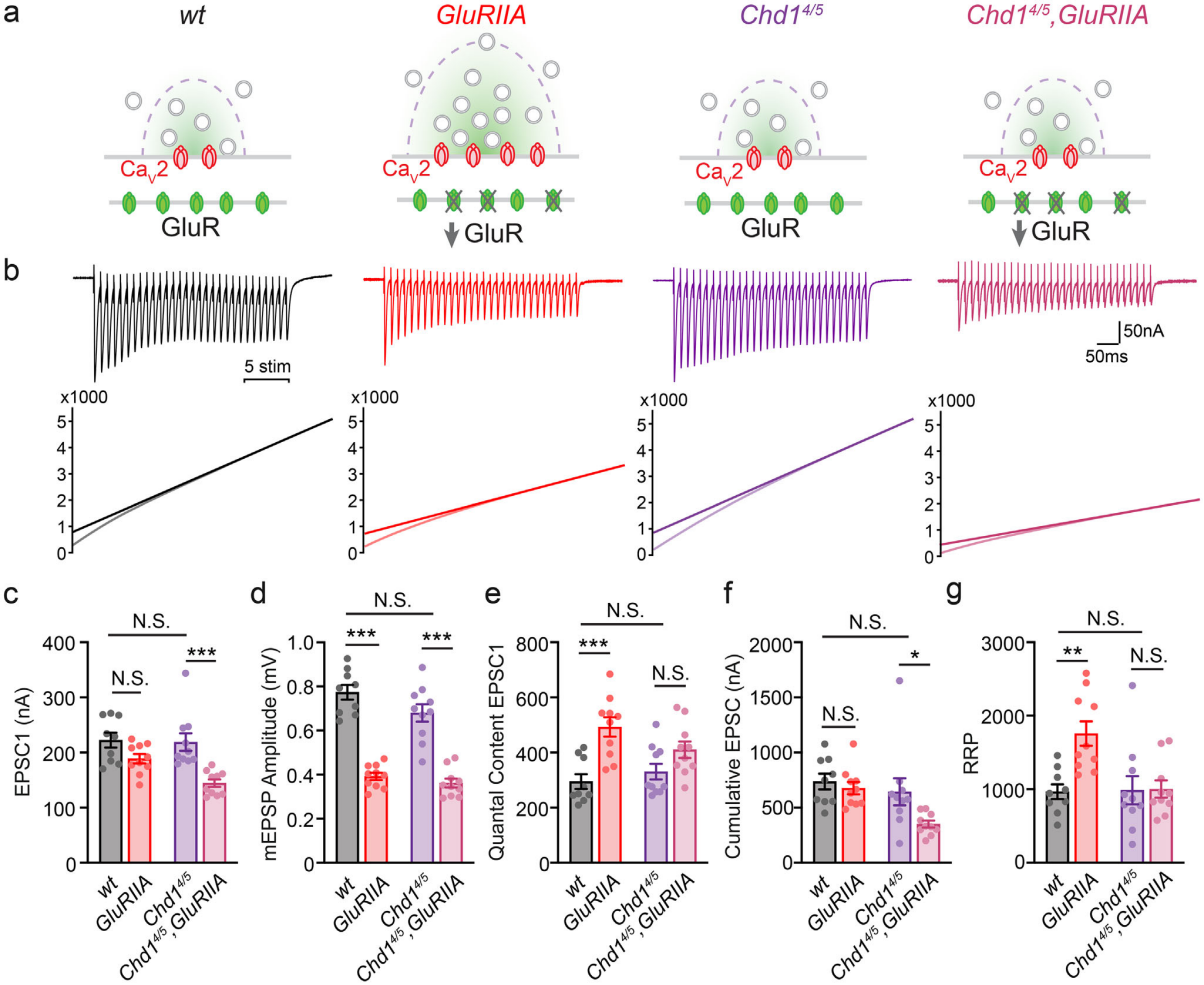
*Chd1* is essential for readily releasable pool expansion in chronic PHP. (a) Schematic illustrating presynaptic calcium influx (green) and RRP size in *wild‐type* (*wt*), *GluRIIA* mutants, *Chd1^4/5^* mutants, and *Chd1^4/5^,GluRIIA* double mutants. Relative changes in presynaptic calcium influx and RRP size observed in mutant genotypes compared to *wild‐type* are indicated. (b) Representative traces of evoked excitatory postsynaptic currents (EPSCs) during 60 Hz stimulus trains in *wild‐type* (*wt*), *GluRIIA* mutants, *Chd1^4/5^* mutants, and *Chd1^4/5^,GluRIIA* double mutants. Cumulative EPSC plots (faded lines) and the back‐extrapolated linear fits of the cumulative phase (solid lines) are shown in the lower panels. (c–g) Quantification of synaptic transmission parameters across genotypes: average amplitude of the first EPSC (EPSC1, c), mEPSP amplitude (d), apparent quantal content calculated from the first EPSC (quantal content EPSC1, e), cumulative EPSC (f), and apparent RRP size (g) for *wild‐type* (*wt*, *n* = 9), *GluRIIA* (*n* = 10), *Chd1^4/5^* (*n* = 10), and *Chd1^4/5^,GluRIIA* (*n* = 10). Mean ± SEM; *p < 0.05, **p < 0.01, ***p < 0.001, N.S. not significant; one‐way ANOVA with Bonferroni test for multiple comparisons.

Another mechanism by which PHP is expressed and maintained is through an increase in the RRP of synaptic vesicles [34, 94, 102]. The RRP consists of vesicles that are immediately available for release upon action potential arrival. When postsynaptic glutamate receptor function is impaired, as in *GluRIIA* mutants, an increase in RRP size serves as a compensatory mechanism to maintain synaptic strength by ensuring more vesicles are available for release (Figure 6a). To assess RRP size during chronic PHP, we stimulated motoneuron axons with high‐frequency trains (60 Hz, 30 stimuli) and performed two‐electrode voltage‐clamp recordings under physiological extracellular calcium concentrations (1.5 mm [Ca^2^^+^]_e_, Figure 6b). This protocol allowed us to measure both synaptic compensation at physiological calcium levels (via first EPSC amplitude and quantal content) and estimate RRP size based on cumulative release during the stimulus train ([34, 102, 103], Figure 6c–g). To determine whether PHP is disrupted in *Chd1^4/5^,GluRIIA* double mutants under physiological calcium conditions, we first examined the amplitude of the first EPSC (EPSC1, Figure 6c). While *GluRIIA* mutants displayed EPSC1 amplitudes comparable to *wild‐type* and a significant increase in apparent quantal content, *Chd1^4/5^,GluRIIA* double mutants showed a marked reduction in EPSC1 amplitude and no significant change in apparent quantal content relative to *Chd1^4/5^* single mutants (Figure 6c–e). These findings indicate a complete block of chronic PHP in the double mutants, consistent with results obtained from current‐clamp recordings under low extracellular calcium conditions (Figure 1l–o).

To assess RRP size in *Chd1^4/5^,GluRIIA* double mutants, we estimated cumulative EPSC amplitude and calculated apparent RRP size by fitting the linear phase of the cumulative EPSC plot and back‐extrapolating to time zero (Figure 6b,f,g). The *y*‐intercept of this extrapolation provides an estimate of the total charge that would have been released if all vesicles in the RRP were released instantaneously [104]. Consistent with previous findings, *GluRIIA* mutants showed no difference in cumulative EPSC amplitude compared to *wild‐type*, but displayed a significant increase in apparent RRP size, reflecting PHP‐dependent expansion of the vesicle pool. In contrast, *Chd1^4/5^,GluRIIA* double mutants exhibited significantly reduced cumulative EPSC amplitude and no change in apparent RRP size relative to the *Chd1^4/5^* single mutant, indicating a failure to expand the RRP during chronic PHP (Figure 6b,f,g). Importantly, *Chd1^4/5^* mutants did not differ from *wild‐type* in baseline RRP size (Figure 6f,g), consistent with the lack of change in presynaptic calcium influx and EPSC amplitude under physiological calcium conditions. These findings support the conclusion that the PHP deficit observed in *Chd1^4/5^* mutants is not due to impairments in basal synaptic transmission but rather reflects a specific disruption in the mechanisms required for PHP expression.

### 2.9 Electrophysiology‐Based Genetic Screen Identifies *Chd1*‐Dependent Signaling Pathways in Acute PHP

**FIGURE 7:**
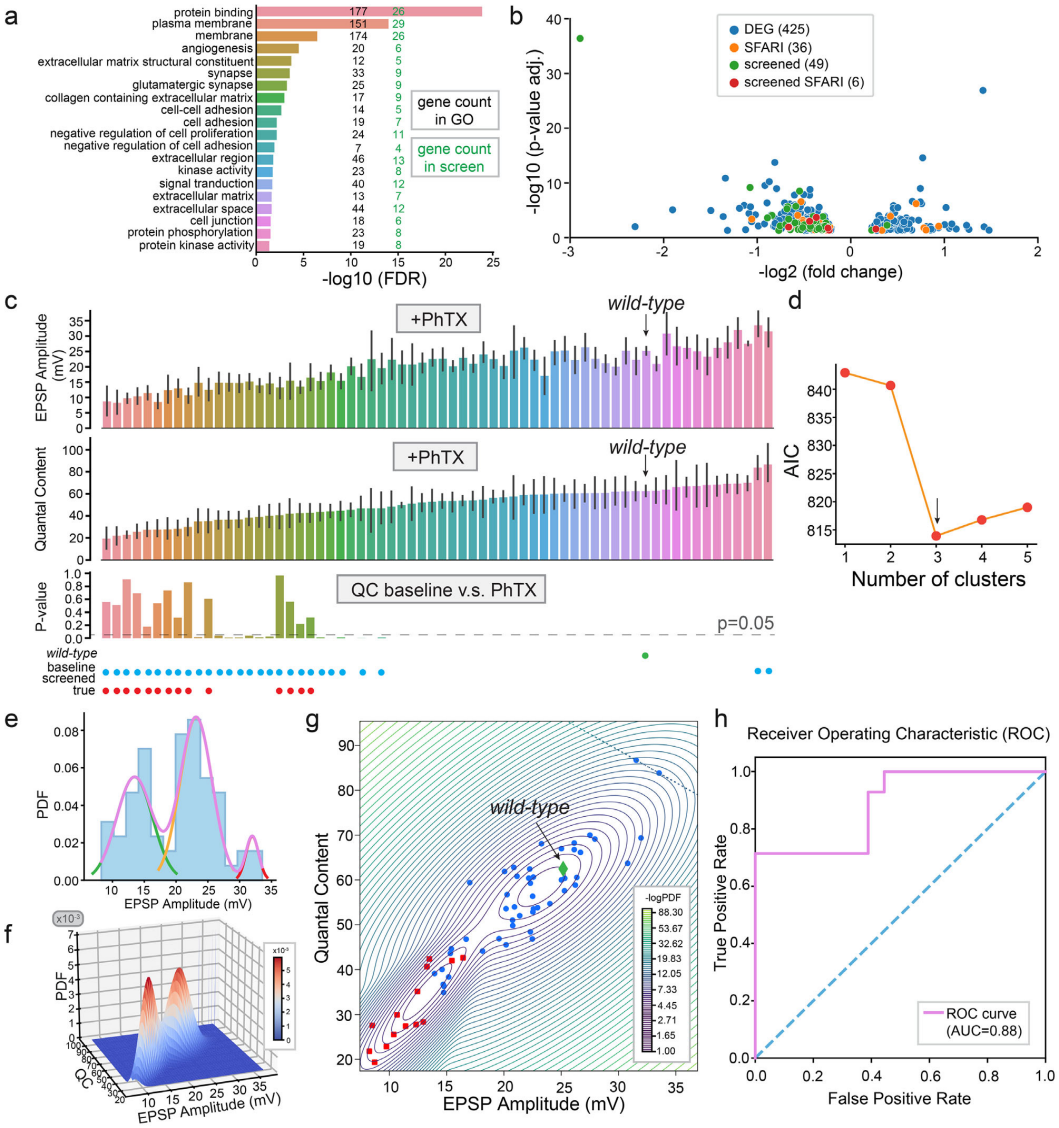
Genetic screen to identify effectors of *Chd1* required for acute PHP. (a) Gene Ontology (GO) terms associated with intercellular communication functions for mouse *CHD2* downstream targets. The total number of genes in each GO category (black) and the number of genes selected for the screen in each category (green) are shown on the right. Note that genes may overlap between GO categories. (b) Transcriptional fold changes and adjusted *p*‐values for differentially expressed genes (DEGs, blue) with intercellular communication functions in *CHD2* knockout mice. Genes selected for the screen (green), SFARI autism risk genes (orange), and SFARI genes included in the screen (red) are highlighted. (c) Average EPSP amplitude (top panel) and quantal content (middle panel) recorded in the presence of philanthotoxin (+PhTX) in all screened mutants, with *wild‐type* indicated by arrows. The bottom panel shows adjusted *p*‐values for quantal content (QC) in the presence vs. absence of PhTX for 28 candidate hits (baseline screened, blue dots) and *wild‐type* (green dot). Mean ± SEM; unpaired Student's *t*‐test. Mutants showing no significant increase in quantal content upon PhTX treatment, relative to baseline release, indicative of impaired PHP, are labeled as “true hits” (red dots). The threshold *p*‐value = 0.05 is indicated by a dashed line. Sample sizes are shown in Table S1. (d) AIC values plotted against cluster number in the Gaussian Mixture Model (GMM); a minimum AIC is achieved at cluster number *n* = 3 (arrow). (e) Probability density functions (PDF) and GMM fits (colored lines) for average EPSP amplitude across all screened mutants. (f) PDF of average EPSP amplitude and quantal content visualized in three‐dimensional space for all screened mutants. (g) Average EPSP amplitude and quantal content for “true hits” (red dots) and other mutants (blue dots) shown in two‐dimensional space. *Wild‐type* is indicated as a green square on the PDF contour plot. (h) Receiver operating characteristic (ROC) curve showing the true positive and false positive rates for identifying “true hits” using *q_0_* predicted by the GMM model (AUC = 0.88).

To further investigate how *Chd1*‐dependent chromatin remodeling regulates PHP, we initiated an electrophysiology‐based genetic screen to identify downstream signaling pathways regulated by *Chd1* during acute PHP. To identify candidate genes for our screen, we conducted a focused re‐analysis of a previously published RNA‐seq dataset from the P45 mouse hippocampus of heterozygous *CHD2* knockout mice  [52] (Figure  7a,b). While the original analysis characterized broad transcriptional changes associated with *CHD2* haploinsufficiency, our re‐analysis specifically aimed to extract genes relevant to synaptic signaling and homeostatic plasticity. Gene Ontology (GO) enrichment revealed that *CHD2* regulates a wide range of cellular functions, including pathways implicated in neural activity and intercellular communication (Figure  7a). Given our finding that *Chd1* expression in perineurial glia is specifically required for the rapid induction of PHP, we hypothesized that glia‐neuron communication plays a critical role in this process. Accordingly, we prioritized *CHD2*‐regulated genes with known or predicted roles in intercellular signaling, selecting those most likely to influence synaptic function based on their annotated molecular functions and relevance to glia‐neuron communications during acute PHP. From the RNA‐seq dataset, we identified 49 significantly differentially expressed genes (DEGs, *p* < 0.05, Figure  7b), and selected their direct or closest *Drosophila* homologues, yielding a total of 65 genetic mutants for inclusion in our electrophysiology‐based screen (Table S1).

We began by performing electrophysiological recordings in the presence of PhTX, measuring mEPSP amplitude, EPSP amplitude, and quantal content (QC) for each genetic loss‐of‐function mutant (Figure 7c). Traditional PHP screens often rely on arbitrary thresholds, such as two standard deviations (SD) from the mean EPSP amplitude under PhTX treatment, to define potential hits before conducting baseline recordings [25, 105, 106]. However, as EPSP amplitudes and quantal content may not follow a normal distribution, using SD as a cutoff lacks statistical justification and may increase the risk of false negatives. To improve the robustness of gene discovery and streamline the screening process, we implemented an unsupervised machine learning approach to identify high‐confidence PHP‐deficient mutants using only data from the +PhTX condition. This approach enables probabilistic classification of hits, reducing the number of required experiments and improving the efficiency of large‐scale genetic screens.

Specifically, we applied a Gaussian Mixture Model (GMM), an unsupervised machine learning algorithm, to cluster the PhTX‐treated dataset in a two‐dimensional space defined by quantal content and EPSP amplitude (see Methods for details). To identify the most likely number of distinct states underlying the homeostatic mechanism, we evaluated models with 1 to 5 clusters and found that the model with *n* = 3 yielded the lowest Akaike Information Criterion (AIC), indicating the best fit to the data (Figure 7d). Based on electrophysiological profiles, we plotted the probability density function (PDF) for all 65 mutants along with *wild‐type* controls and assigned functional interpretations to the clusters: the cluster with the lowest EPSP amplitudes under PhTX likely represents PHP‐deficient mutants (“hit cluster”); the cluster centered near *wild‐type* levels likely includes mutants with intact PHP (“normal cluster”); and the third cluster, characterized by elevated EPSP amplitudes under PhTX, may reflect mutants that overcompensate for synaptic perturbation (“over‐shooter cluster,” Figure 7e–g).

This analysis identified 28 mutants within the hit cluster (highlighted as red dots in Figure 7g), which were prioritized as strong candidates for mediating *Chd1*‐dependent signaling during the rapid induction of PHP. To validate these predictions, we performed electrophysiological recordings in the absence of PhTX to assess baseline mEPSP and EPSP amplitudes in the 28 candidate mutants identified by the GMM model (blue dots in Figure 7c). We found that 14 of these mutants exhibited significantly reduced EPSP amplitude in the presence of PhTX, without significant changes in quantal content compared to baseline (Student's *t*‐test, *p* > 0.05, Figure 7c,g). These genes are specifically required for the rapid induction of PHP, but not for baseline synaptic transmission, and were thus classified as “true hits” (red dots in Figure 7c). By leveraging this machine learning approach, we were able to bypass baseline recordings for 37 of the 65 mutants, reducing experimental workload.

To further assess the predictive performance of the GMM model, we generated a receiver operating characteristic (ROC) curve using the posterior probability of each gene belonging to the hit cluster (*q_0_*). True hits were defined based on electrophysiological recordings under both PhTX‐treated and untreated conditions. The area under the curve (AUC) was 0.88, and all true hits were successfully captured using the conservative threshold of 0.02 on *q_0_*, indicating that the GMM reliably identified PHP regulators using only data from the PhTX condition (Figure  7h, see Methods for details). In addition to improving efficiency and reducing experimental burden, the GMM‐based method also reveals distinct states of homeostatic modulation through clustering and can be readily applied to other genetic screens. GO analysis of the 14 genes required for acute PHP revealed that seven encode extracellular matrix (ECM) proteins (*trol*, *dlp*, *CG31342*, *kon*, *cht11*, *Muc68Ca*, and *Manf*), four encode cell adhesion molecules (*Cad74A*, *sdk*, *kek4*, and *CG1674*), one encodes a protein kinase (*Tie*), one is an adhesion G‐protein‐coupled receptor (GPCR, *Cirl*), and one is involved in autophagy (*Atg1*; Table S1). Given that *Chd1* function in perineurial glia is specifically required for the rapid induction of PHP, these findings suggest that the *Chd1*‐dependent downstream target genes identified in our screen play critical roles in mediating intercellular signaling during acute homeostatic plasticity.

### 2.10 *Drosophila Cadherin 74A*, a Downstream Targe of *Chd1*, is Required for PHP

**FIGURE 8:**
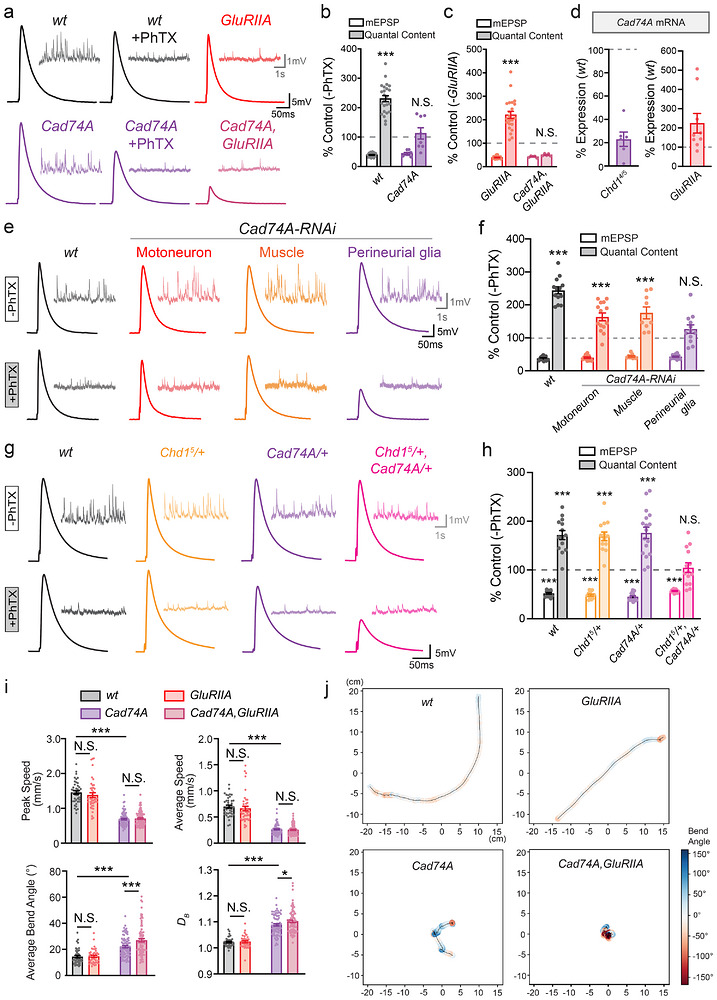
*Cad74A*, a downstream target gene of *Chd1*, is required for PHP. (a) Representative mEPSP and EPSP traces from *wild‐type* (*wt*) and *Cad74A* mutants in the absence and presence (+PhTX) of philanthotoxin. At the far right, traces from *GluRIIA* mutants and *Cad74A,GluRIIA* double mutants are shown. (b,c) Average mEPSP amplitude (open bars) and presynaptic release (quantal content, filled bars). Data are presented as the percent change in the presence of PhTX (b) or homozygous *GluRIIA* mutation (c) relative to the same genotype recorded in the absence of PhTX or *GluRIIA* mutation, respectively. Genotypes and sample sizes: *wild‐type* (*wt*, *n* = 29, 24 for −PhTX and +PhTX, respectively) and *Cad74A* (*n* = 7, 8) in (b); *wild‐type* (*wt*, *n* = 29), *GluRIIA* (*n* = 22), *Cad74A* (*n* = 7), and *Cad74A,GluRIIA* (*n* = 5) in (c). Mean ± SEM; ***p < 0.001, N.S. not significant; one‐way ANOVA with Bonferroni test for multiple comparisons. Non‐normalized raw data were used for statistical analysis. (d) mRNA expression levels of *Cad74A* in *wild‐type* and *Chd1^4/5^* mutants (left panel, *n* = 6 biological replicates), and *wild‐type* and *GluRIIA* mutants (right panel, *n* = 9 biological replicates) shown as fold change relative to the housekeeping gene *Rpl32*. mRNA expression was normalized to *wild‐type*. Mean ± SEM. (e) Representative mEPSP and EPSP traces from *wild‐type* (*wt*) and RNAi‐mediated knockdown of *Cad74A* in motoneurons (*OK371‐Gal4>UAS‐Cad74A‐RNAi*), muscle (*MHC‐Gal4>UAS‐Cad74A‐RNAi*, orange), and perineurial glia (*NP6293‐Gal4>UAS‐Cad74A‐RNAi*) in the absence and presence of PhTX (+PhTX). (f) Average mEPSP amplitude (open bars) and quantal content (filled bars) for genotypes in (e), shown as the percent change in the presence of PhTX relative to the absence (−PhTX). Sample sizes: *wild‐type* (*wt*, *n* = 18, 13 for −PhTX and +PhTX, respectively), knockdown of *Cad74A* in motoneurons (*OK371‐Gal4>UAS‐Cad74A‐RNAi*, *n* = 9, 14), muscle (*MHC‐Gal4>UAS‐Cad74A‐RNA, n* = 12, 9), and perineurial glia (*NP6293‐Gal4>UAS‐Cad74A‐RNAi*, *n* = 13, 12). Mean ± SEM; ***p < 0.001, N.S. not significant; one‐way ANOVA with Bonferroni test for multiple comparisons. Non‐normalized raw data were used for statistical analysis. (g) Representative mEPSP and EPSP traces from *wild‐type* (*wt*), heterozygous *Chd1* mutant (*Chd1^5^/+*), heterozygous *Cad74A* mutant (*Cad74A/+*), and trans‐heterozygous *Chd1^5^/+,Cad74A/+* mutants in the absence and presence of PhTX (+PhTX). (h) Average mEPSP amplitude (open bars) and quantal content (filled bars) for genotypes in (g), shown as the percent change in the presence of PhTX relative to the absence (−PhTX). Sample sizes: *wild‐type* (*wt*, *n* = 15, 14 for −PhTX and +PhTX, respectively), *Chd1^5^/+* (*n* = 20, 13), *Cad74A/+* (*n* = 15, 17), and *Chd1^5^/+,Cad74A/+* (*n* = 18, 14). Mean ± SEM; ***p < 0.001, N.S. not significant; one‐way ANOVA with Bonferroni test for multiple comparisons. Non‐normalized raw data were used for statistical analysis. (i) Quantification of larval locomotion: peak speed (upper left panel), average speed (upper right panel), average bend angle (lower left panel), and fractal dimension (box‐counting D_B_, lower right panel) in *wild‐type* (*wt*, *n* = 42), *GluRIIA* mutants (*n* = 41), *Cad74A* mutants (*n* = 80), and *Cad74A,GluRIIA* double mutants (*n* = 84). Mean ± SEM; *p < 0.05, ***p < 0.001, N.S. not significant; one‐way ANOVA with Bonferroni test for multiple comparisons. (j) Representative larval crawling traces from *wild‐type* (*wt*), *GluRIIA* mutants, *Cad74A* mutants, and *Cad74A,GluRIIA* double mutants. Instantaneous bending angles (bending angle per frame) during crawling are also shown for each genotype.

To further dissect the signaling pathway downstream of *Chd1* in PHP, we selected and validated a candidate gene from our genetic screen. We prioritized a cell adhesion molecule, given its potential role in mediating glia‐neuron communication during PHP. Among these, we focused on *Drosophila Cadherin 74A* (*Cad74A*), a non‐classical cadherin previously implicated in cell adhesion, polarity, and oogenesis  [107, 108, 109]. Although *Cad74A* has not been studied in the context of the nervous system, its predicted adhesive function made it a strong candidate for mediating *Chd1*‐dependent glia‐neuron interactions during homeostatic plasticity. To determine whether *Cad74A* is required for the rapid induction of PHP, we expanded our dataset and applied 20 µm PhTX to both *Cad74A* homozygous mutants and *wild‐type* controls. PhTX reduced mEPSP amplitude by approximately 50% in both genotypes, confirming comparable levels of postsynaptic receptor inhibition (Figure 8a,b; Figure S15). As expected, *wild‐type* animals showed a significant compensatory increase in quantal content in response to PhTX. However, this increase was completely absent in homozygous *Cad74A* mutants, indicating a failure to enhance presynaptic release and confirming that *Cad74A* is essential for the rapid induction of PHP (Figure 8a,b; Figure S15).

Next, we investigated whether *Cad74A* is also required for the long‐term maintenance of PHP. As expected, *GluRIIA* mutants exhibited an approximately 100% increase in quantal content compared to *wild‐type*, consistent with robust homeostatic compensation (Figure 8a,c; Figure S15). However, *Cad74A,GluRIIA* double mutants showed an approximately 70% reduction in EPSP amplitude and a decrease in quantal content compared to *Cad74A* single mutants (Figure 8a,c; Figure S15). These significant changes indicate a severe failure of chronic PHP. Importantly, *Cad74A* mutants did not exhibit significant reductions in EPSP amplitude or quantal content under baseline conditions, in the absence of PhTX treatment or the *GluRIIA* mutation (Figure S15). These findings demonstrate that *Cad74A* is essential for both the rapid induction and long‐term maintenance of PHP. Notably, *Cad74A,GluRIIA* double mutants exhibited more severe synaptic transmission deficits than *Chd1^4/5^,GluRIIA* mutants (Figure  1l–o), suggesting that *Cad74A* plays a critical, and potentially more direct, role in modulating synaptic output. One possible explanation for this difference is variation in *Cad74A* expression between the *Cad74A* and *Chd1^4/5^* mutants.

To test whether *Cad74A* is transcriptionally regulated by *Chd1*, we quantified *Cad74A* mRNA levels in *Chd1^4/5^* mutants using qPCR. Consistent with GO analysis indicating that *Cad74A* is a *Chd1*‐regulated gene involved in intercellular signaling, qPCR revealed an ∼80% reduction in *Cad74A* transcript levels in *Chd1^4/5^* mutants compared to *wild‐type* controls (Figure  8d). These results validate *Cad74A* as a downstream effector of *Chd1* and underscore a conserved role for *Chd1* in regulating cadherin expression. Interestingly, we also observed an ∼100% increase in *Cad74A* transcript levels in *GluRIIA* mutants, consistent with the upregulation of *Chd1* expression in the *GluRIIA* background (Figure 8d; see also Figure 3j). This result suggests that *Chd1* actively regulates downstream target gene expression during chronic PHP.

To test whether the upregulation of *Cad74A* transcript levels in the *GluRIIA* mutant might be associated with increased Chd1 occupancy at the *Cad74A* proximal promoter during dynamic chromatin remodeling, we performed Chromatin Immunoprecipitation followed by qPCR (ChIP‐qPCR) using an antibody specific to Chd1 (Figure S16a). As a positive control, we detected increased Chd1 association with the *HSP70* promoter in heat‐shocked *wild‐type* flies, consistent with previous reports [65]. In contrast, we did not observe a consistent change in Chd1 occupancy at the *Cad74A* proximal promoter in *GluRIIA* mutants (Figure S16a). However, given the variability of the data and the dynamic nature of chromatin regulation, we cannot exclude the possibility that Chd1 regulates *Cad74A* through transient or context‐dependent promoter interactions that are not readily captured by ChIP, or through indirect mechanisms that alter chromatin accessibility. Notably, strong promoter binding by Chd1 has previously been associated with large (10–100‐fold) increases in downstream gene expression [65], whereas the *GluRIIA* mutant shows a more modest ∼100% increase in *Cad74A* mRNA. To further assess whether *Cad74A* is a downstream target of the SAGA complex, we examined *Cad74A* transcript levels in *Gcn5* homozygous mutants. We observed only a mild reduction in *Cad74A* transcript levels in *Gcn5* mutants (Figure S16b). This observation aligns with the lack of consistent genetic interaction between *Chd1* and components of the SAGA complex (Figure S11a–e).

To investigate the cell type‐specific requirement of *Cad74A* in acute PHP, we performed RNAi‐mediated knockdown of *Cad74A* in specific tissues (Figure  8e,f; Figure S17). Based on the hypothesis that *Cad74A* functions downstream of *Chd1*, we predicted that only knockdown in perineurial glia would impair the rapid induction of PHP. We found that RNAi knockdown of *Cad74A* in motoneurons or muscle had no effect on acute PHP, as indicated by robust increases in quantal content following PhTX application, consistent with intact homeostatic compensation. In contrast, perineurial glial‐specific knockdown of *Cad74A* abolished the compensatory increase in quantal content (Figure  8e,f; Figure S17). Importantly, baseline EPSP amplitude and quantal content were unaffected across all RNAi conditions compared to *wild‐type* controls, indicating that the PHP deficit was not due to altered baseline synaptic transmission. These results demonstrate that *Cad74A* is specifically required in perineurial glia for the rapid induction of PHP, mirroring the glial‐specific requirement for *Chd1* in acute PHP (Figure  8e,f; Figure S17; see also Figure 3a–d). Together, these findings establish *Cad74A* as one of the downstream effectors of *Chd1* in regulating homeostatic synaptic plasticity.

Finally, we examined whether *Cad74A* genetically interacts with *Chd1* in the rapid induction of PHP (Figure 8g,h; Figure S18). Both *Chd1^5^/+* and *Cad74A/+* heterozygous mutants exhibited a significant increase in quantal content, and EPSP amplitudes recovered to baseline levels in the presence of PhTX. In contrast, *Chd1^5^/+,Cad74A/+* trans‐heterozygous mutants displayed a significant reduction in EPSP amplitude and no change in quantal content after PhTX application, indicating that the rapid induction of PHP is completely abolished in the trans‐heterozygous mutant (Figure 8g,h; Figure S18). Importantly, both the *Chd1^5^/+* and *Cad74A/+* single heterozygous mutants and the *Chd1^5^/+,Cad74A/+* trans‐heterozygous mutants showed normal EPSP amplitudes and presynaptic release at baseline, demonstrating that the PHP impairment is not due to defects in basal synaptic transmission (Figure S18). Together, these findings show a strong genetic interaction between *Cad74A* and *Chd1*, supporting the conclusion that *Cad74A* functions downstream of *Chd1* to regulate homeostatic plasticity.

### 2.11 *Cad74A* is Essential for Larval Motor Function

Finally, we investigated whether *Cad74A*, like *Chd1*, is required for the regulation of motor function in *Drosophila* larvae. To this end, we quantified multiple locomotor parameters, including peak and average crawling speed, stride frequency, angular speed, bend angle, rhythm index, and fractal dimension, in *wild‐type*, *GluRIIA*, *Cad74A*, and *Cad74A,GluRIIA* double mutants (Figure 8i,j; Figure S19). *GluRIIA* mutants exhibited motor behavior comparable to *wild‐type* controls, with no significant differences in peak speed, average speed, angular speed, bend angle, or fractal dimension (Figure 8i,j). In contrast, *Cad74A* homozygous mutants displayed marked impairments, including significantly reduced peak and average crawling speeds, angular speed, stride frequency, and rhythm index, along with increased bend angle and greater movement complexity, as reflected by elevated fractal dimension values (Figure 8i,j; Figure S19). These findings demonstrate that *Cad74A*, one of the downstream effectors of *Chd1*, is required for normal larval motor behavior. Notably, motor impairments in *Cad74A* mutants were more severe than those observed in *Chd1^4/5^* mutants, suggesting that loss of *Cad74A* produces more pronounced functional deficits. This discrepancy may result from a more complete depletion of *Cad74A* in the mutant background or indicate that *Cad74A* also contributes to motor circuit regulation through *Chd1*‐independent mechanisms.

Although no additional reduction in peak or average crawling speed was observed in *Cad74A,GluRIIA* double mutants, likely due to a ceiling effect resulting from the already severe impairment in *Cad74A* single mutants, these double mutants exhibited significantly greater deficits in average bend angle and fractal dimension compared to *Cad74A* mutants alone (Figure 8i,j). These changes suggest more pronounced disruption of motor coordination and may reflect increased involuntary muscle contractions or aberrant neuromuscular activity. This phenotype closely mirrors that observed in *Chd1^4/5^,GluRIIA* double mutants (Figure 4d,f), underscoring that *Chd1* and *Cad74A* function within a shared or convergent signaling pathway that regulates motor output. Importantly, these findings demonstrate that disruption of homeostatic plasticity at the NMJ not only compromises synaptic stability but also exacerbates motor dysfunction and movement complexity. Taken together, our results suggest that *Cad74A* is one of the key downstream effectors of *Chd1* and is essential for both synaptic homeostasis and coordinated motor behavior. Moreover, they reveal a mechanistic link between impaired synaptic plasticity and complex motor phenotypes, highlighting the critical role of *Chd1*‐dependent signaling in maintaining functional motor output in *Drosophila* larvae.

## 3 Discussion

In summary, we demonstrate that *Chd1*, a chromatin remodeler linked to ASD, epilepsy, and intellectual disability, is essential for both the rapid induction and long‐term maintenance of PHP in *Drosophila* (Figure 1). Systematic analysis of *Chd1* expression in the larval nervous system revealed broad expression across multiple cell types (Figure 2). Importantly, *Chd1* operates in a cell type‐ and phase‐specific manner: it is required in perineurial glia for the acute induction of PHP, and in motoneurons, muscle, and glia for its sustained maintenance (Figure 3), underscoring the importance of intercellular communication in synaptic homeostasis. We further demonstrate that *Chd1* is dispensable for gross NMJ synapse morphogenesis but plays critical roles in regulating seizure susceptibility and motor function (Figures 1 and 4). Mechanistically, chronic PHP is impaired in *Chd1* mutants due to the failure to enhance presynaptic calcium influx, increase calcium channel abundance, and expand the readily releasable vesicle pool, core features of functional PHP (Figures 5 and 6). To uncover downstream effectors of *Chd1*, we conducted an electrophysiology‐based genetic screen guided by unsupervised machine learning. This screen identified *Cad74A*, a non‐classical cadherin, as one of the key glial‐specific effectors of *Chd1* required for the rapid induction of PHP (Figure 7). Like *Chd1*, *Cad74A* is essential for both acute and chronic forms of PHP and is necessary for proper motor behavior in larvae (Figure  8). Interestingly, both *Chd1^4/5^,GluRIIA* and *Cad74A,GluRIIA* double mutants, which lack chronic PHP, exhibit exacerbated motor impairments, highlighting the functional importance of PHP in preserving coordinated motor output (Figures 4 and 8). Together, our findings establish that *Chd1* acts in distinct cell types to regulate temporally separable phases of synaptic homeostasis and uncover core *Chd1*‐dependent molecular and cellular mechanisms. This work underscores the importance of glial epigenetic regulation and glia‐neuron signaling in maintaining synaptic stability. Our results also provide new insight into how disrupted compensatory regulation of presynaptic calcium influx and the RRP during chronic PHP may contribute to the pathogenesis of neurodevelopmental disorders.

### 3.1 Cell Type‐Specific Roles of *Chd1* in Regulating PHP

**FIGURE 9:**
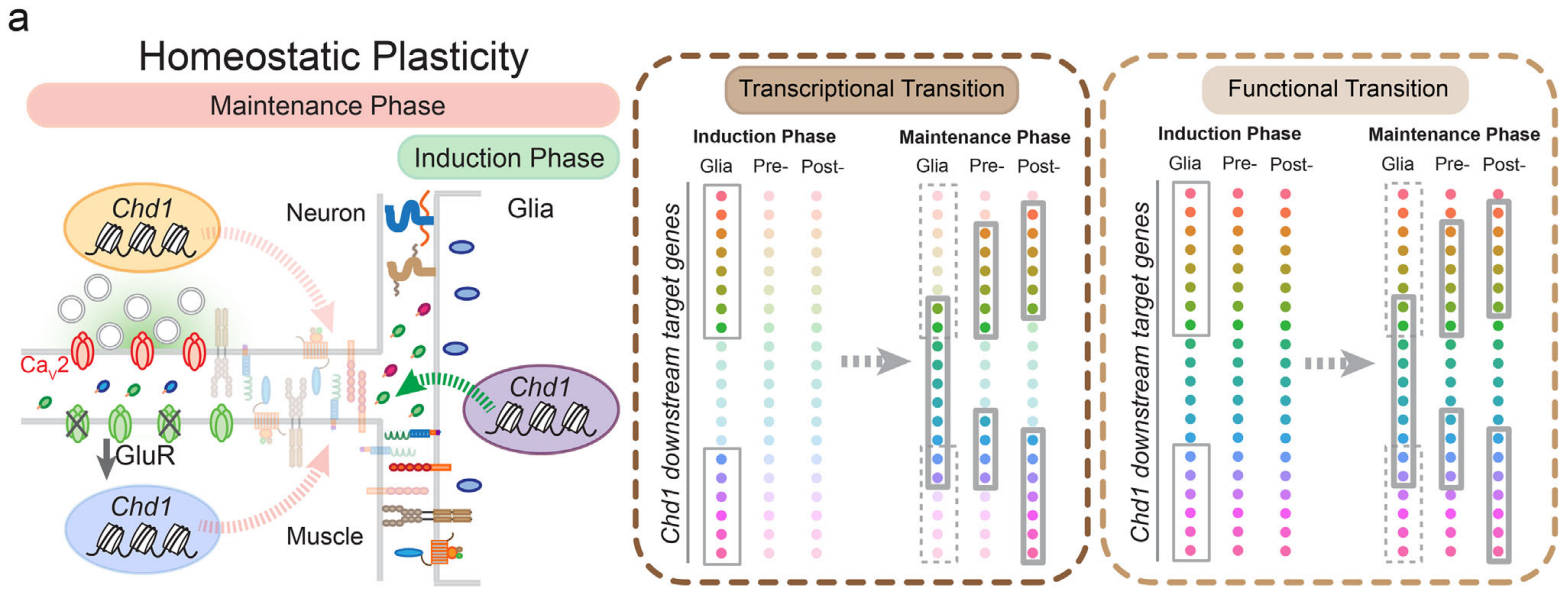
Model of cell type‐specific *Chd1* function in distinct temporal phases of PHP. (a) *Chd1* plays different roles in acute induction *v.s*. long‐term maintenance phase of PHP. *Chd1*‐dependent expression of signaling molecules in perineurial glia is required for the induction phase of PHP. During the consolidation and long‐term maintenance phases, *Chd1* activity in motoneurons and muscle also becomes necessary alongside glial contributions (left panel). One proposed mechanism is a “transcriptional transition,” in which *Chd1* regulates distinct target genes (indicated by colored dots in boxes) in each cell type (glia, presynaptic motoneuron, and postsynaptic muscle) depending on the temporal phase (middle panel). In this model, *Chd1*‐dependent expression in motoneurons and muscle is triggered by prolonged perturbations, such as deletion of the *GluRIIA* subunit. Alternatively, *Chd1* may regulate a common set of genes across all cell types, with the encoded proteins being selectively utilized in a phase‐ and cell type‐specific manner (“functional transition,” right panel). In this model, certain *Chd1*‐dependent signaling molecules (indicated by colored dots in boxes) act specifically in perineurial glia to mediate PHP induction, while motoneurons and muscle are recruited later to support the maintenance phase of PHP.

It is intriguing that *Chd1* is required in distinct cell types for the rapid induction (within minutes) and sustained maintenance (throughout the organism's lifespan) of PHP in *Drosophila* (Figure  9). One possible explanation for this cell type‐specific requirement is that *Chd1* regulates distinct effector gene programs in neurons, muscle, and glia. Notably, the rapid induction of PHP is known to occur independently of new transcription and translation [32], suggesting that *Chd1*‐dependent expression of glial effectors, such as *Cad74A*, must take place prior to PHP induction. Through our genetic screen, we identified 14 *Chd1*‐dependent effector genes required for acute PHP, including ECM components, cell adhesion molecules, a kinase, an adhesion GPCR, and an autophagy‐related gene (left panel, Figure 9; Table S1). Although the precise cellular sources and sites of action for these factors remain to be determined, it is likely that they are synthesized in glia and deposited at intercellular signaling sites in advance, thereby priming the synapse for rapid activity‐dependent modulation.

In contrast, long‐term perturbations, such as *GluRIIA* mutation, may trigger chronic, cell type‐specific transcriptional changes via *Chd1* in neurons, muscle, and glia, sustaining homeostatic signaling and enabling precise regulation of neurotransmitter release over time (a “transcriptional transition” from acute induction to long‐term maintenance phase of PHP, middle panel in Figure 9). An alternative, but not mutually exclusive, explanation is that *Chd1* regulates a common set of genes across cell types, but that distinct subsets of their encoded proteins are selectively utilized at different phases of PHP in a cell type‐specific manner (a “functional transition” from induction to maintenance phase of PHP, right panel in Figure 9). In this model, factors required immediately for induction are deployed from perineurial glia, while those necessary for maintenance become functionally active later across all three cell types. Together, these models emphasize that PHP is a highly dynamic and coordinated process, dependent on precise intercellular signaling. Acting as a master transcriptional regulator, *Chd1* orchestrates the spatial and temporal expression of key effector genes to support robust synaptic homeostasis across both acute and chronic timescales.

### 3.2 Cellular and Molecular Mechanisms Underlying *Chd1*‐Dependent Regulation of PHP

Using calcium imaging, super‐resolution microscopy, and electrophysiological approaches, we demonstrated that *Chd1* is required for regulating both presynaptic calcium influx and the RRP size during chronic PHP. Although our genetic screen primarily focused on identifying *Chd1* downstream targets required for the rapid induction of PHP, it is likely that a subset of these genes, including *Cad74A*, also contributes to the chronic phase (Figure 8c). Our data suggest that *Chd1* is required for the compensatory increase in presynaptic calcium influx and the RRP during chronic PHP. However, it remains unclear whether glial *Chd1* also regulates these processes during acute PHP, and which cell type(s) *Chd1* acts in to control presynaptic calcium influx and RRP during chronic PHP. A major open question is how *Chd1*‐dependent effectors coordinate the regulation of presynaptic calcium dynamics and vesicle release across distinct temporal phases of PHP.

In one scenario, because *Chd1* is specifically required in glia during the acute phase of PHP, its downstream effectors may modulate presynaptic calcium influx and/or RRP size through glia‐neuron signaling. Supporting this idea, previous studies have shown that *Chd1* genetically interacts with RIM, a presynaptic scaffolding protein and key PHP regulator involved in RRP enhancement [34, 72]. While *Chd1* itself functions as a chromatin remodeler, its downstream effectors, expressed on the glial surface or secreted into the extracellular space, may directly interact with neuronal or synaptic proteins to regulate vesicle release. In contrast, during the long‐term maintenance phase of PHP, *Chd1* functions across motoneurons, muscle, and glia. In this context, *Chd1* may regulate distinct transcriptional programs within motoneurons that directly modulate presynaptic calcium influx and/or RRP size. A critical next step will be to identify which *Chd1* targets are necessary for chronic PHP, in which cell types they function, and how they mechanistically influence neurotransmitter release. Although these questions lie beyond the scope of this study, the candidate effectors and signaling pathways uncovered by our screen establish a foundation for dissecting the molecular mechanisms by which *Chd1* governs synaptic homeostasis across time.

Cadherins are well known for their roles in remodeling synaptic architecture and strength through calcium‐dependent interactions with scaffolding proteins, receptors, kinases, and phosphatases [110, 111, 112]. *Cad74A* is a non‐classical cadherin among the 17 cadherins in *Drosophila* [107, 108, 109]. It contains extracellular cadherin domains, a transmembrane domain, and an uncharacterized cytoplasmic domain [107, 109]. *Cad74A* is likely a critical component of the PHP signaling network, potentially serving as an anchor for membrane receptors or as part of intercellular signaling complexes that mediate glia‐neuron communication. In many contexts, identical cadherins expressed on opposing cells engage in *trans* interactions via homophilic binding, enabling cell recognition and activation of downstream signaling pathways [113, 114, 115]. Interestingly, prior work suggests that heterophilic interactions can also occur between cadherins from different families [116, 117]. In addition, cadherins can interact in *cis* with cadherins on the same cell surface [118].

Notably, *Cad74A* expressed in perineurial glia is specifically required for the rapid induction of PHP (Figure 8e,f). Given that *Cad74A* expression in motoneurons or muscle is not required for acute PHP, an important question is which binding partner(s) interact with perineurial glial *Cad74A* to mediate signal transduction during acute PHP. For example, does glial *Cad74A* engage cadherins within the same family through homophilic interactions, or does it instead signal via heterophilic interactions with other cadherins expressed in adjacent cells? More broadly, it remains an open question whether *Cad74A* functions through homophilic or heterophilic interactions during distinct phases of PHP, and which downstream signaling pathways are activated by *Cad74A* to upregulate presynaptic neurotransmitter release during PHP. Mapping the spatial and temporal expression and function of *Cad74A* will provide important mechanistic insight into how distinct cell types coordinate across time to sustain synaptic homeostasis.

### 3.3 Glial Epigenetic Signaling Network in Synaptic Stabilization

Epigenetic regulation of gene expression plays a vital role in shaping synaptic and circuit function throughout brain development, normal behavior, and aging. However, the contribution of glial epigenetic signaling to synaptic and circuit regulation has been largely understudied. In *Drosophila*, three types of peripheral glia, wrapping glia, subperineurial glia, and perineurial glia, are localized along the peripheral nerves and at the NMJ [119, 120, 121]. Subperineurial glia lie beneath the perineurial glia, and together these two layers form the *Drosophila* equivalent of the blood–brain barrier [87, 121, 122]. Both subperineurial and perineurial glia extend along the peripheral nerves and interact directly with both presynaptic motoneurons and postsynaptic muscles. Our previous work demonstrated that epigenetic signaling in perineurial glia, mediated by the SAGA histone acetyltransferase complex, is essential for the secretion of the extracellular matrix protein *Drosophila Multiplexin* (*Dmp*) [41]. At baseline, Dmp is proteolytically cleaved to release the functional Endostatin domain, which enhances presynaptic calcium influx and neurotransmitter release during both the induction and maintenance of PHP [95]. These findings highlight the critical role of perineurial glia in mediating glia‐neuron communication to support synaptic homeostasis. Notably, while glial overexpression of a SAGA component rescued PHP in SAGA mutants, overexpression of Endostatin alone failed to do so, suggesting that SAGA regulates additional, yet unidentified, factors necessary for PHP [41]. This underscores the complexity of epigenetic regulation in PHP, and we speculate that multiple downstream effectors act in concert to finely tune neurotransmitter release. Reintroducing a single factor is insufficient to restore normal PHP.

While Chd1 is a component of the SAGA complex in yeast, its association with SAGA in *Drosophila* or mammals and the identity of downstream effectors relevant to synaptic stability remain unclear [123]. Here, we provide evidence that *Chd1* does not show consistent genetic interactions with SAGA components, and that overexpression of *Ada2b* fails to rescue PHP deficits in *Chd1^4/5^* mutants. These findings suggest that *Chd1* likely functions in parallel to the SAGA complex in regulating PHP. Nonetheless, given the large number of SAGA subunits, we cannot exclude the possibility that Chd1 interacts with specific components or converges on partially overlapping downstream pathways with SAGA.

Through a genetic screen, we identified *Chd1*‐dependent effector genes that span a range of functional domains. These findings not only allow us to dissect the role of *Chd1* in glia‐neuron interactions and PHP but also provide a framework for investigating how distinct epigenetic regulators and signaling pathways converge at the synapse to maintain physiological stability. Although mapping *Drosophila* perineurial glia to specific mammalian glial subtypes remains challenging due to evolutionary divergence in lineage and molecular identity, our recent computational analyses offer valuable insight. Cross‐species enrichment and single‐cell expression correlation analyses revealed that *Drosophila* perineurial glia share transcriptional profiles of “synaptic” genes, defined by GO terms containing “synaptic,” with human astrocytes and oligodendrocytes [39, 77, 124]. This suggests that *Drosophila* perineurial glia may employ conserved mechanisms for regulating synaptic function, similar to those used by glia in the human central nervous system. Nonetheless, it remains an open question whether mammalian glial cells, such as astrocytes, microglia, and oligodendrocytes, can directly detect changes in excitatory or inhibitory synaptic efficacy and contribute to synaptic stabilization through glia‐neuron interactions. Unraveling the role of glial epigenetic regulation and glia‐derived signaling in maintaining synaptic and circuit integrity presents an exciting and largely unexplored frontier in neuroscience research.

### 3.4 Epigenetic Control of Homeostatic Plasticity and Glial Function in ASD

There is growing evidence underscoring the importance of several interrelated features observed in ASD patients and animal models, including altered epigenetic regulation, frequent comorbidities with epilepsy, intellectual disability, and developmental delay, synaptic plasticity deficits, and glia‐specific roles of ASD‐linked genes in disease pathogenesis [5, 6, 7, 9, 55, 125, 126, 127]. Investigating the intersection of glial function, epigenetic regulation, and homeostatic synaptic plasticity may provide critical insights into how these diverse aspects of ASD pathology are mechanistically connected. Using *Drosophila* behavioral assays, we demonstrate for the first time that *Chd1* mutants exhibit increased seizure susceptibility and impaired motor function. Notably, *Cad74A*, a downstream effector of *Chd1*, is also required for normal motor behavior, mirroring the function of *Chd1*. When chronic PHP is disrupted, motor deficits are further exacerbated in *Chd1^4/5^,GluRIIA* and *Cad74A,GluRIIA* double mutants compared to *Chd1^4/5^* and *Cad74A* single mutants. These findings highlight a crucial role for synaptic homeostatic plasticity in maintaining motor behavior. Our previous work showed that glial expression of the ECM molecule *Dmp* increases during chronic PHP, not only at the NMJ but also along peripheral nerves and in the VNC, suggesting that PHP may involve circuit‐level modifications [41]. Given that *Chd1* is broadly expressed across multiple cell types in *Drosophila*, it is an intriguing open question whether *Chd1*‐mediated signaling contributes more broadly to circuit regulation and behavioral control beyond the NMJ.

Transcriptomic studies in mice have shown that *CHD2* regulates the expression of numerous genes associated with ASD, epilepsy, and intellectual disability [52, 55]. This raises the possibility that synaptic homeostatic plasticity, and specifically the glial epigenetic regulation of homeostatic modulators, may represent a shared locus of dysfunction across these neurodevelopmental disorders. One approach to explore this hypothesis would be to conduct a partially biased genetic screen for PHP modulators, focusing on *CHD2*‐regulated genes previously associated with these conditions. Additionally, comparative analyses of downstream regulatory targets across multiple models of epigenetic regulators implicated in ASD may help identify common effectors involved in synaptic homeostatic plasticity. Elucidating the potential role of homeostatic plasticity as a convergent mechanism underlying ASD and related disorders could provide valuable insight into disease etiology and inform the development of targeted therapeutic interventions.

## 4 Methods

### 4.1 *Drosophila melanogaster* Strains and Husbandry

Unless otherwise noted, the *w^1118^* strain was used as a *wild‐type* (*wt*) control. All *Drosophila* lines used were raised at 25°C, unless otherwise noted in figure legends, and maintained on standard molasses food. The following *Drosophila* stocks were used: *Chd1^4^* and *Chd1^5^* [59], *Df(2L)Exel7014* (BDSC BL7784), *eas^1^* [71], *GluRIIA^sp16^* [75], *Ada2b^1^* [41, 92], *Gcn5^Q186st^* (BL9334, [41]), *Gcn5^E333st^* (BL9333, [41]), *Cac^sfGFP^* [99], *Cad74A^f00312^* (BL18312), *UAS‐Redstinger.nls* (BL8546), *UAS‐GFP.nls* (BL4776), *UAS‐mCD8‐GFP* (BL5137), *UAS‐Ada2b‐3HA* (FlyORF F000122, [41]), *UAS‐Chd1‐RNAi* (KK101875, VDRC), *UAS‐Cad74A‐RNAi* (BL27485), *UAS‐Chd1* (generated for this study), *UAS‐Chd1‐3HA* (generated for this study), *UAS‐Chd1^KR^‐3HA* (generated for this study), *UAS‐Syt‐GCaMP8f* (generated for this study), *elav^C155^‐Gal4* (BL458), *OK371‐Gal4* (BL26160), *OK6‐Gal4* (BL64199, [79, 80]), *OK319‐Gal4* [80, 81], *D42‐Gal4* (BL8816, [80]), *Ib‐Gal4* (*dHb9‐Gal4*, BL83004, [80]), *Is‐Gal4* (*GMR27E09‐Gal4*, BL49227, [80]), *MHC‐Gal4* (BL84298), *Alrm‐Gal4* [78], *Mz709‐Gal4* [78], *Spg‐Gal4* [78], *NP6293‐Gal4* [41, 78], *Tubulin‐Gal4* (BL5138), *T2A‐Gal4* (*TI [CRIMIC.TG4.0]Chd1CR00688‐TG4.0*, BL78985, [82]). All genetic and RNAi mutants used in the genetic screen are listed in Table S1.

### 4.2 *Drosophila* Transgenic Lines and Antibody Generation

The DNA plasmids for *UAS‐Chd1*, *UAS‐Chd1‐3HA*, or *UAS‐Chd1^KR^‐3HA* were generated using the pENTR Directional TOPO Cloning Kit (K240020, Invitrogen). The *Chd1* coding sequence was amplified from *P [UAS‐Chd1+]126*  [59] using the following PCR primers: Forward CACCATGAGCCAGGCACTCAATGAATCGG and Reverse GGTCTGCGTGCGCCGCTC. To generate the *Chd1* ATPase mutant (K599R), the following primers were used for site‐directed mutagenesis in the pENTR vector using the Q5 Site‐Directed Mutagenesis Kit (E0554S, NEB): Forward GGCCTCGGCAGGACCATCCAA and Reverse CATCTCGTCGGCCAGGATC. For generation of the final constructs, the pENTR vectors were recombined with the destination vectors pUASg‐attB (1422, DGRC) or pUASg‐HA‐attB (1423, DGRC) to generate the non‐tagged or HA‐tagged Chd1, respectively, using LR Clonase II enzyme (11791020, Invitrogen). DNA constructs were purified using the GeneJET Plasmid Miniprep Kit (K0502, Invitrogen). After sequencing validation, constructs were sent to BestGene Inc. (Chino Hills, CA) for embryo injection. The ZH‐86Fb (III) strain was used as the targeted insertion site. The resulting *UAS‐Chd1*, *UAS‐Chd1‐3HA*, *UAS‐Chd1^KR^‐3HA* transgenic line was backcrossed into the *w^1118^* background. To generate *UAS‐Syt‐GCaMP8f*, we modified a previously engineered *UAS‐Syt‐GCaMP6s* plasmid [128] by replacing the *GCaMP6s* coding sequence with *GCaMP8f* (Addgene plasmid #162379), as described in [96, 97]. This construct was injected into the VK27 insertion site by BestGene Inc. (Chino Hills, CA). For custom antibody generation, a peptide corresponding to the C‐terminal amino acid sequence of Chd1 (residues 1867–1883: DYPADYRRSDYERRTQT) was synthesized and used to generate a polyclonal antibody (ThermoFisher Scientific). Antibody production followed a standard 90‐day immunization protocol with routine peptide injection.

### 4.3 Adult *Drosophila* Behavior Analysis

For the bang sensitivity assay, 8–10 adult flies (7–9 days post‐eclosion) were placed in an empty vial and vortexed at maximum speed for 10 s using a benchtop vortex mixer [66, 67]. The number of flies exhibiting seizure‐like activity at the bottom of the tube was quantified 10 s after stimulation. For the heat‐induced seizure assay, 4–5 adult flies (7–9 days post‐eclosion) were placed in an empty vial and submerged in a 42°C water bath for 2 min [68]. Seizure‐like behavior, characterized by loss of posture, convulsions, and uncoordinated movements, was recorded, and the number of flies exhibiting seizures at the bottom of the vial was quantified every 10 s. Seizure‐like behaviors were video recorded in both assays to capture motor abnormalities and convulsive movements indicative of seizure episodes, allowing assessment of seizure susceptibility and severity [129]. For climbing behavior (negative geotaxis) analysis, 8–10 adult flies (3–5 days post‐eclosion) were introduced into a vertical glass cylinder capped with parafilm perforated with small holes for ventilation. After a 5‐min habituation period, flies were gently tapped to the bottom of the cylinder and their climbing behavior was recorded for 1 min using an OKIOCAM T camera (OKIOLABS, [69]). The percentage of flies that crossed 10 cm, 15 cm, and 20 cm marker lines was quantified. Both male and female flies were included in all behavioral experiments.

### 4.4 *Drosophila* Larval Crawling Behavior Analysis

For larval crawling experiments, third instar *Drosophila* larvae of the appropriate genotypes were rinsed with PBS to remove residual food and yeast, then allowed to habituate for 2 min at room temperature. Each larva was gently placed at the center of a 10 cm Petri dish (pre‐warmed to room temperature) containing agar dyed with black food coloring. Free movement was recorded for 2 min using OKIOCAM T cameras (OKIOLABS) and OBS Studio software (28.0.0) at a resolution of 1920 × 1080 pixels and a fixed frame rate of 30 frames per second. A custom Python script was used to automatically track the larval centroid and body midline, extracting the movement path as a two‐dimensional time series. Both male and female larvae were included in the analysis.

Average speed was calculated as the mean speed across the entire crawling session. Instantaneous speed at each time point was computed using the distance between positions three frames before and three frames after the current frame (spanning a 0.2‐s window). Stride frequency (*F*_stride_) was estimated by identifying peaks in the power spectral density (PSD) of the instantaneous speed time series, calculated via the squared magnitude of the discrete Fourier transform (DFT) using a periodogram. Peak speed was determined by detecting local maxima in the instantaneous speed time series using a window of 15/*F*_stride_, and reported as the average of these peaks. Bend angle was computed as the maximum angle formed between the head, a midline point, and the tail in each frame. Angular speed was calculated as the change in bend angle per second, using the angle three frames before and three frames after each time point. Rhythm index was defined as the ratio of the total power of the top three peaks in the PSD to the total spectral power, with higher values reflecting more regular and rhythmic crawling. Movement complexity was estimated using the fractal dimension, analyzed as previously described [93].

### 4.5 Electrophysiology

Sharp‐electrode recordings were performed from muscle 6 in abdominal segments 2 and 3 of male and female third instar larvae using an Axoclamp 900A amplifier (Molecular Devices), as previously described [32, 130]. Recordings were conducted in HL3 saline, composed of (in mm): 70 NaCl, 5 KCl, 10 MgCl_2_, 10 NaHCO_3_, 115 Sucrose, 5 Trehalose, 5 HEPES, and 0.3 CaCl_2_. This solution was used for all current‐clamp experiments reported. EPSPs and mEPSPs were analyzed using StimFit (0.15.8) and MiniAnalysis (6.0.3, Synaptosoft). For the rapid induction of PHP, larvae were treated with 20 µm PhTX in an unstretched, partially dissected preparation for 10 min (PhTX: 276684‐27‐6, Santa Cruz Biotechnology or AOB0876, AOBIOUS). For each NMJ, the average EPSP amplitude was calculated from the mean peak response to 20–30 individual stimuli. mEPSPs were recorded continuously for 60–90 s. Quantal content was estimated for each NMJ as the ratio of the mean EPSP amplitude to the mean mEPSP amplitude. The mean quantal content across all NMJs for a given genotype is reported. Two‐electrode voltage clamp (TEVC) recordings were performed as previously described [104]. Following dissection in Ca^2+^‐free HL3, larval preparations were transferred to HL3 saline containing 1 mm Ca^2+^ prior to electrophysiological recording (Figure S13). Muscle cells were voltage‐clamped at −65 mV in a two‐electrode voltage configuration using an Axoclamp 900A amplifier (Molecular Devices). Apparent quantal content was estimated for each NMJ as the ratio of the mean EPSC amplitude to the mean mEPSP amplitude.

### 4.6 RRP Measurement

TEVC recordings were performed as previously described [104]. The RRP size was estimated using the method of cumulative EPSC amplitude analysis [34, 102, 103]. Following dissection in Ca^2+^‐free HL3, larval preparations were transferred to HL3 saline containing 1.5 mm Ca^2+^ prior to electrophysiological recording. Muscle cells were voltage‐clamped at −65 mV in a two‐electrode voltage configuration using an Axoclamp 900A amplifier (Molecular Devices), and EPSC amplitudes were recorded during a 60 Hz stimulus train. To estimate the RRP size, cumulative EPSC amplitudes were calculated for each stimulus train, and a linear fit was applied to the final 10 stimuli representing the steady‐state phase of depression. This linear fit was back‐extrapolated to time 0 to estimate the total synaptic output from the RRP. The apparent RRP size was then calculated by dividing the extrapolated cumulative EPSC amplitude at time 0 by the mean mEPSP amplitude recorded in the same cell under current‐clamp configuration prior to insertion of the second electrode.

### 4.7 Immunohistochemistry

Standard immunohistochemistry was performed as previously described [42, 95]. Briefly, dissected third instar larvae were fixed in 4% paraformaldehyde (PFA) in PBS at room temperature for 20 min, blocked in 5% normal goat serum (NGS) in PBST (PBS containing 0.1% Triton X‐100) for 2 h, and then incubated overnight at 4°C with primary antibodies diluted in PBST, following six brief washes. After primary antibody incubation, preparations were washed again and incubated with secondary antibodies diluted in PBST for 1.5 h at room temperature, followed by mounting in Vectashield without DAPI (H‐1000‐10, Vector Laboratories). For anti‐Syt1 immunolabeling, larvae were fixed in Bouin's fixative (HT10132‐1L, Sigma) at room temperature for 5 min. For Cac‐GFP immunolabeling, a modified protocol was used in which preparations were fixed in ice‐cold 100% ethanol for 5 min and mounted in ProLong Gold (P36930, Invitrogen). Primary antibodies included: mouse anti‐Bruchpilot (Brp, 1:100, Nc82, DSHB), rabbit anti‐Discs large (Dlg, 1:1000 [106]), rabbit anti‐GFP (1:1000, G10362, Invitrogen), mouse anti‐GFP (1:1000, A‐11120, Invitrogen), chicken anti‐GFP (GFP‐1010, Aves Labs), rabbit anti‐Syt1 (1:4000 [131]), and rabbit anti‐Chd1 (1:500, generated for this study). For confocal and dSTORM imaging, Alexa Fluor‐conjugated secondary antibodies (Alexa 488, Cy3, and Cy5, Invitrogen) were used at 1:300, along with Alexa Fluor 647‐conjugated anti‐HRP (1:100, 123‐605‐021, Jackson ImmunoResearch Laboratories). For STED imaging, the secondary antibodies used were anti‐mouse Alexa Fluor 594 (1:300, R37121, Invitrogen) and anti‐rabbit ATTO 647N (1:300, 611‐156‐122, Rockland Antibodies and Assays).

### 4.8 Dual In Situ Hybridization and Immunohistochemistry

The manufacturer's protocol was slightly modified for dual in situ hybridization and immunohistochemistry on whole‐mount *Drosophila* third instar larval brains and peripheral nerves using the RNAscope Multiplex Fluorescent V2 Assay (323100, ACDBio). A total of 15 brains with attached peripheral nerves were dissected in Ca^2+^‐free HL3 solution. After a rinse in PBS, tissues were fixed in 4% PFA at room temperature for 1 h. Samples were then rinsed three times for 5 min each in PBS with 0.1% Tween‐20 (PBS‐Tween) and subsequently dehydrated through sequential 10‐min incubations in 25%, 50%, 75%, and 100% methanol in PBS‐Tween. This was followed by incubation in 0.2 m HCl in 100% methanol for 30 min, and rehydration through 75%, 50%, and 25% methanol solutions, then PBS‐Tween, for 10 min at each step. For pretreatment, samples were transferred to 100 µm cell strainers submerged in 100°C Target Retrieval Solution (322000, ACDBio) for 2 min, then returned to PBS‐Tween. PBS‐Tween was subsequently replaced with 100% methanol for 1 min, then returned to PBS‐Tween. Samples were then treated with 300 µL of 2.5% Protease Plus (diluted in ddH_2_O) and incubated at 40°C for 5 min. Following three washes in PBS‐Tween (5 min each), samples were incubated overnight at 40°C with 2 drops of the Dm‐Chd1 probe (856271, ACDBio, generated for this study). Signal amplification steps were performed according to the manufacturer's protocol. Opal 570 (FP1488001KT, Akoya Biosciences) was used at a 1:1000 dilution in TSA buffer for 30 min at 40°C. Following in situ hybridization, standard immunohistochemistry was performed, beginning with the blocking step prior to primary antibody incubation. Samples were mounted in ProLong Gold Antifade Mountant (P36930, Invitrogen) for imaging.

### 4.9 Confocal Imaging

Confocal imaging of the VNC, peripheral nerves, and NMJ was performed using a laser scanning confocal microscope (LSM 880, Carl Zeiss). For Chd1 antibody validation and Chd1‐RNAi knockdown verification, Z‐stacks of the VNC and peripheral nerves were acquired using a 40× oil immersion objective (Plan‐Apochromat 40×/1.40 Oil DIC M27) at a resolution of 1200 × 1200 pixels. Maximum intensity projections were used for representative images of peripheral nerves, while medial and dorsal sections from the Z‐stacks were used for representative images of the VNC. For Chd1 cell type‐specific expression validation, Z‐stacks of the VNC, peripheral nerves, and segment A2 muscle 6/7 were captured using either a 20× objective (Plan‐Apochromat 20×/0.8) for VNC and muscle or a 40× oil immersion objective (Plan‐Apochromat 40×/1.40 Oil DIC M27) for peripheral nerves. Maximum intensity projections were used for representative images of muscles and peripheral nerves, while medial optical sections were selected for representative VNC images. For synapse morphology analysis, Z‐stacks were acquired using a 63× oil immersion objective (Plan‐Apochromat 63×/1.40 Oil DIC M27) at a resolution of 1200 × 1200 pixels. Images were taken from the NMJs on muscles 6/7 of segments A2 and A3, and maximum intensity projections were used for subsequent analysis in Fiji (NIH). Bouton number was quantified using Dlg immunolabeling, and active zone number was assessed using Brp staining, with segmentation performed using the watershed plugin in Fiji.

### 4.10 dSTORM Imaging

Images of Chd1 expression in glial cells along peripheral nerves were acquired using the Abbelight SAFe 360 system (2D SMLM). Excitation light was modulated to achieve either HiLo or TIRF illumination using an Olympus UPLAPO100XOHR objective (1.5 NA). A set of galvanometric mirrors ensured uniform illumination across the TIRF field. Fluorescent emitters were collected simultaneously through both the epifluorescence and SAF paths using two Hamamatsu Fusion BT sCMOS cameras (Hamamatsu City, Japan). Frame rate and exposure settings were optimized empirically. Typically, exposure times ranged from 10 to 50 ms per frame, with 2000 to 10 000 frames collected per imaging series. Alexa Fluor 647 “blinking” behavior was induced using a GLOX buffer specifically optimized for dSTORM. Image reconstructions were generated using NEO software (Abbelight).

### 4.11 STED Imaging

STED images of single 1b boutons were collected from synapses innervating muscles 6/7 in segment A2 using a Nikon Eclipse Ti2‐E confocal microscope equipped with an Abberior STEDYCON unit and a Nikon Plan Apo 100× NA 1.45 Lambda Oil DIC N2 objective. For each synapse, images of three boutons were acquired. The Alexa Fluor 594 and ATTO 647N channels were imaged using 561 nm (excitation)/775 nm (STED) and 640 nm (excitation)/775 nm (STED) laser pairs, respectively. This imaging configuration provides a lateral resolution of approximately 50 nm in the X/Y plane, while maintaining confocal‐level resolution in the Z plane. Each Z‐stack consisted of four optical sections acquired at 0.5 µm step size to minimize photobleaching. All images were captured in 16‐bit format with a region of interest (ROI) of 8 µm × 8 µm. Maximum intensity projections were generated and used for quantitative analysis in Fiji (NIH).

### 4.12 Calcium Imaging

Calcium imaging was performed using a galvanometer‐resonant hybrid laser scanning confocal system (FV3000, Olympus), equipped with a dual‐channel photomultiplier tube and a hybrid scanner incorporating four laser lines (405/488/561/640 nm), integrated into an electrophysiology setup. Single action potential‐evoked, spatially averaged Ca^2+^ transients were recorded from 1b boutons innervating muscles 6/7 of abdominal segments A2 and A3 in 1mM extracellular Ca^2+^ at room temperature, and analyzed as previously described  [132]. The 488 nm excitation light was focused onto the preparation using a PLAN FLUOR 60× water immersion objective (NA 1.0). Line scans across individual boutons were performed at a frequency of 833 Hz. Fluorescence changes were quantified as ΔF/F = (F(t) − F_baseline_) / F_baseline_, where F(t) is the integrated pixel fluorescence intensity at time t (averaged across pixels with >50% of peak intensity), and F_baseline_ is the mean fluorescence intensity prior to stimulation. For each preparation, 1–2 synapses (comprising 4–12 boutons) were imaged. The average Ca^2^^+^ transient per bouton was calculated from 3–6 line scans. Imaging datasets were excluded from analysis if the resting fluorescence decreased by >15% (possibly due to sample drifting), or if F_baseline_ fell outside the range of 300–900 A.U. Data from experimental and control groups were collected side by side under matched conditions.

### 4.13 Quantitative RT‐PCR

The protocol was adapted from [41]. VNCs and peripheral nerves were dissected from third instar larvae in Ca^2+^‐free HL3, with each biological replicate consisting of five brains. Total RNA was extracted using the RNeasy Plus Micro Kit (74034, Qiagen) according to the manufacturer's protocol. RNA purity was assessed using a NanoDrop Spectrophotometer (ThermoFisher Scientific), with A260/A280 ratio 2.0 considered indicative of high purity. Each biological replicate was split into +RT and –RT controls and reverse transcribed into cDNA using the SuperScript III First‐Strand Synthesis SuperMix for RT‐PCR (11752‐050, Invitrogen), following the manufacturer's instructions. Quantitative RT‐PCR was performed in triplicate using Mic tubes (71‐107, Genesee Scientific) on a Mic qPCR Cycler (Bio Molecular Systems) or 96‐well plate (HSP9601, Bio‐Rad) on a CFX Opus 96 Real‐Time PCR System (Bio‐Rad). Reactions were prepared using TaqMan Universal PCR Master Mix (4304437, Invitrogen) and TaqMan probes specific to *Chd1* (Dm01842738_g1, 4351372, Invitrogen), *Cad74A* (Dm01793527_m1, 4448892, Invitrogen), and the housekeeping gene *RpL32* (Dm02151827_g1, 4453320, Invitrogen). Mic Relative Quantification Software (Bio Molecular Systems) or CFX Maestro Software (Bio‐Rad) was used to obtain threshold cycle (Ct) values, and fold changes in mRNA expression were calculated using the ΔΔCt method (2^−ΔΔCt^).

### 4.14 Protein Structure and scRNA‐seq Data Analysis

Protein structures for mouse CHD2 (AF‐E9PZM4‐F1) and *Drosophila* Chd1 (AF‐Q7KU24‐F1) were predicted by AlphaFold [61, 62]. Structural comparisons were performed using pairwise structural alignment via the jFATCAT method (RCSB Protein Data Bank). Single‐cell RNA‐seq (scRNA‐seq) data from the mouse cortex ([76], GSE60361) were clustered following the approach described in [133]. The *Drosophila* scRNA‐seq data for larval VNC were obtained from ([134], GSE235231). Cell clusters (31 040 cells) were presented in the original study. The *Drosophila* adult brain scRNA‐seq dataset was obtained from ([77], GSE107451). Cell clusters (57 000 cells) were visualized using scenic_tsne1 and scenic_tsne2 coordinates, as presented in the original study.

### 4.15 Gaussian Mixture Model for Genetic Screen Data Analysis

We applied a GMM, an unsupervised machine learning approach, to cluster electrophysiological data obtained under PhTX treatment in a two‐dimensional space defined by quantal content (QC) and EPSP amplitude. The probability density function for observing a data point (QC,  EPSP) was modeled as a weighted sum of multivariate Gaussian distributions:

$$P\;\left(\mathrm{QC},\mathrm{EPSP}\right)=\sum_{i=0}^{n}w_{i}\cdot N_{i}\left(\mathrm{QC},\;\mathrm{EPSP}\right)$$

where $N_{i}\left(\mathrm{QC},\mathrm{EPSP}\right)$ represents the two‐dimensional Gaussian distribution for cluster *i*, and *w_i_* is the corresponding weight. To infer the number of distinct states underlying homeostatic regulation, we tested models with *n* = 1, 2, 3, 4, and 5, and identified *n* = 3 as the optimal model based on the lowest AIC. Without loss of generality, we designated cluster 0 as containing the lowest EPSP values and cluster 2 as containing the highest. Intuitively, mutants located near the center of cluster 0 were hypothesized to impair PHP.

To formally quantify the likelihood that a given mutant belonged to the PHP‐deficient cluster, we computed the posterior probability *q*_0_, defined as the probability that a given (QC,  EPSP) point arises from cluster 0:

$$q_{0}=P\;\left(0\left|QC\right.,\;EPSP\right)=\frac{w_{0}\;N_{0}\left(QC,\;EPSP\right)}{\sum_{j=0}^{2}w_{j}\;N_{j}\left(QC,\;EPSP\right)}$$

We used *q*_0_ as a predictor of PHP impairment and validated this approach by performing baseline (−PhTX) recordings for the 28 mutants with *q*_0_ > 0.02 (i.e., very low probability of belonging to the high‐EPSP cluster) to ensure a high true positive rate and minimize the risk of overlooking true hits. A mutant was classified as blocking PHP (label = 1) if the difference in QC between +PhTX and −PhTX conditions was not statistically significant (*p* > 0.05); otherwise, it was labeled as 0. Using *q*_0_ as a predictive score, we constructed a ROC curve and observed an AUC of 0.88, indicating strong predictive performance in a biological context.

This approach differs from traditional supervised learning, where models are trained on labeled data. Here, we trained an unsupervised model on the +PhTX dataset without any labels, derived a probabilistic classifier from the model, and validated it using independent −PhTX recordings. This strategy demonstrates that our framework is both unsupervised and generalizable across experimental conditions. It enables efficient identification of candidate PHP‐deficient mutants while reducing the number of required baseline recordings.

### 4.16 Statistics

Data were analyzed using Stimfit (0.15.8), MiniAnalysis (6.0.3, Synaptosoft), Python (3.7.12), or Fiji (NIH). Both male and female animals were included in all experiments and analyses. An α‐level of 0.05 was used to determine statistical significance. Data are presented as mean ± standard error of the mean (SEM), with exact sample sizes indicated in the figure legends and Table S1. GO enrichment analysis was done using Python library GOATOOLS (1.2.3, [135]). Statistical analyses were performed using GraphPad Prism (10.4.1), and all figures were created using Adobe Illustrator (2023, Adobe). Normality of residuals from ANOVA tests was assessed using the D'Agostino‐Pearson omnibus test. For datasets with normally distributed residuals, one‐way ANOVA or Student's t‐tests were used. For datasets with non‐normal residuals, the nonparametric Kruskal‐Wallis test was applied. When comparing more than two conditions, Bonferroni post hoc tests were used following one‐way ANOVA, and Dunn's post hoc tests were used following Kruskal–Wallis. For quantification of seizure incidence during heat‐induced seizure assays, two‐way ANOVA with Bonferroni post hoc tests was performed to compare genotypes across the time series. All sample sizes and *p*‐values from statistical tests are reported in the figure legends and in Table S1.

## Author Contributions

D.T.M. data collection, data analysis, interpretation, and writing manuscript. T.C., Y.C. project design, data collection, data analysis, interpretation, writing, and editing manuscript. C.L., R.E.N., R.H., G.L.C., Y.X., S.W.A., J.W., and T.W. data collection and analysis. K.H., C.Q., and D.K.D. reagent generation, data collection, and analysis. P.M.P. reagent generation. G.W. data analysis. S.N., H.P., and S.V. project design. T.T.W. conceptualization, project design, data collection, data analysis, data interpretation, supervision, writing, and editing manuscript.

## Funding

Work in the laboratory of T.T.W. was supported by NIH Grants R01NS117372 (T.T.W.) and R01MH134978 (T.T.W.), National Science Foundation Career Award 2440057 (T.T.W.), Brain and Behavior Research Foundation Young Investigator Award 27792 (T.T.W.), Simons Foundation Autism Research Initiative (SFARI) BTI Award 551354 (T.T.W.), the Shenoy Undergraduate Research Fellowship in Neuroscience from the Simons Foundation AN‐SURFiN‐00008126 (T.T.W.), AN‐SURFiN‐00003291 (T.T.W.), and SFI‐AN‐SURFiN‐00008776 (T.T.W.), and NIH NRSA predoctoral fellowship F31NS139658 (Y.C.). Work in the laboratory of D.K.D. was supported by an NIH Grant R01NS126654 (D.K.D.).

## Conflicts of Interest

The authors declare no conflicts of interest.

## Supporting information




**Supporting File**: advs74782‐sup‐0001‐SuppMat.docx.

## Data Availability

All data generated are included in the main Figures and Supporting Information. The datasets that support the findings of this study are available from the corresponding author upon reasonable request. Custom Python codes are available at Github: RRP estimation: https://github.com/wanglab‐georgetown/HFRP_analysis; scRNA‐seq data cluster and visualization: https://github.com/wanglab‐georgetown/JOINT; Larval crawling fractal dimension analysis: https://github.com/wanglab‐georgetown/fractal. All transgenic lines and antibodies generated for this study are available upon request.
