# Supplementary material for: Temporal and Cell‐Specific Regulation of Synaptic Homeostasis by the Chromatin Remodeler Chd1
Source: Adv Sci (Weinh). 2026 Mar 15;13(33):e10538. doi: 10.1002/advs.202510538 (PMC13271595; doi:10.1002/advs.202510538)
Supplement: Supplementary file 1 — Supporting File: advs74782‐sup‐0001‐SuppMat.docx. [file ADVS-13-e10538-s001.docx]

**SUPPORTING INFORMATION**

**Temporal and Cell-Specific Regulation of Synaptic Homeostasis by the Chromatin Remodeler *Chd1***

*Danielle T. Morency^1,2^, Tao Cui^1^, Yimei Cai^1^, Chloe Lok^3^, Rachel E. Nokku^3^, Ruoxian Huang^4^, Grace L. Chu^3^, Yumeng Xie^1^, Saleem W. Abu-Tayeh^1^, Kaikai He^5^, Chengjie Qiu^5^, Junyi Wang^4^, Paxton M. Paganelli^1^, Ting Wang^1^, Gabrielle Williams^1^, Sreejith Nair^6^, Huadong Pei^6^, Dion K. Dickman^5^, Stefano Vicini^1,2^, Tingting Wang^1,2,*^*

**Address:**

^1^ Department of Pharmacology & Physiology, Georgetown University Medical Center, Washington, D.C. 20007, USA

^2^ Interdisciplinary Program in Neuroscience, Georgetown University Medical Center, Washington, D.C. 20007, USA

^3^ Biology Department, Georgetown University, Washington, D.C. 20007, USA

^4^ Department of Human Science, School of Health, Georgetown University, Washington, D.C. 20007, USA

^5^ Department of Neurobiology, University of Southern California, Los Angeles, CA 90089, USA

^6^ Department of Oncology, Georgetown Lombardi Comprehensive Cancer Center, Georgetown University Medical Center, Washington, D.C. 20007, USA

*Correspondence: [tw652@georgetown.edu](mailto:tw652@georgetown.edu)

**SUPPLEMENTAL METHODS**

**ChIP-PCR**

ChIP was performed using male adult heads. For heat shock controls, male flies were exposed to 35°C for 30 minutes prior to sample collection. Two hundred male adult heads were flash-frozen in liquid nitrogen. Samples were homogenized and crosslinked with 1.8% formaldehyde (F8775, Sigma) at room temperature for 10 minutes and quenched with a 1:10 volume of 2.625 M glycine (G8898, Sigma). Following centrifugation at 3,000g for 5 minutes at 4°C, the supernatant was discarded and samples were washed twice with cold PBS containing 0.5% BSA (A9647, Sigma).

Pellets were resuspended in nucleus isolation buffer (10 mM HEPES, pH 8.0, 85 mM KCl, 1 mM EDTA, 0.5% Igepal, protease inhibitors) and centrifuged at 500g for 5 minutes at 4°C. The pellets were then resuspended in nucleus lysis buffer (20 mM Tris-HCl pH 7.5, 150 mM NaCl, 1 mM EDTA, 0.5 mM EGTA, 0.4% Na-deoxycholate, 0.1% SDS, 1% Igepal, 0.5 mM DTT, protease inhibitors). All solutions contained protease inhibitor cocktail (A32961, Invitrogen). Samples were sonicated using a Bioruptor Pico sonication device (Diagenode; 30 s on/30 s off, 25 cycles). Sonicated samples were centrifuged at 16,000g for 5 minutes at 4°C. Input samples (1% of the supernatant) were reserved, and the remaining sonicated chromatin was used for each IP reaction.

Chromatin samples were added to rabbit anti-Chd1 antibody (generated for this study) -bound Protein A Dynabeads (REF10006D, Invitrogen) and incubated on a rotator overnight at 4°C. Samples were washed twice with wash buffer I (20 mM Tris-HCl pH 7.5, 150 mM NaCl, 1 mM EDTA, 0.5 mM EGTA, 0.4% Na-deoxycholate, 0.1% SDS, 1% Igepal, protease inhibitors), three times with wash buffer II (10 mM Tris-HCl pH 7.5, 250 mM LiCl, 1% Igepal, 1% Na-deoxycholate, 1 mM EDTA, protease inhibitors), once with TE buffer containing protease inhibitors (10 mM Tris-HCl pH 7.5, 1 mM EDTA, protease inhibitors), and once with TE buffer without protease inhibitors.

DNA was eluted with elution buffer (1% SDS, 10 mM Tris-HCl pH 8.0, 1 mM EDTA) for 20 minutes at 65°C on a Thermomixer (Model 5382, Eppendorf) at 1,200 rpm. A second elution with was performed for 10 minutes, and the two eluates were combined. All IP and input samples were treated with RNase A (12091021, Invitrogen) for 1 hour at 37°C and reverse crosslinked overnight at 65°C. Proteinase K (100005393, Invitrogen) was added, and samples were incubated at 50°C for 1 hour. DNA was purified using QIAquick DNA cleanup columns (28506, QIAGEN). DNA concentration was determined using a Nanodrop spectrophotometer.

ChIP DNA was quantified by SYBR Green real-time PCR using iTaq Universal SYBR Green Supermix (1725120, Bio-Rad). The following primers were used: HSP70-25(F): CGGAGAGTCAATTCTATTCAAACA and HSP70-25(R): CTTGCACTTTATTGCAGATTGT [1]; Cad74A-48(F): TGAGCGCCTCTCATTGGAAA and Cad74A-48(R): CTTACCTTCTGGCCGGACTT (the Cad74A primer pair starts 48 bp upstream of the transcription start site). ChIP DNA (or 1% input) and RNase/DNase-free H₂O were added to a final reaction volume of 20 μL.

Reactions were run in technical triplicate on a BIO-RAD CFX Opus 96 machine using the following program: 95°C for 2 minutes, followed by 50 cycles of 95°C for 5 seconds and 60°C for 30 seconds with fluorescence acquisition at the annealing/extension step. A melt-curve analysis was performed at the end of the run to confirm product specificity.

**Immunoprecipitation (IP)**

For each IP reaction, 150 fly heads (male and female) were used. Heads were homogenized using a Microtube Homogenizer (D1030, BeadBug) in NP-40 lysis buffer (6 mM Na₂HPO₄, 4 mM NaH₂PO₄, 1% NP-40, 150 mM NaCl, 2 mM EDTA, 50 mM NaF, 4 µg/mL leupeptin, 0.1 mM Na₃VO₄) supplemented with protease inhibitors (04693124001, Roche). After a 20-minute incubation on ice, samples were sonicated using a Branson SFX 550 Digital Sonifier for 10 seconds (23–26% amplitude, 1 s on/off cycle). Samples were then incubated on ice for 10 minutes and centrifuged at 15,000 rpm for 15 minutes at 4°C. The supernatant was collected for IP.

Protein G Dynabeads (REF10003D, Invitrogen) were washed three times in antibody binding buffer and then resuspended with 5 µg rat anti-HA antibodies (11867423001, Sigma) in antibody binding buffer. The bead–antibody mixture was incubated on a nutator for 2 hours at room temperature. After washing, the antibody-bound beads were added to the IP samples and rotated overnight at 4°C. The following day, beads were collected using a magnetic rack and washed three times with washing buffer. Bound proteins were eluted by boiling in sample buffer (NP0007, Invitrogen) and reducing reagent (NP0009, Invitrogen) for 10 minutes at 95°C.

**Western Blot**

Samples were run on NuPAGE Bis-Tris Mini Protein Gels, 4–12% (NP0321BOX, Invitrogen) and transferred to PVDF membranes (IB24001, Invitrogen) using the Invitrogen iBlot 2 system. Membranes were blocked with 1× Clear Milk (37587, Invitrogen) in PBS containing 0.1% Tween-20 (BP337-100, ThermoFisher Scientific), and then incubated overnight at 4°C with the appropriate primary antibodies diluted in the same buffer (rat anti-HA, 1:500, 11867423001, Sigma; rabbit anti-Chd1, 1:250, generated for this study). Following six washes with PBS with 0.1% Tween-20, membranes were incubated for 1.5 hours at room temperature with HRP-conjugated secondary antibodies (goat anti-rat, 31470, Invitrogen; goat anti-rabbit, 31460, Invitrogen) diluted 1:10,000 in blocking buffer. After another six washes, membranes were treated with SuperSignal West Pico PLUS Chemiluminescent Substrate (34580, Invitrogen) and imaged using the Amersham ImageQuant 600 Western blot imaging system (Cytiva).

**SUPPLEMENTAL FIGURES**

**
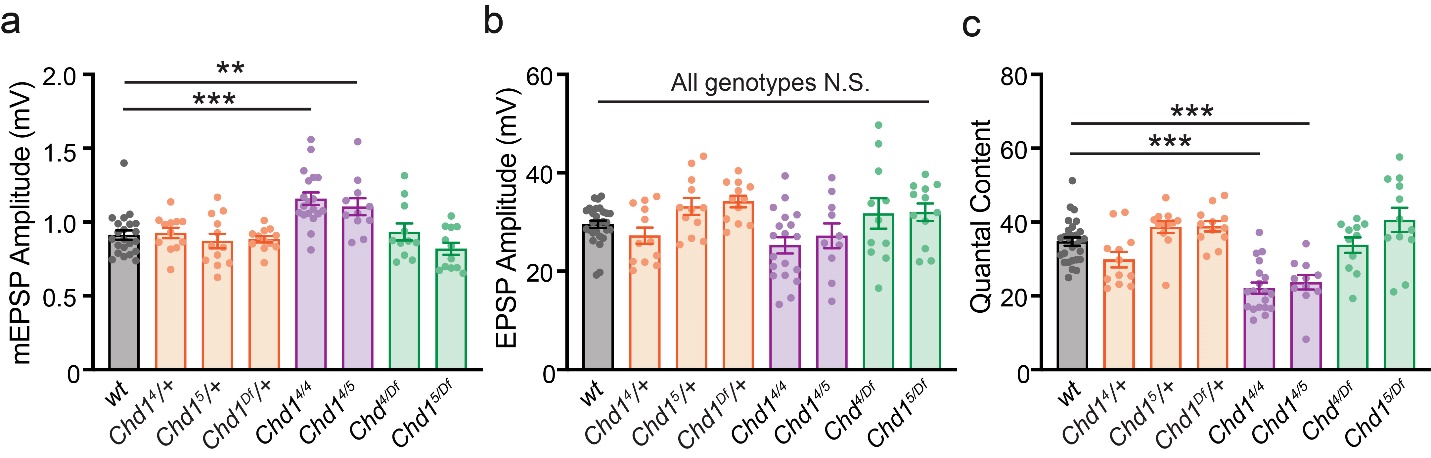
**

**Figure S1. Baseline Synaptic Transmission in *Chd1* mutants.**

**a-c.** Average mEPSP amplitude (**a**), EPSP amplitude (**b**), and presynaptic release (quantal content, **c**) at baseline. Genotypes and sample sizes: *wild-type* (*wt*, n = 22), *Chd1^4^/+* (n = 12), *Chd1^5^/+* (n = 12), *Chd1^Df^/+* (n = 12), *Chd1^4/4^* (n = 19), *Chd1^4/5^* (n = 11), *Chd1^4/Df^* (n = 11), *Chd1^5/Df^* (n = 12). Mean ± SEM; **p < 0.01, ***p < 0.001, N.S. not significant; one-way ANOVA with Bonferroni test for multiple comparisons.

**
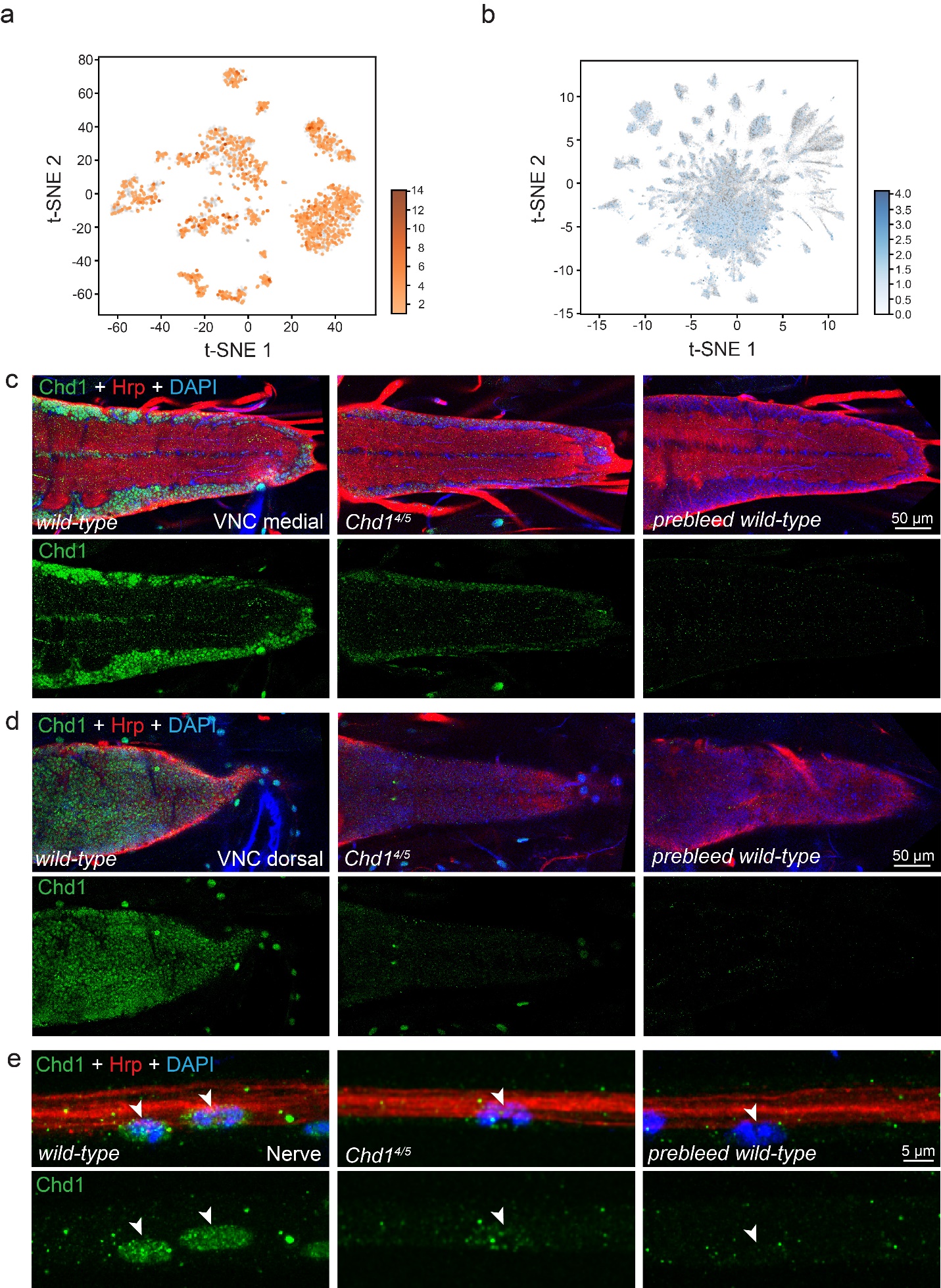
**

**Figure S2. Expression of Mouse *CHD2* and *Drosophila Chd1* and Validation of the Chd1 Antibody Specificity.**

**a-b.** Expression of *CHD2* in mouse cortical cells (**a**) and *Chd1* in the *Drosophila* adult brain (**b**) visualized by t-SNE plots. Expression levels are indicated in orange (mouse *CHD2* [2], **a**) and blue (*Drosophila Chd1* [3], **b**).

**c-e.** Representative confocal images of third instar larval medial (**c**) and dorsal (**d**) sections of the ventral nerve cord (VNC), and peripheral nerves (**e**), immunolabeled for Chd1 (green), neuronal membrane (HRP, red), and nuclei (DAPI, blue). Images are shown for *wild-type* (left panels), *Chd1^4/5^* mutants (middle panels), and *wild-type* labeled with pre-bleed serum as a negative control for Chd1 immunostaining (green, right panels). Peripheral glial nuclei along the peripheral nerves are indicated by arrowheads (**c**).

**
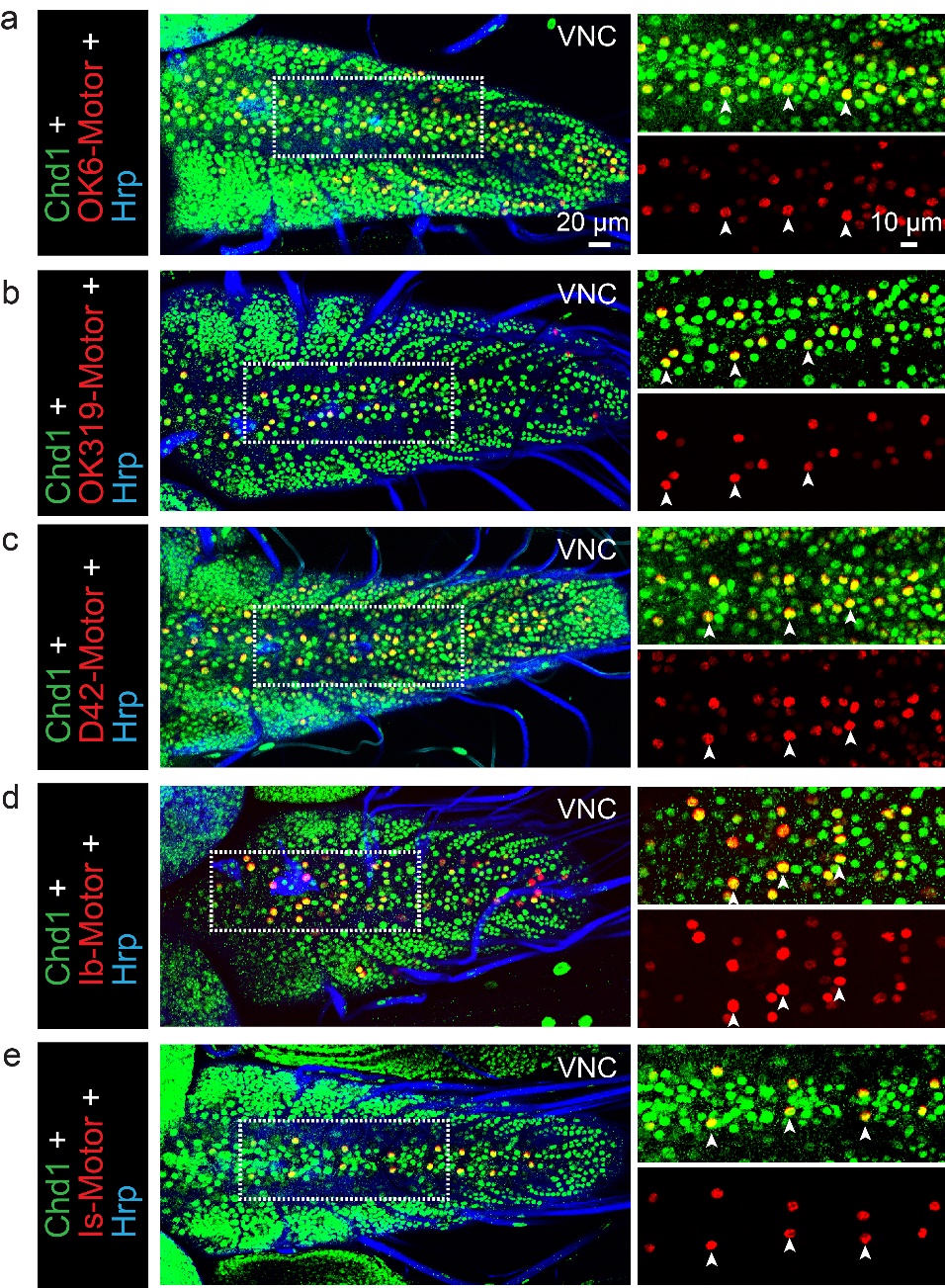
**

**Figure S3. Chd1 Is Expressed in Motoneurons of the Larval VNC.**

**a-e.** Confocal images of third-instar larval ventral nerve cords (VNCs) showing motoneurons labeled by motoneuron-specific *Gal4* drivers. *UAS-RedStinger.nls* marks motoneuron nuclei (red) driven by *OK6-Gal4* (**a**, OK6-Motor, *OK6-Gal4>UAS-RedStinger.nls*), *OK319-Gal4* (**b**, OK319-Motor, *OK319-Gal4>UAS-RedStinger.nls*), *D42-Gal4* (**c**, D42-Motor, *D42-Gal4>UAS-RedStinger.nls*), *Ib-Gal4* (**d**, Ib-Motor, *dHb9-Gal4>UAS-RedStinger.nls*), and *Is-Gal4* (**e**, Is-Motor, *GMR27E09-Gal4>UAS-RedStinger.nls*). Chd1 protein (green) and neuronal membranes (HRP, blue) are shown. Regions outlined by white boxes in the left panels are shown at higher magnification in the right panels. Chd1 is detected in motoneuron nuclei (arrowheads).


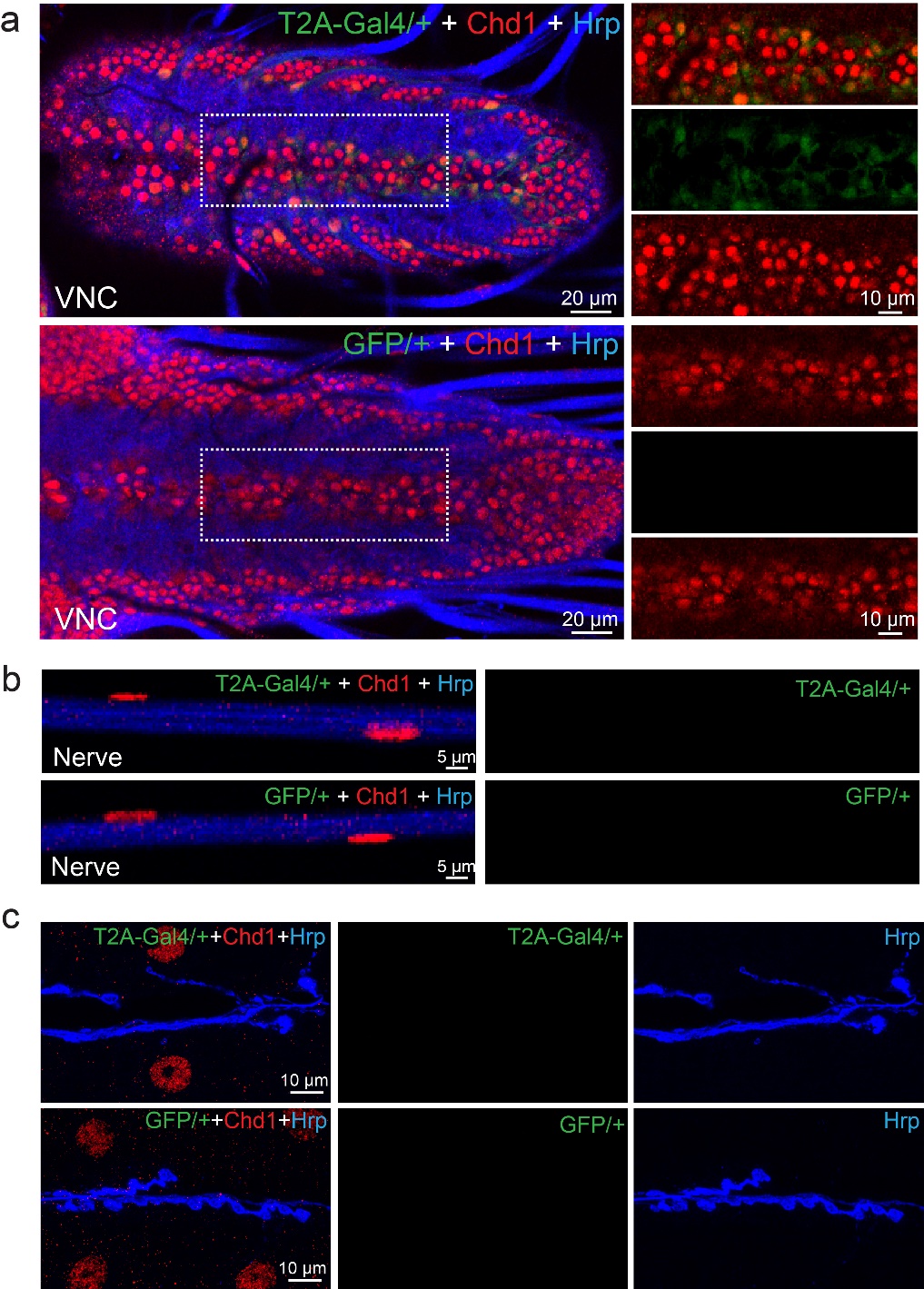


**Figure S4. *T2A-Gal4/+* and *UAS-CD8-GFP/+* Heterozygous Controls for Endogenous *Chd1* Expression.**

**a-c.** Confocal images of third-instar larvae showing the ventral nerve cord (VNC, **a**), peripheral glia along the nerve (**b**), and presynaptic boutons and muscle (**c**) in heterozygous *T2A-Gal4* (*T2A-Gal4/+*) and *UAS-CD8-GFP* (*UAS-CD8-GFP/+*) control animals. Regions outlined by white boxes in the left panels are shown at higher magnification in the right panels (**a**). Chd1 protein (red) and neuronal membranes (HRP, blue) are shown. GFP (green) is not detected in these heterozygous controls.


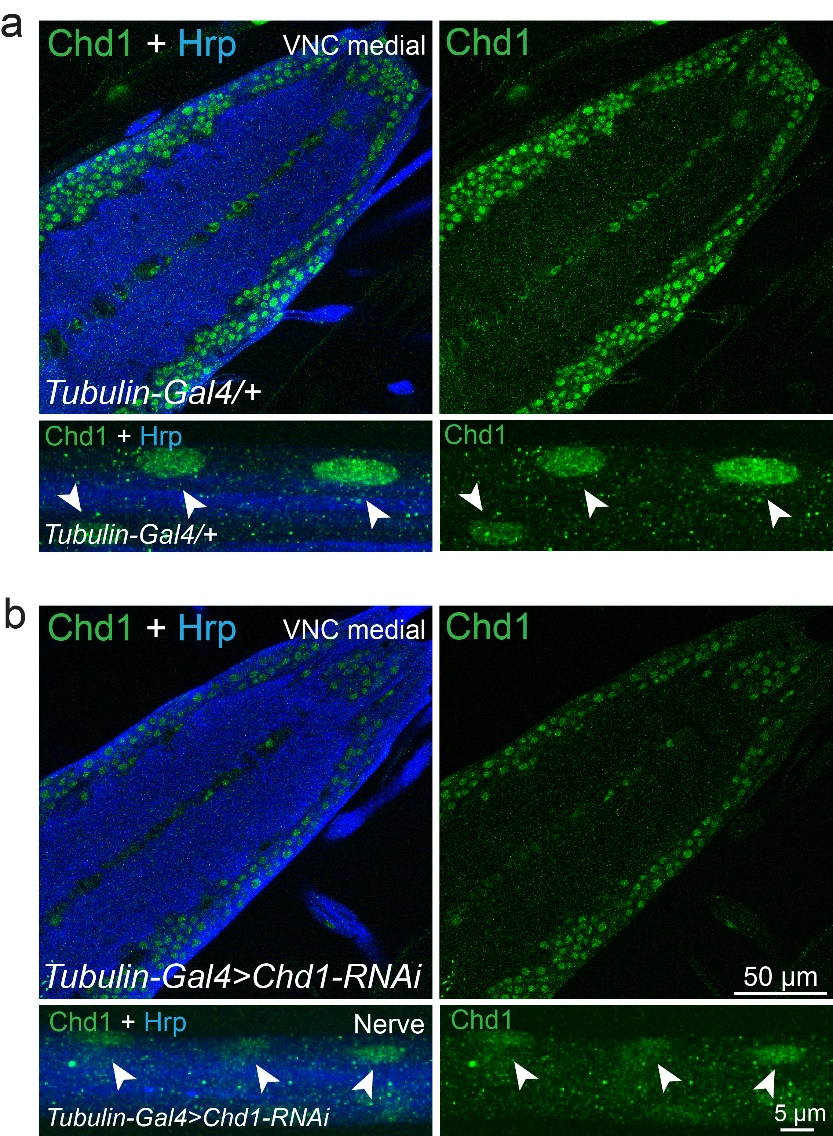


**Figure S5. Validation of *UAS-Chd1-RNAi* knockdown efficiency.**

**a-b.** Representative confocal images of third instar larval medial sections of the ventral nerve cord (VNC, upper panels) and peripheral nerves (lower panels), immunolabeled for Chd1 (green) and neuronal membrane (HRP, blue) in the control (*Tubulin-Gal4/+*, **a**) and ubiquitous *Chd1* knockdown (*Tubulin-Gal4>UAS-Chd1-RNAi*, **b**). Peripheral glial nuclei along the peripheral nerves are indicated by arrowheads (lower panels).

**
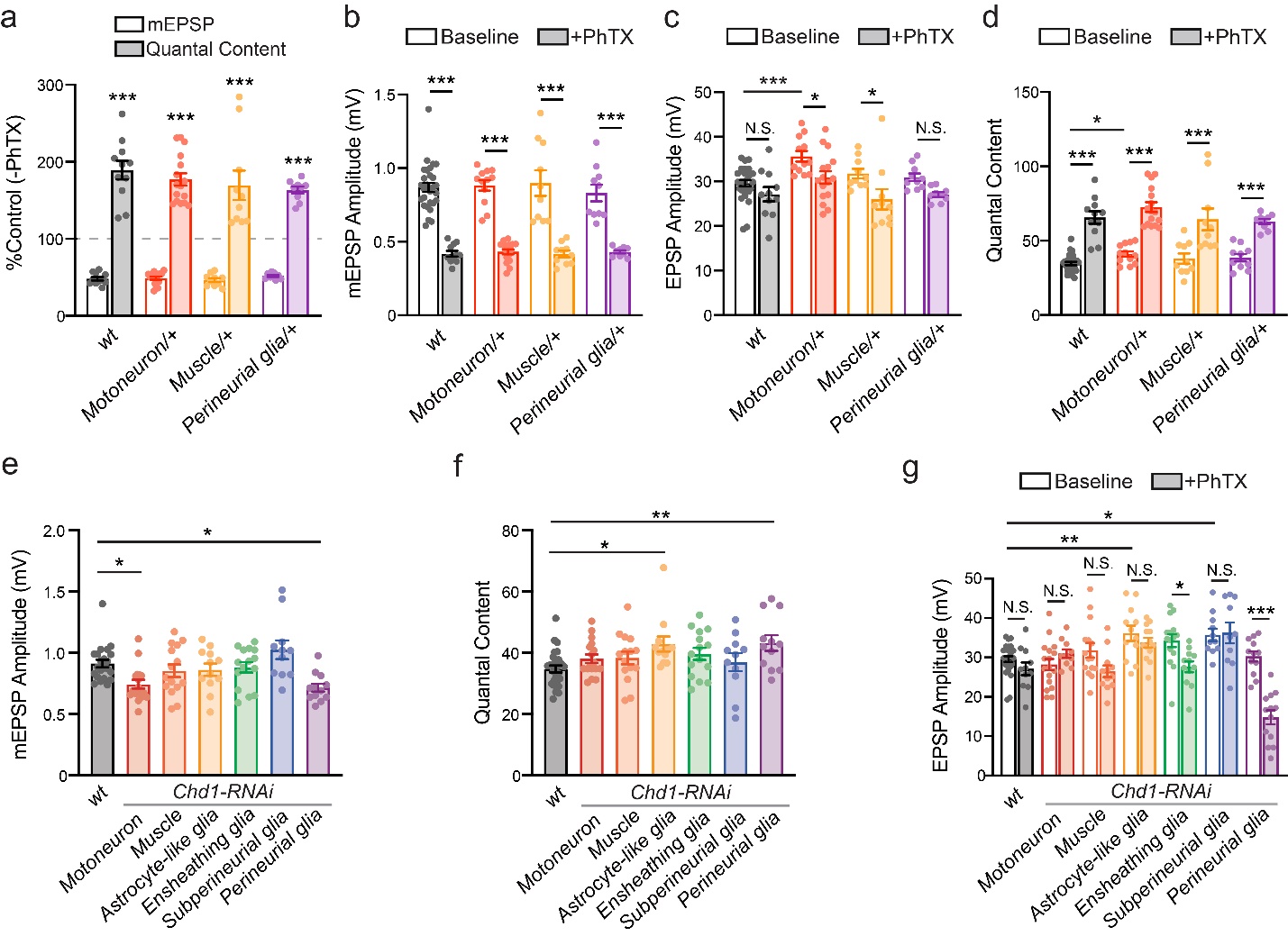
 Figure S6. Heterozygous *Gal4* Controls and Raw Data for Tissue-Specific Knockdown of *Chd1* in Acute PHP.**

**a.** Average mEPSP amplitude (open bars) and presynaptic release (quantal content, filled bars). Data for each genotype are presented as the percent change in the presence of PhTX (+PhTX) compared to the same genotype recorded in the absence of PhTX (baseline). Genotypes and sample sizes: *wild-type* (*wt*, n = 28, 11 for –PhTX and +PhTX, respectively), motoneuron *Gal4* control (*OK371-Gal4/+*, n = 12, 16), muscle *Gal4* control (*MHC-Gal4/+*, n = 10, 10), and perineurial glial *Gal4* control (*NP6293-Gal4/+*, n = 10, 9). Mean ± SEM; ***p < 0.001, N.S. not significant; one-way ANOVA with Bonferroni test for multiple comparisons. Non-normalized raw data were used for statistical analysis.

**b-d.** Non-normalized values corresponding to (**a**): average mEPSP amplitude (**b**), EPSP amplitude (**c**), and quantal content (**d**) in the absence (open bars) and presence (filled bars) of PhTX. Mean ± SEM; *p < 0.05, ***p < 0.001, N.S. not significant; one-way ANOVA with Bonferroni test for multiple comparisons.

**e-g.** Baseline synaptic transmission measurements for each tissue-specific *Chd1* knockdown. mEPSP amplitude (**e**) and quantal content (**f**) in the absence of PhTX. Non-normalized average EPSP amplitude (**g**) in the absence (baseline, open bars) and presence of PhTX (+PhTX, filled bars). Genotypes and sample sizes: *wild-type* (*wt*, n = 22, 11 for –PhTX and +PhTX, respectively); *Chd1* knockdown in motoneurons (*OK371-Gal4>UAS-Chd1-RNAi*, n = 17, 10), muscles (*MHC-Gal4>UAS-Chd1-RNAi*, n = 15, 12), astrocyte-like glia (*Alrm-Gal4>UAS-Chd1-RNAi*, n = 12, 13), ensheathing glia (*Mz709-Gal4>UAS-Chd1-RNAi*, n = 15, 12), subperineurial glia (*Spg-Gal4>UAS-Chd1-RNAi*, n = 11, 10), and perineurial glia (*NP6293-Gal4>UAS-Chd1-RNAi*, n = 12, 13). Mean ± SEM; *p < 0.05, **p < 0.01, ***p < 0.001, N.S. not significant; one-way ANOVA with Bonferroni test for multiple comparisons. Non-normalized raw data were used for statistical analysis.


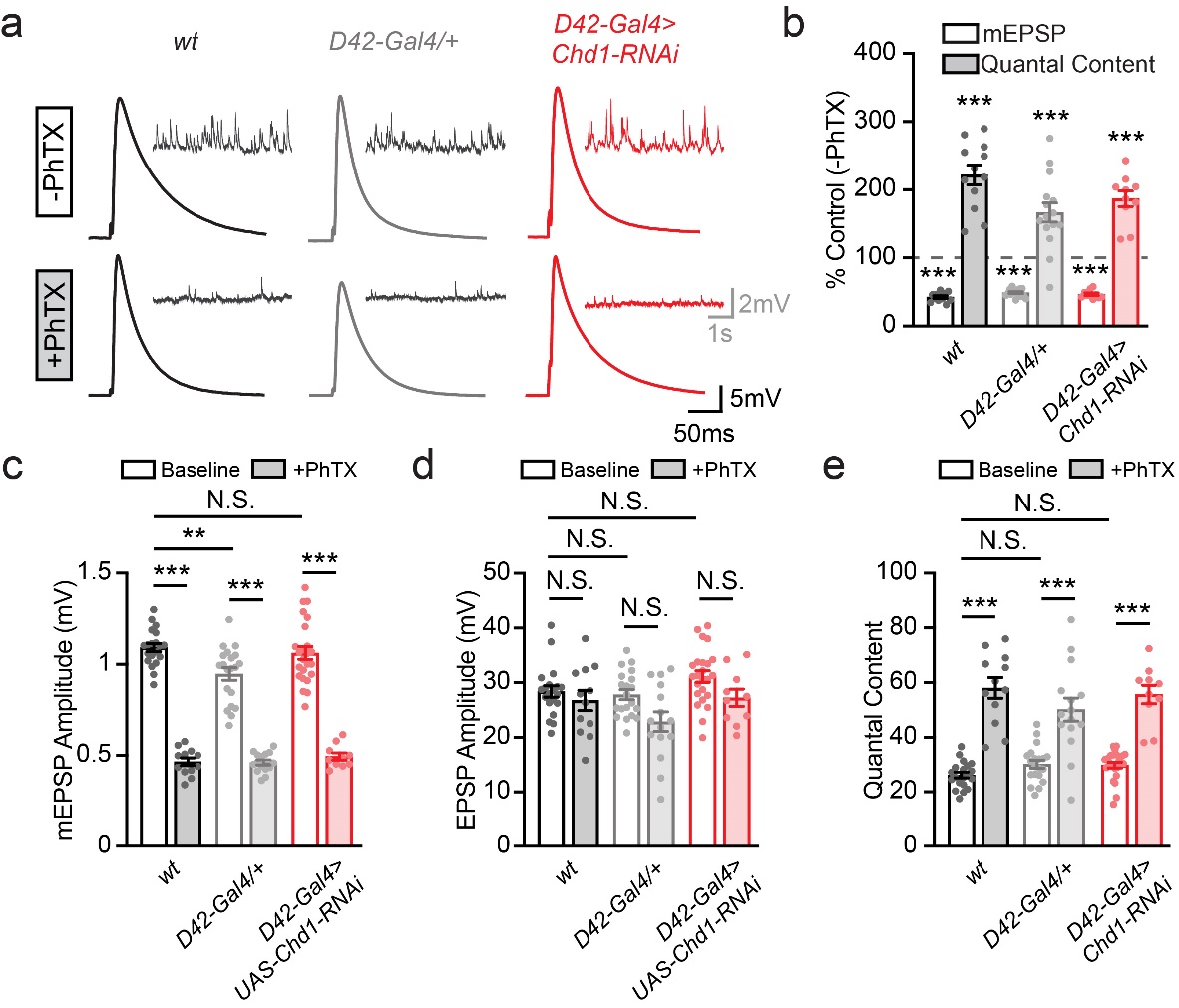


**Figure S7. Motoneuron-Specific *Chd1* Knockdown Does Not Affect Acute PHP.**

**a.** Representative mEPSP and EPSP traces from *wild-type* (*wt*), heterozygous *D42-Gal4* (*D42-Gal4/+*), and motoneuron knockdown of *Chd1 (D42-Gal4>UAS-Chd1-RNAi)* in the absence and presence (+PhTX) of philanthotoxin.

**b.** Average mEPSP amplitude (open bars) and presynaptic release (quantal content, filled bars), expressed as percent change with PhTX relative to baseline (-PhTX). Genotypes and sample sizes: *wild-type* (*wt*, n = 20, 12 for -PhTX and +PhTX, respectively), *D42-Gal4/+* (n = 20, 15), and motoneuron knockdown of *Chd1 (D42-Gal4>UAS-Chd1-RNAi,* n = 24, 10). Mean ± SEM; ***p < 0.001; one-way ANOVA with Bonferroni test for multiple comparisons. Non-normalized raw data were used for statistical analysis.

**c-e.** Non-normalized values corresponding to (**b**). Average mEPSP amplitude (**c**), EPSP amplitude (**d**), and quantal content (**e**) in the absence (open bars) and presence (filled bars) of PhTX. Mean ± SEM; **p < 0.01, ***p < 0.001, N.S. not significant; one-way ANOVA with Bonferroni test for multiple comparisons.

**
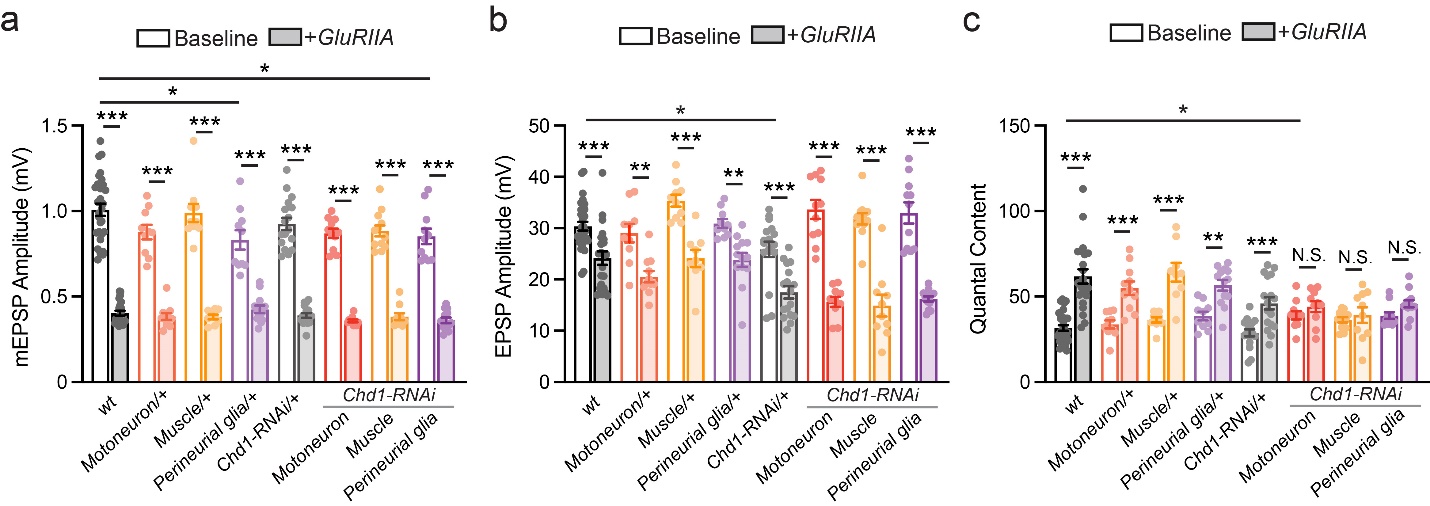
**

**Figure S8. Raw Data for Tissue-Specific *Chd1* Knockdown in Chronic PHP.**

**a-c.** Non-normalized raw data for average mEPSP amplitude (**a**), EPSP amplitude (**b**), and presynaptic release (quantal content, **c**), in the absence (baseline, open bars) and presence (+*GluRIIA*, filled bars) of the *GluRIIA* mutation. Genotypes and sample sizes: *wild-type* (*wt*, n = 30, 22 for -*GluRIIA* and +*GluRIIA*, respectively), motoneuron *Gal4* control (*OK371-Gal4/+*, n = 10, 11), muscle *Gal4* control (*MHC-Gal4/+*, n = 10, 9), perineurial glial *Gal4* control (*NP6293-Gal4/+*, n = 10, 13), *UAS-Chd1-RNAi* control (*Chd1-RNAi/+*, n = 17, 17), motoneuron-specific knockdown (*OK371-Gal4>UAS-Chd1-RNAi*, n = 11, 10), muscle-specific knockdown (*MHC-Gal4>UAS-Chd1-RNAi*, n = 12, 10), and perineurial glial-specific knockdown of *Chd1* (*NP6293-Gal4>UAS-Chd1-RNAi*, n = 11, 13). Mean ± SEM; *p<0.05, **p < 0.01, ***p < 0.001, N.S. not significant; one-way ANOVA with Bonferroni test for multiple comparisons. Non-normalized raw data were used for statistical analysis.

**
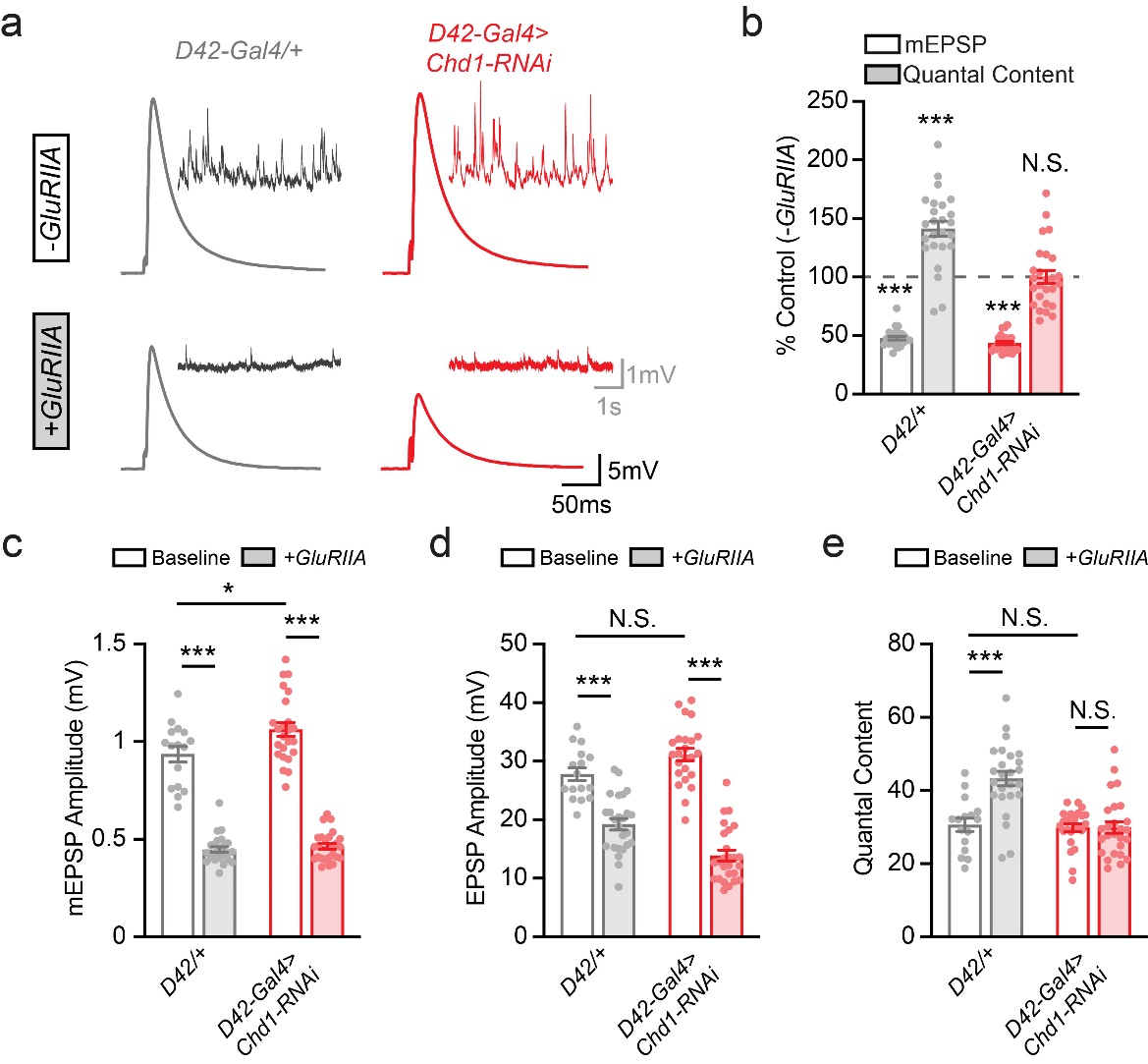
**

**Figure S9. Motoneuron-Specific *Chd1* Knockdown Disrupts Chronic PHP.**

**a.** Representative mEPSP and EPSP traces from heterozygous *D42-Gal4* (*D42-Gal4/+*), motoneuron-specific *Chd1* knockdown (*D42-Gal4>UAS-Chd1-RNAi*), *D42-Gal4/+,GluRIIA* mutants, and motoneuron-specific *Chd1* knockdown in *GluRIIA* mutant background *(D42-Gal4>UAS-Chd1-RNAi,GluRIIA).*

**b.** Average mEPSP amplitude (open bars) and presynaptic release (quantal content, filled bars), expressed as percent change in *GluRIIA* mutants compared to the same genotype without the *GluRIIA* mutation. Genotypes and sample sizes: *D42-Gal4/+* (n = 16), *D42-Gal4>UAS-Chd1-RNAi* (n = 24), *D42/+,GluRIIA* (n = 25), *D42-Gal4>UAS-Chd1-RNAi,GluRIIA* (n = 26). Mean ± SEM; ***p < 0.001, N.S. not significant; one-way ANOVA with Bonferroni test for multiple comparisons.

**c-e.** Non-normalized values corresponding to (**b**). Average mEPSP amplitude (**c**), EPSP amplitude (**d**), and quantal content (**e**) in the absence (open bars) and presence (filled bars) of the *GluRIIA* mutation. Mean ± SEM; *p < 0.05, ***p < 0.001, N.S. not significant; one-way ANOVA with Bonferroni test for multiple comparisons.

**
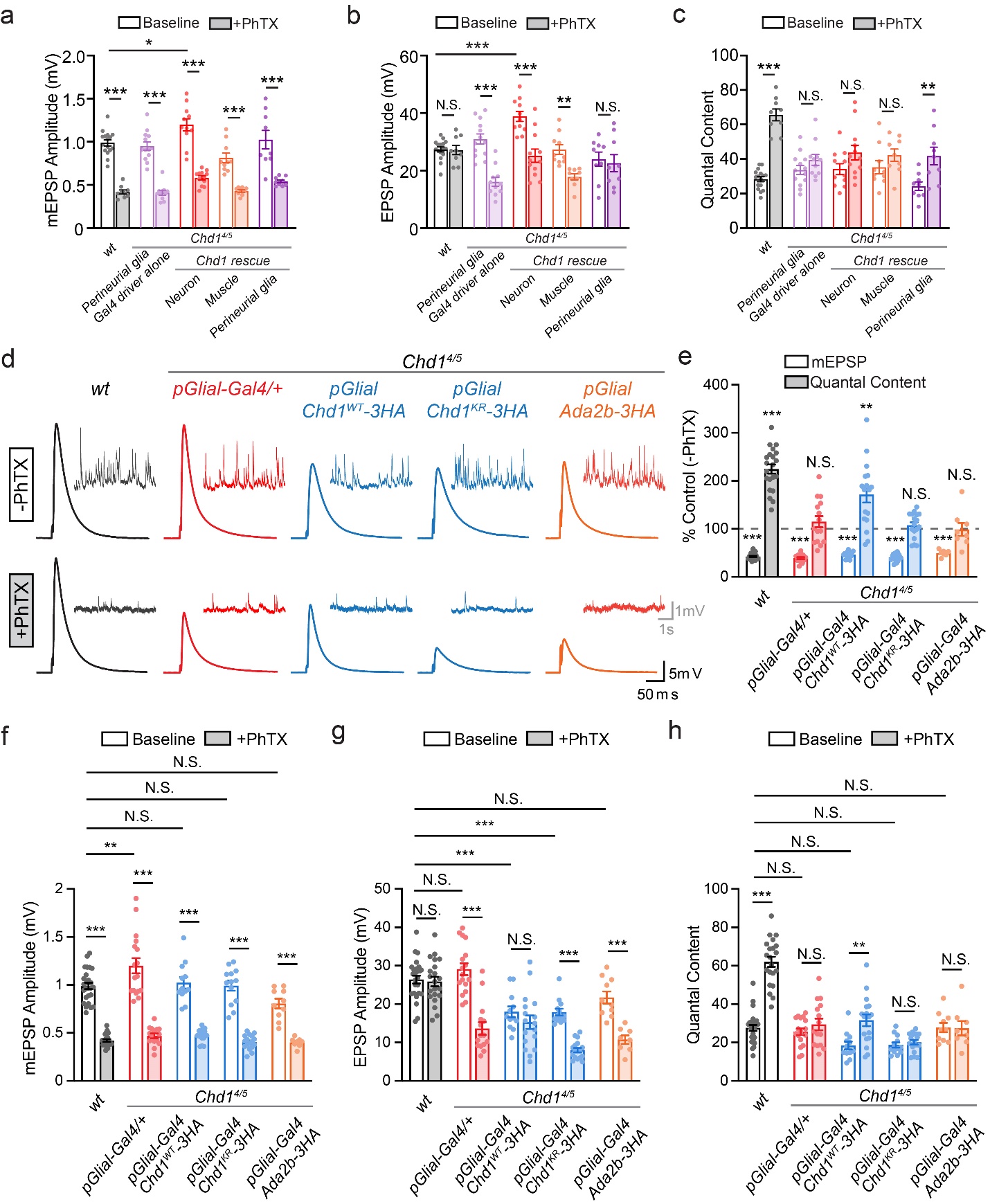
**

**Figure S10. Raw Data for Tissue-Specific Rescue of *Chd1* in Acute PHP and Glial Overexpression of *Chd1* with ATPase Mutation or *Ada2b* Does Not Rescue PHP.**

**a-c.** Non-normalized raw data for average mEPSP amplitude (**a**), EPSP amplitude (**b**), and presynaptic release (quantal content, **c**), in the absence (baseline, open bars) and presence (+PhTX, filled bars) of philanthotoxin. Genotypes and sample sizes: *wild-type* (*wt*, n = 16, 9 for -PhTX and +PhTX, respectively), perineurial glial *Gal4* control (*NP6293-Gal4/+;Chd1^4/5^*, n = 13, 11), pan-neuronal-specific rescue (*elav^C155^-Gal4>UAS-Chd1;Chd1^4/5^*, n = 11, 12), muscle-specific rescue (*MHC-Gal4>UAS-Chd1;Chd1^4/5^*, n = 10, 9), and perineurial glial-specific rescue (*NP6293-Gal4>UAS-Chd1;Chd1^4/5^*, n = 9, 9). Mean ± SEM; *p < 0.05, **p < 0.01, ***p < 0.001, N.S. not significant; one-way ANOVA with Bonferroni test for multiple comparisons. Non-normalized raw data were used for statistical analysis.

**d.** Representative mEPSP and EPSP traces from *wild-type* (*wt*), perineurial glial *Gal4* control (*NP6293-Gal4/+;Chd1^4/5^*), perineurial glial-specific rescue with *wild-type Chd1-3HA* (*NP6293-Gal4>UAS-Chd1-3HA;Chd1^4/5^*), ATPase mutated *Chd1-3HA* (*NP6293-Gal4>UAS-Chd1^KR^-3HA;Chd1^4/5^*), and *Ada2b-3HA* (*NP6293-Gal4>UAS-Ada2b-3HA;Chd1^4/5^*) in the *Chd1^4/5^* mutant background, in the absence (-PhTX) and presence (+PhTX) of philanthotoxin.

**e.** Normalized average mEPSP amplitude (open bars) and presynaptic release (quantal content, filled bars), expressed as the percent change in the presence of PhTX compared to the same genotype recorded in the absence of PhTX. Genotypes and sample sizes: *wild-type* (*wt*, n = 26, 21 for -PhTX and +PhTX, respectively), perineurial glial *Gal4* control (*NP6293-Gal4/+;Chd1^4/5^*, n = 17, 15), perineurial glial-specific rescue with *wild-type Chd1-3HA* (*NP6293-Gal4>UAS-Chd1-3HA;Chd1^4/5^*, n = 12, 17), ATPase mutated *Chd1-3HA* (*NP6293-Gal4>UAS-Chd1^KR^-3HA;Chd1^4/5^*, n = 13, 17), and *Ada2b-3HA* (*NP6293-Gal4>UAS-Ada2b-3HA;Chd1^4/5^*, n = 10, 8) in the *Chd1^4/5^* mutant background. Mean ± SEM; **p < 0.01, ***p < 0.001, N.S. not significant; one-way ANOVA with Bonferroni test for multiple comparisons. Non-normalized raw data were used for statistical analysis.

**f-h.** Non-normalized values corresponding to (**e**). Average mEPSP amplitude (**f**), EPSP amplitude (**g**), and quantal content (**h**) in the absence (open bars) and presence (filled bars) of the PhTX. Mean ± SEM; **p < 0.01, ***p < 0.001, N.S. not significant; one-way ANOVA with Bonferroni test for multiple comparisons.


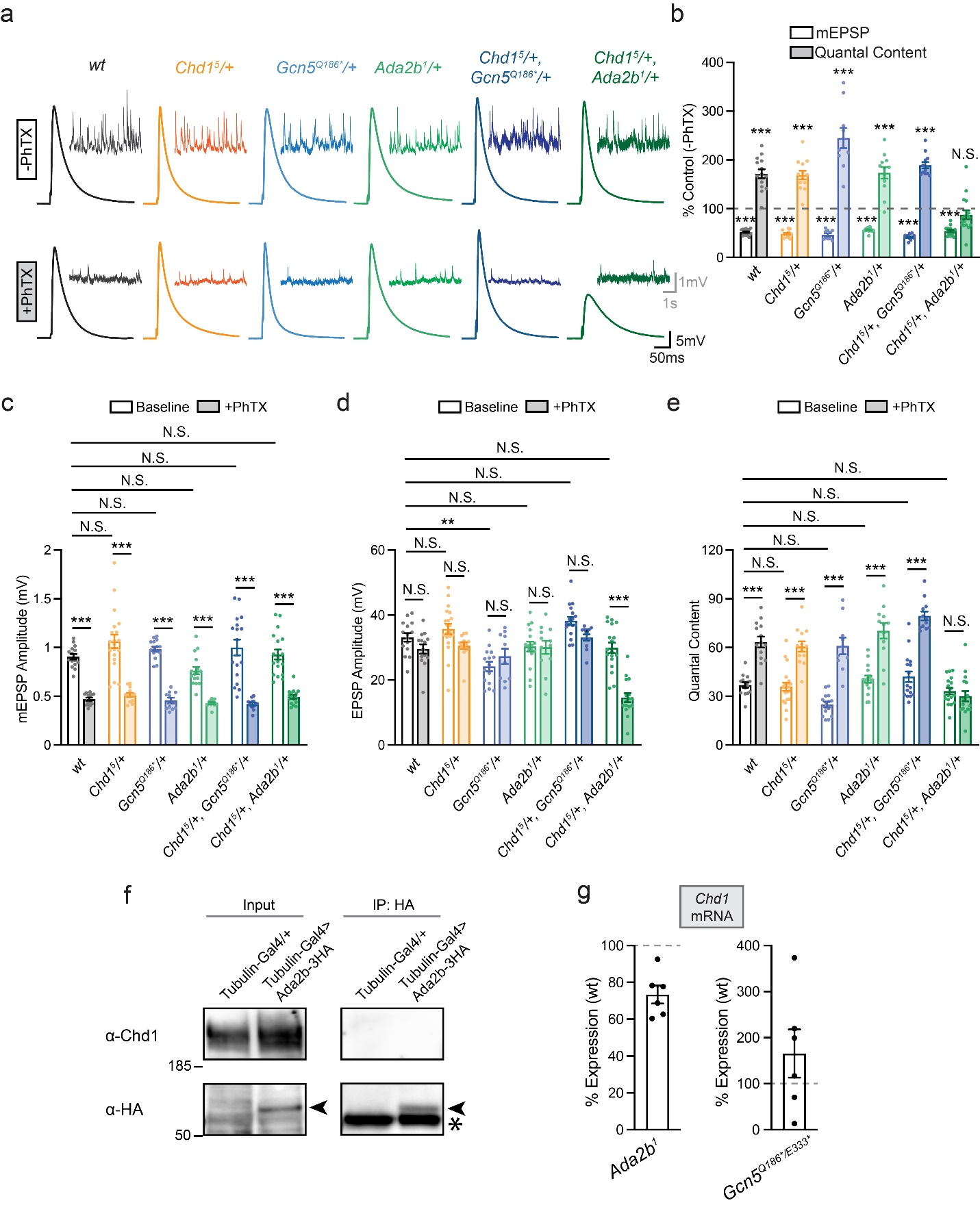


**Figure S11. *Chd1* Genetically Interact with *Ada2b* but Not with *Gcn5*.**

**a.** Representative mEPSP and EPSP traces from *wild-type* (*wt*), heterozygous *Chd1* (*Chd1^5^/+*), heterozygous *Gcn5* (*Gcn5^Q186*^/+*), heterozygous *Ada2b* (*Ada2b^1^/+*), trans-heterozygous *Chd1/+,Gcn5/+* (*Chd1^5^/+,Gcn5^Q186*^/+*), and trans-heterozygous *Chd1/+,Ada2b/+* (*Chd1^5^/+,Ada2b^1^/+*) mutants in the absence (-PhTX) and presence (+PhTX) of Philanthotoxin.

**b.** Average mEPSP amplitude (open bars) and quantal content (filled bars) for genotypes in (**a**), shown as the percent change in the presence of PhTX (+PhTX) relative to the absence (-PhTX). Sample sizes: *wild-type* (*wt*, n = 15, 14 for -PhTX and +PhTX, respectively), *Chd1^5^/+* (n = 20, 13), *Gcn5^Q186*^/+* (n = 15, 10), *Ada2b^1^/+* (n = 16, 12), *Chd1^5^/+,Gcn5^Q186*^/+* (n = 17, 12), and *Chd1^5^/+,Ada2b^1^/+* (n = 18, 16). Mean ± SEM; ***p < 0.001, N.S. not significant; one-way ANOVA with Bonferroni test for multiple comparisons. Non-normalized raw data were used for statistical analysis.

**c-e.** Non-normalized values corresponding to (**b**). Average mEPSP amplitude (**c**), EPSP amplitude (**d**), and quantal content (**e**) in the absence (open bars) and presence (filled bars) of the PhTX. Mean ± SEM; **p < 0.01, ***p < 0.001, N.S. not significant; one-way ANOVA with Bonferroni test for multiple comparisons.

**f.** Chd1 does not interact with Ada2b. HA-tagged Ada2b (*Tubulin-Gal4>UAS-Ada2b-3HA*) was immunoprecipitated (IP) using an HA antibody, and Chd1 was detected by immunoblotting (right panels). Input lysates are shown in the left panels. Ada2b-HA bands are indicated by arrowheads, and IgG heavy chains are indicated by an asterisk.

**g.** mRNA expression levels of *Chd1* in *wild-type*, *Ada2b^1^* homozygous, and *Gcn5^Q186*/E333*^* trans-allelic homozygous mutants (n = 6 per genotype) shown as fold change relative to the housekeeping gene *Rpl32*. mRNA expression was normalized to *wild-type*. Mean ± SEM.

**
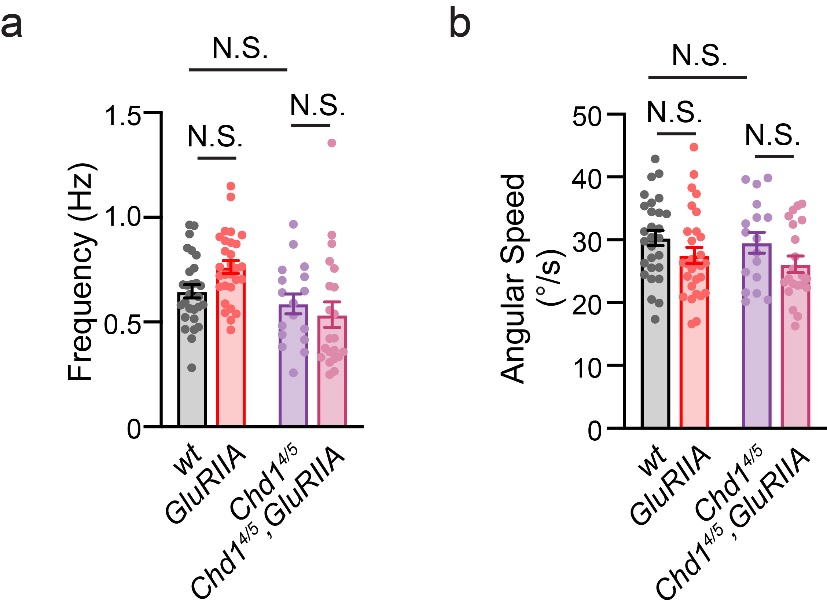
**

**Figure S12. Loss of *Chd1* Does Not Affect Crawling Stride Frequency or Angular Speed.**

**a-b.** Quantification of larval crawling stride frequency (**a**) and average angular speed (**b**) in *wild-type* (*wt*, n = 28), *GluRIIA* mutants (n = 29), *Chd1^4/5^* mutants (n = 17), and *Chd1^4/5^,GluRIIA* double mutants (n = 21). Mean ± SEM; N.S. not significant; one-way ANOVA with Bonferroni test for multiple comparisons.


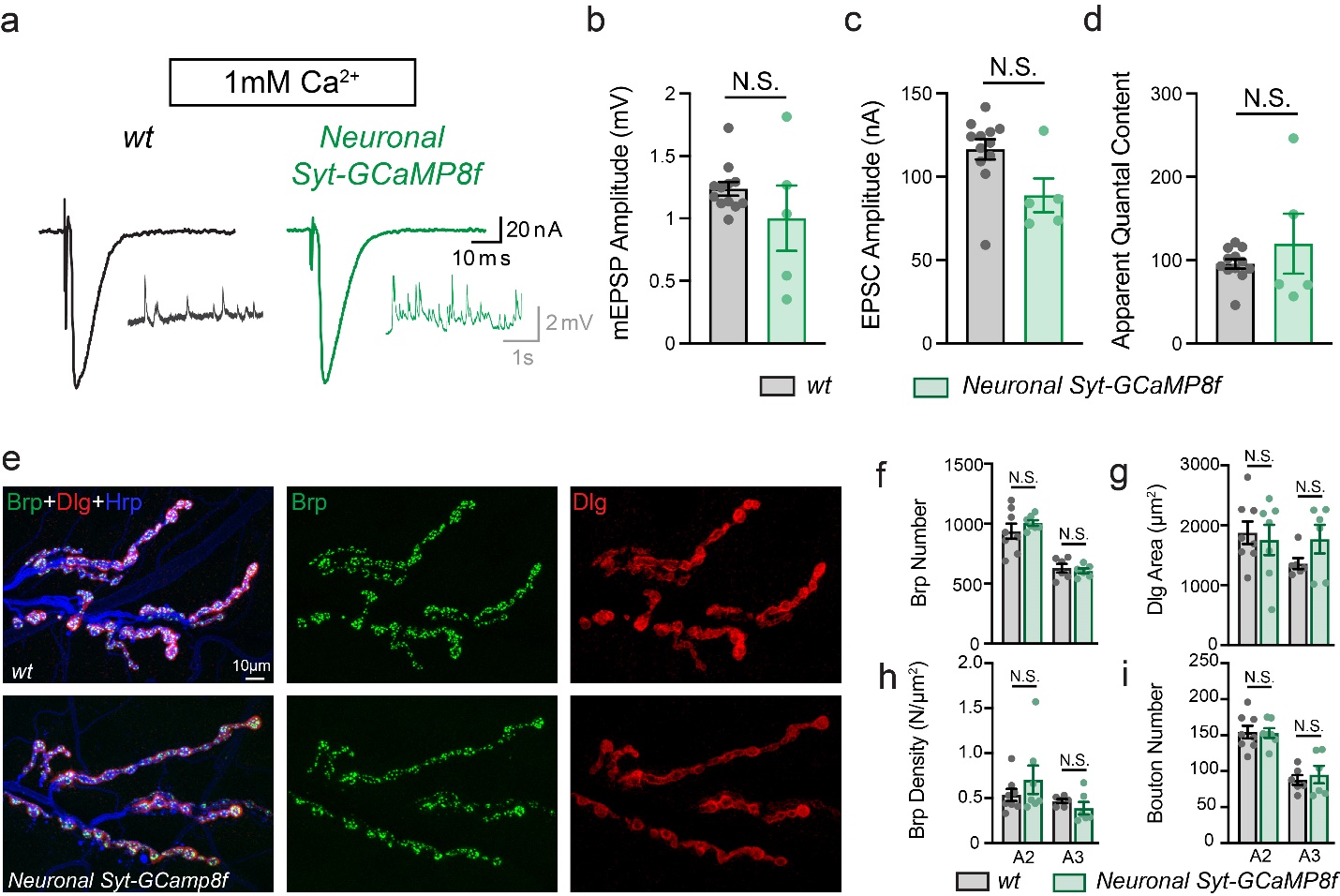


**Figure S13. Neuronal-Specific Expression of *UAS-Syt-GCaMP8f* Does Not Affect Synaptic Transmission at Baseline.**

**a.** Representative mEPSP and EPSC traces from *wild-type* (*wt*) and neuronal expression of *UAS-Syt-GCaMP8f* (*elav^C155^-Gal4>UAS-Syt-GCaMP8f*).

**b-d.** Average mEPSP amplitude (**b**), EPSC amplitude (**c**), and apparent quantal content (**d**) in *wild-type* (*wt*, n = 12) and neuronal expression of *UAS-Syt-GCaMP8f* (*Elav^C155^-Gal4>UAS-Syt-GCaMP8f*, n = 5). Mean ± SEM; N.S. not significant; Unpaired Student’s t-test comparing *wild-type* and neuronal expression groups.

**e.** Representative confocal images of the NMJ at muscle 6/7 (abdominal segment 2) in *wild-type* (*wt*) and neuronal expression of *UAS-Syt-GCaMP8f* (*elav^C155^-Gal4>UAS-Syt-GCaMP8f*). NMJs were immunolabeled with anti-Bruchpilot (Brp, green), anti-Discs large (Dlg, red), and neuronal membrane marker (HRP, blue).

**f-i.** Quantification of NMJ structure in *wild-type* (*wt*) and neuronal expression of *UAS-Syt-GCaMP8f* (*elav^C155^-Gal4>UAS-Syt-GCaMP8f*): total number of presynaptic Brp puncta (**f**), total postsynaptic Dlg area (**g**), Brp density (Brp puncta/Dlg area**, h**), and total number of synaptic boutons (**i**) at muscle 6/7 in abdominal segment 2 (A2) and segment 3 (A3). Sample sizes: *wild-type* (*wt*, n = 8, 6 synapses for A2 and A3, respectively) and *elav^C155^-Gal4>UAS-Syt-GCaMP8f* (n = 7, 6 synapses for A2 and A3, respectively). Mean ± SEM; N.S. not significant; unpaired Student’s t-test comparing *wild-type* and neuronal expression groups within the same muscle segment.

**
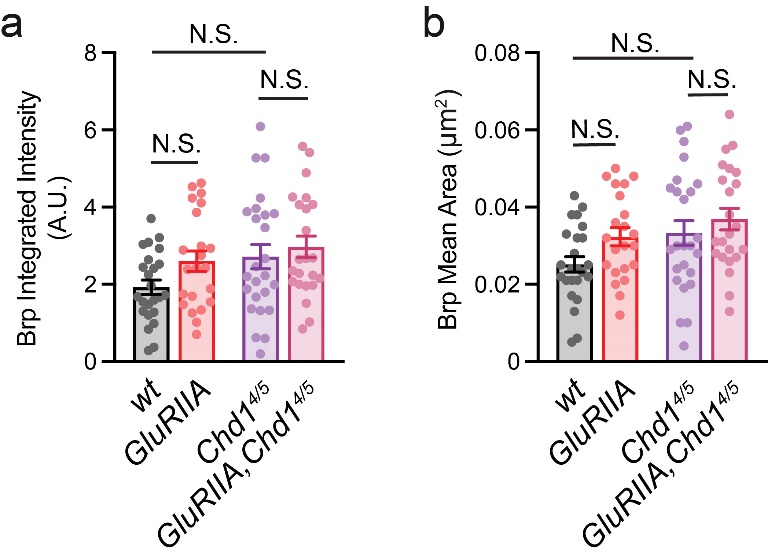
**

**Figure S14. *Chd1* Is Not Required for Regulating Brp Intensity and Area in Chronic PHP.**

**a-b.** Average Brp integrated intensity **(a)** and Brp area **(b)** in *wild-type* (*wt*, n = 24), *GluRIIA* mutants (n = 23), *Chd1^4/5^* mutants (n = 22), and *Chd1^4/5^,GluRIIA* double mutants (n = 26). Mean ± SEM; N.S. not significant; one-way ANOVA with Bonferroni test for multiple comparisons.

**
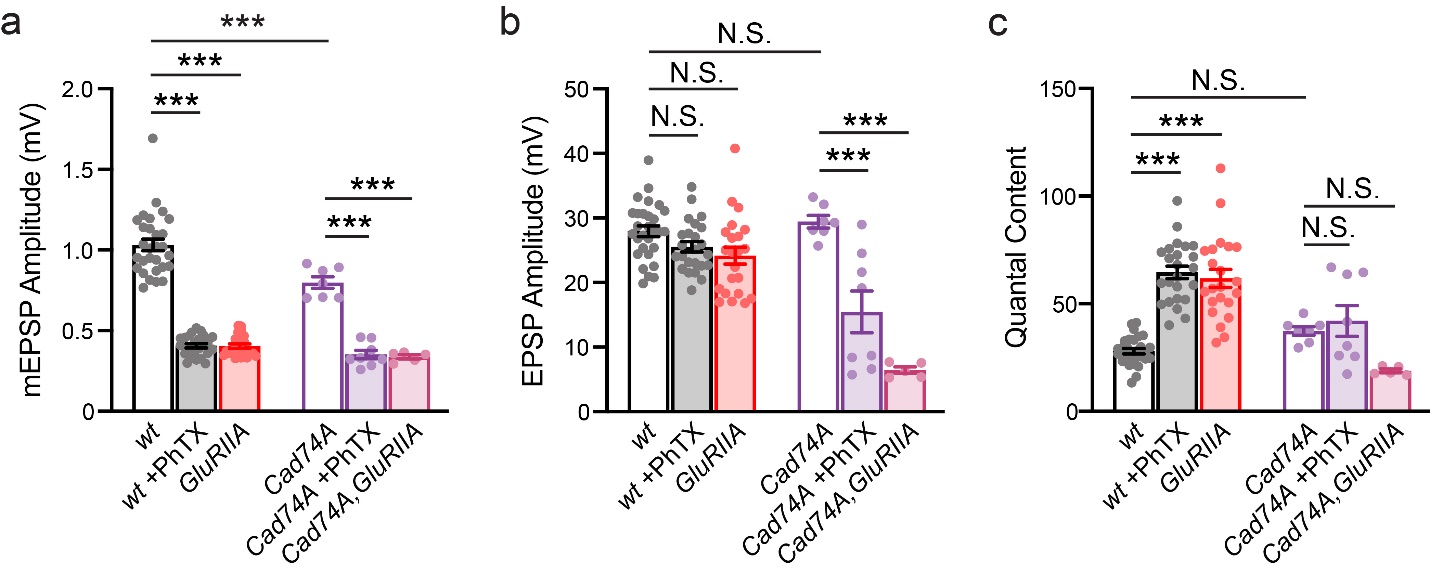
**

**Figure S15. Raw Data for *Cad74A* Mutants in PHP.**

**a-c.** Non-normalized raw data for average mEPSP amplitude (**a**), EPSP amplitude (**b**), and presynaptic release (quantal content, **c**) in the absence or presence of PhTX (+PhTX) or homozygous *GluRIIA* mutation (+*GluRIIA*). Genotypes and sample sizes: *wild-type* (*wt*, n = 29, 24 for –PhTX and +PhTX, respectively) and *Cad74A* (n = 7, 8 for –PhTX and +PhTX, respectively); *wild-type* (*wt*, n = 29), *GluRIIA* (n = 22), *Cad74A* (n = 7), and *Cad74A,GluRIIA* (n = 5). Mean ± SEM; ***p < 0.001, N.S. not significant; one-way ANOVA with Bonferroni test for multiple comparisons. Non-normalized raw data were used for statistical analysis.


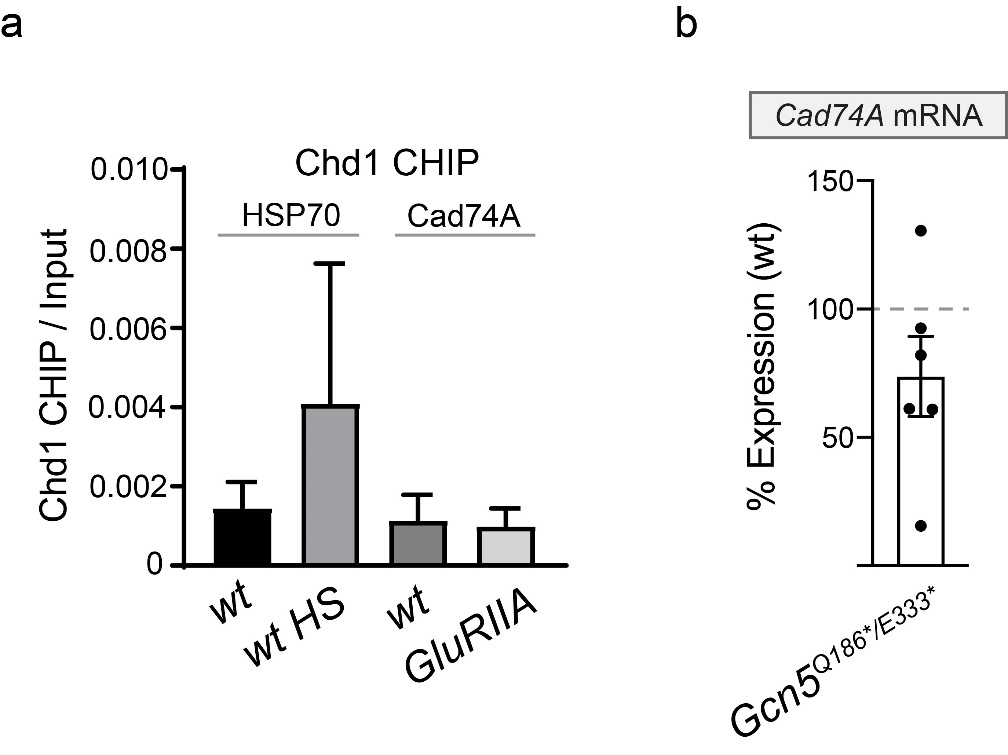


**Figure S16. *Chd1* Occupancy of *Cad74A* Promoter during Chronic PHP.**

**a.** IP/Input ratios from ChIP-PCR assays for Chd1 immunoprecipitation: *wild-type* (*wt*) vs. heat-shocked *wild-type* (*wt HS*) flies using *Hsp70* primers, and *wild-type* vs. *GluRIIA* mutants using *Cad74A* primers (n = 3 biological replicates).

**b.** mRNA expression levels of *Cad74A* in *wild-type* and *Gcn5^Q186*/E333*^* trans-allelic homozygous (n = 6 biological replicates) mutants shown as fold change relative to the housekeeping gene *Rpl32*. mRNA expression was normalized to *wild-type*. Mean ± SEM.


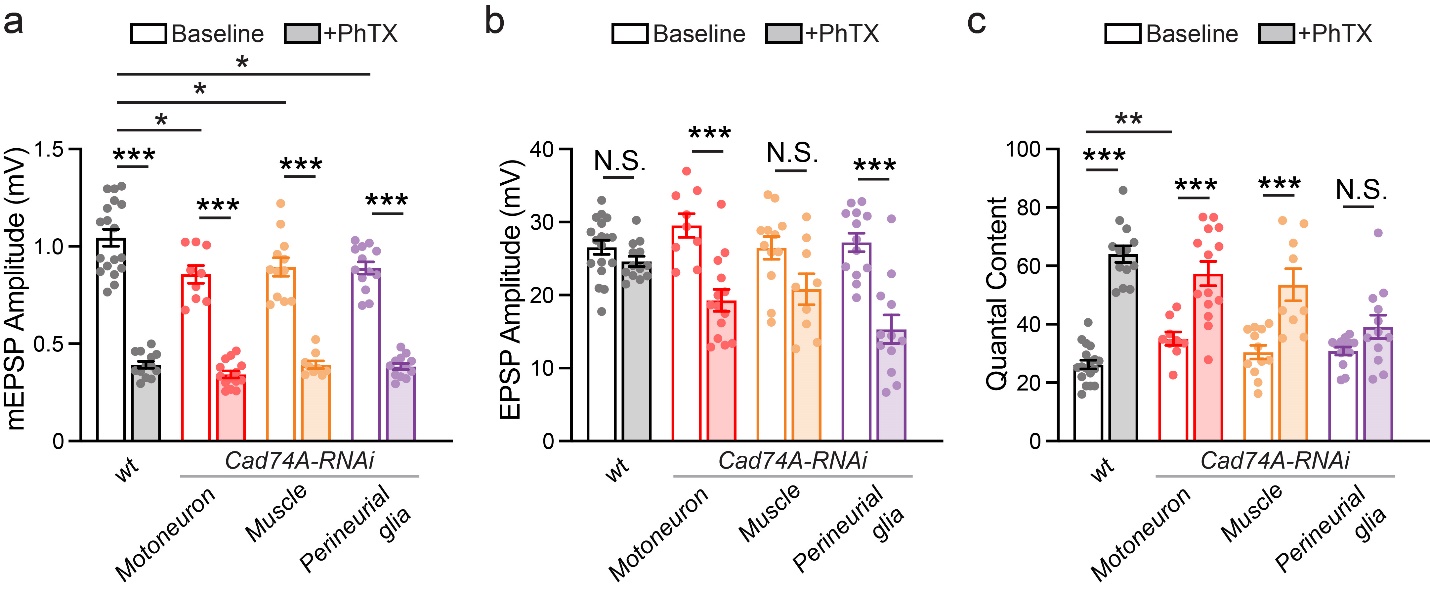


**Figure S17. Raw Data for Tissue-Specific Knockdown of *Cad74A* in Acute PHP.**

**a-c.** Non-normalized raw data for average mEPSP amplitude (**a**), EPSP amplitude (**b**) and presynaptic release (quantal content, **c**) in the absence (baseline, open bars) or presence (+PhTX, filled bars) of philanthotoxin. Genotypes and sample sizes: *wild-type* (*wt*, n = 18, 13 for -PhTX and +PhTX, respectively), knockdown of *Cad74A* in motoneurons (*OK371-Gal4>UAS-Cad74A-RNAi*, n = 9, 14), muscle (*MHC-Gal4>UAS-Cad74A-RNAi,* n = 12, 9), and perineurial glia (*NP6293-Gal4>UAS-Cad74A-RNAi*, n = 13, 12). Mean ± SEM; *p < 0.05, **p < 0.01, ***p < 0.001, N.S. not significant; one-way ANOVA with Bonferroni test for multiple comparisons. Non-normalized raw data were used for statistical analysis.


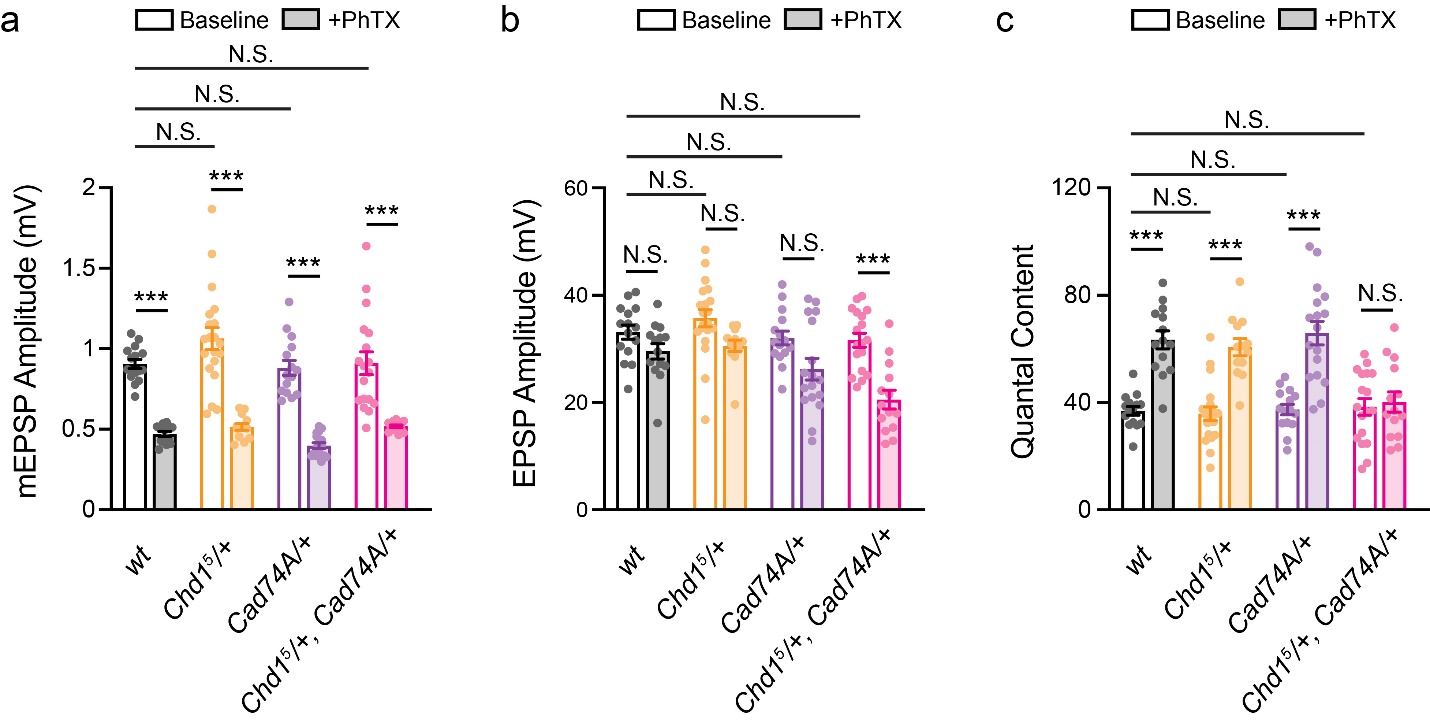


**Figure S18. Raw Data for *Chd1* and *Cad74A* Genetic Interaction in Acute PHP.**

**a-c.** Non-normalized raw data for average mEPSP amplitude (**a**), EPSP amplitude (**b**) and presynaptic release (quantal content, **c**) in the absence (baseline, open bars) or presence (+PhTX, filled bars) of philanthotoxin. Genotypes and sample sizes: *wild-type* (*wt*, n = 15, 14 for -PhTX and +PhTX, respectively), *Chd1^5^/+* (n = 20, 13), *Cad74A/+* (n = 15, 17), and *Chd1^5^/+,Cad74A/+* (n = 18, 14). Mean ± SEM; ***p < 0.001, N.S. not significant; one-way ANOVA with Bonferroni test for multiple comparisons. Non-normalized raw data were used for statistical analysis.

**
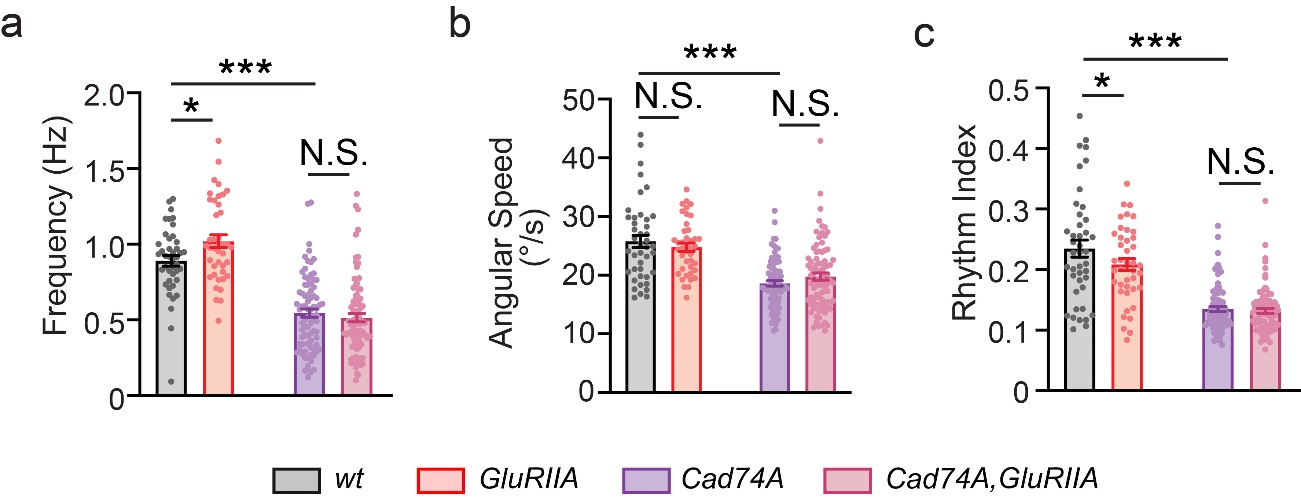
**

**Figure S19. Larval Crawling Behavior in *Cad74A* Mutants.**

**a-c.** Quantification of larval locomotion: stride frequency (**a**), angular speed (**b**), and rhythm index (**c**) in *wild-type* (*wt*, n = 42), *GluRIIA* mutants (n = 41), *Cad74A* mutants (n = 80), and *Cad74A,GluRIIA* double mutants (n = 84). Mean ± SEM; *p < 0.05, ***p < 0.001, N.S. not significant; one-way ANOVA with Bonferroni test for multiple comparisons.

**Table S1.**

| **is_PhTX** | **mutant** | **fly_gene** | **mEPSP (mV)** | **EPSP (mV)** | **QC** | **Vm (mV)** | **n** | **pvalue** |
| --- | --- | --- | --- | --- | --- | --- | --- | --- |
| FALSE | NP>BL44499 | Muc68Ca RNAi | 0.63 | 24.22 | 40.35 | -65.08 | 6 | 0.9679 |
| TRUE | NP>BL44499 | Muc68Ca RNAi | 0.34 | 13.28 | 40.67 | -64.67 | 7 | 0.9679 |
| FALSE | BL85038 | Tie | 0.99 | 22.04 | 22.32 | -68.66 | 6 | 0.9069 |
| TRUE | BL85038 | Tie | 0.43 | 9.74 | 22.84 | -60.62 | 6 | 0.9069 |
| FALSE | BL26109 | kon | 0.76 | 23.15 | 30.96 | -62.65 | 6 | 0.8628 |
| TRUE | BL26109 | kon | 0.37 | 10.67 | 29.92 | -59.33 | 9 | 0.8628 |
| FALSE | BL29204 | kek4 | 0.94 | 23.89 | 25.40 | -62.59 | 5 | 0.7371 |
| TRUE | BL29204 | kek4 | 0.44 | 12.34 | 27.74 | -61.40 | 8 | 0.7371 |
| FALSE | BL37623 | dlp | 0.84 | 19.79 | 23.99 | -62.40 | 8 | 0.6919 |
| TRUE | BL37623 | dlp | 0.41 | 10.39 | 25.53 | -59.51 | 6 | 0.6919 |
| FALSE | NP>V51330 | CG31342 RNAi | 0.91 | 27.69 | 31.22 | -68.06 | 6 | 0.6065 |
| TRUE | NP>V51330 | CG31342 RNAi | 0.37 | 12.45 | 35.16 | -68.29 | 9 | 0.6065 |
| FALSE | BL18312 | Cad74A | 0.80 | 29.42 | 37.30 | -69.53 | 7 | 0.5625 |
| TRUE | BL18312 | Cad74A | 0.35 | 15.46 | 42.01 | -66.68 | 8 | 0.5625 |
| FALSE | BL61737 | CG1674 | 1.16 | 26.69 | 23.23 | -63.99 | 5 | 0.5573 |
| TRUE | BL61737 | CG1674 | 0.44 | 8.72 | 19.34 | -61.90 | 6 | 0.5573 |
| FALSE | BL18540 | Cht11 | 0.70 | 16.42 | 24.49 | -66.49 | 15 | 0.5393 |
| TRUE | BL18540 | Cht11 | 0.31 | 8.51 | 27.55 | -69.34 | 20 | 0.5393 |
| FALSE | BL22389 | Manf | 0.79 | 19.63 | 26.51 | -62.47 | 8 | 0.5120 |
| TRUE | BL22389 | Manf | 0.36 | 8.25 | 21.81 | -67.31 | 5 | 0.5120 |
| FALSE | BL21398 | Cirl | 0.82 | 28.49 | 36.45 | -65.75 | 9 | 0.3183 |
| TRUE | BL21398 | Cirl | 0.37 | 16.43 | 42.65 | -63.37 | 10 | 0.3183 |
| FALSE | BL34441 | sdk | 1.19 | 27.52 | 23.51 | -64.93 | 10 | 0.3164 |
| TRUE | BL34441 | sdk | 0.46 | 12.96 | 28.33 | -65.94 | 15 | 0.3164 |
| FALSE | NP>V16133 | Atg1 RNAi | 1.09 | 34.08 | 34.08 | -77.19 | 6 | 0.2209 |
| TRUE | NP>V16133 | Atg1 RNAi | 0.33 | 13.49 | 42.37 | -67.18 | 8 | 0.2209 |
| FALSE | BL11007 | trol | 1.10 | 22.86 | 21.04 | -64.68 | 11 | 0.1759 |
| TRUE | BL11007 | trol | 0.43 | 11.42 | 27.40 | -65.80 | 17 | 0.1759 |
| FALSE | BL14900 | crq | 0.81 | 20.14 | 25.21 | -61.92 | 12 | 0.0369 |
| TRUE | BL14900 | crq | 0.40 | 14.76 | 36.31 | -64.73 | 14 | 0.0369 |
| FALSE | NP>V853 | babo RNAi | 0.91 | 23.73 | 27.41 | -66.02 | 9 | 0.0369 |
| TRUE | NP>V853 | babo RNAi | 0.40 | 15.17 | 38.35 | -64.69 | 13 | 0.0369 |
| FALSE | BL23336 | Syn1 | 0.73 | 18.27 | 25.16 | -59.57 | 7 | 0.0252 |
| TRUE | BL23336 | Syn1 | 0.37 | 14.60 | 40.04 | -60.28 | 6 | 0.0252 |
| FALSE | BL27807 | 5-HT1A | 0.98 | 16.27 | 16.64 | -60.57 | 8 | 0.0150 |
| TRUE | BL27807 | 5-HT1A | 0.42 | 14.74 | 34.89 | -61.81 | 11 | 0.0150 |
| FALSE | BL8486 | Ptp69D | 1.29 | 22.18 | 18.11 | -57.29 | 5 | 0.0108 |
| TRUE | BL8486 | Ptp69D | 0.38 | 13.94 | 39.13 | -59.17 | 8 | 0.0108 |
| FALSE | NP>V24104 | HtrA2 RNAi | 0.98 | 25.24 | 26.95 | -64.85 | 6 | 0.0100 |
| TRUE | NP>V24104 | HtrA2 RNAi | 0.36 | 15.32 | 43.63 | -62.54 | 6 | 0.0100 |
| FALSE | BL12425 | Rtnl1 | 0.91 | 22.36 | 26.91 | -65.79 | 11 | 0.0096 |
| TRUE | BL12425 | Rtnl1 | 0.36 | 15.41 | 44.64 | -65.36 | 15 | 0.0096 |
| FALSE | BL37815 | Ten-a | 1.33 | 25.59 | 20.55 | -59.31 | 7 | 0.0088 |
| TRUE | BL37815 | Ten-a | 0.43 | 19.57 | 46.89 | -64.56 | 7 | 0.0088 |
| FALSE | BL30711/BL56111 | SPARC | 0.98 | 21.94 | 23.14 | -63.62 | 9 | 0.0085 |
| TRUE | BL30711/BL56111 | SPARC | 0.41 | 14.70 | 36.71 | -65.63 | 12 | 0.0085 |
| FALSE | BL35950 | neur | 1.20 | 25.72 | 21.18 | -65.31 | 9 | 0.0070 |
| TRUE | BL35950 | neur | 0.41 | 14.73 | 36.45 | -65.71 | 11 | 0.0070 |
| FALSE | BL18141 | Tsp3A | 1.07 | 21.98 | 21.02 | -73.24 | 7 | 0.0022 |
| TRUE | BL18141 | Tsp3A | 0.36 | 16.70 | 46.79 | -68.01 | 11 | 0.0022 |
| FALSE | BL26103 | CadN2 | 0.89 | 24.55 | 28.44 | -60.14 | 9 | 0.0007 |
| TRUE | BL26103 | CadN2 | 0.41 | 18.13 | 44.16 | -64.61 | 12 | 0.0007 |
| FALSE | BL8594 | trio | 0.85 | 34.96 | 41.51 | -58.68 | 6 | 0.0001 |
| TRUE | BL8594 | trio | 0.40 | 33.54 | 83.84 | -67.21 | 8 | 0.0001 |
| FALSE | NP>V13566 | Lgr1 RNAi | 0.99 | 30.49 | 31.33 | -63.49 | 8 | 0.0000 |
| TRUE | NP>V13566 | Lgr1 RNAi | 0.37 | 31.54 | 86.72 | -61.14 | 7 | 0.0000 |
| FALSE | WT | WT | 1.02 | 27.56 | 28.12 | -61.36 | 45 | 0.0000 |
| TRUE | WT | WT | 0.41 | 25.17 | 62.49 | -60.51 | 34 | 0.0000 |
| TRUE | BL26035 | Gfrl | 0.28 | 17.02 | 59.42 | -59.39 | 5 |  |
| TRUE | NP>V34743 | CG30280 RNAi | 0.42 | 25.21 | 62.15 | -67.02 | 10 |  |
| TRUE | NP>V51331 | CG31342 RNAi | 0.40 | 20.92 | 53.98 | -61.32 | 7 |  |
| TRUE | NP>BL28515 | Tsp86D RNAi | 0.45 | 20.19 | 45.53 | -62.74 | 7 |  |
| TRUE | BL19942 | HDAC4 | 0.48 | 22.48 | 46.84 | -63.18 | 4 |  |
| TRUE | BL34183 | CG33298 | 0.46 | 22.31 | 48.44 | -60.75 | 6 |  |
| TRUE | NP>BL65919 | Ptp36E RNAi | 0.42 | 20.77 | 49.83 | -67.77 | 3 |  |
| TRUE | BL22630 | mthl15 | 0.40 | 20.75 | 51.04 | -67.16 | 4 |  |
| TRUE | BL13625 | Asator | 0.41 | 21.38 | 52.04 | -58.17 | 6 |  |
| TRUE | NP>BL30483 | PlexA | 0.43 | 22.53 | 52.85 | -66.01 | 6 |  |
| TRUE | BL56620 | Drak | 0.42 | 22.58 | 53.58 | -66.40 | 6 |  |
| TRUE | NP>V29024 | CycD RNAi | 0.39 | 20.18 | 53.59 | -60.82 | 5 |  |
| TRUE | BL15625 | lama | 0.43 | 22.93 | 53.82 | -60.78 | 4 |  |
| TRUE | BL81638 | p120ctn | 0.46 | 24.01 | 54.72 | -60.55 | 3 |  |
| TRUE | NP>V38077 | Wnt2 RNAi | 0.40 | 22.50 | 56.36 | -59.78 | 5 |  |
| TRUE | BL16510 | Dscam1 | 0.36 | 20.27 | 56.75 | -65.13 | 4 |  |
| TRUE | BL32830 | mGluR | 0.45 | 25.29 | 57.58 | -59.58 | 6 |  |
| TRUE | BL20251 | Shrm | 0.45 | 26.23 | 58.86 | -59.71 | 5 |  |
| TRUE | BL60774 | Nuak | 0.39 | 22.31 | 59.29 | -71.35 | 4 |  |
| TRUE | BL34428 | mmd | 0.46 | 25.02 | 60.01 | -66.70 | 4 |  |
| TRUE | BL53129 | drpr | 0.42 | 25.30 | 60.37 | -61.43 | 5 |  |
| TRUE | BL53233 | Octalpha2R | 0.37 | 22.16 | 60.41 | -61.80 | 6 |  |
| TRUE | BL44178 | Trim9 | 0.43 | 26.36 | 60.58 | -63.64 | 5 |  |
| TRUE | BL30981 | CadN | 0.39 | 22.56 | 60.65 | -59.05 | 4 |  |
| TRUE | BL60867 | Actbeta | 0.36 | 21.14 | 60.83 | -58.30 | 5 |  |
| TRUE | NP>BL40938 | Lar RNAi | 0.33 | 20.05 | 61.89 | -59.29 | 4 |  |
| TRUE | BL13540 | Trim9 | 0.35 | 22.25 | 62.45 | -61.41 | 4 |  |
| TRUE | BL12331 | vlc | 0.34 | 20.87 | 62.80 | -61.53 | 6 |  |
| TRUE | BL41436 | shg | 0.48 | 30.80 | 63.72 | -68.36 | 2 |  |
| TRUE | BL42049 | magu | 0.42 | 26.68 | 66.14 | -60.38 | 5 |  |
| TRUE | BL30071 | Kul | 0.41 | 26.13 | 66.77 | -64.05 | 5 |  |
| TRUE | BL86012 | Cad96Cb | 0.38 | 25.02 | 66.98 | -68.65 | 4 |  |
| TRUE | NP>V6696 | kirre RNAi | 0.35 | 23.28 | 68.07 | -66.52 | 4 |  |
| TRUE | NP>V107205 | Creld RNAi | 0.39 | 26.13 | 68.23 | -62.46 | 5 |  |
| TRUE | BL61036 | Ndg | 0.40 | 27.94 | 69.13 | -64.52 | 7 |  |
| TRUE | BL19688 | Ten-m | 0.46 | 31.96 | 69.37 | -63.96 | 4 |  |
| TRUE | BL6958 | Wnt2 | 0.40 | 27.50 | 70.01 | -62.91 | 3 |  |

**Summary of Mutants and Electrophysiological Data from the Genetic Screen.**

Recording conditions with PhTX present (is PhTX: TRUE) or absent (is PhTX: FALSE) are indicated. For each genotype, *Drosophila* gene name, mEPSP amplitude, EPSP amplitude, quantal content (QC), resting membrane potential, and sample size are reported. Student’s t-test was used to calculate p values comparing QC measured in the presence versus absence of PhTX within the same mutant. WT refers to *wild-type*; NP refers to the *NP-6293-Gal4* (perineurial glia-specific driver). BDRC (BL) and VDRC (V) allele numbers are shown.

**References**

1. Berson A, Sartoris A, Nativio R, Van Deerlin V, Toledo JB, Porta S, Liu S, Chung CY, Garcia BA, Lee VM *et al*: **TDP-43 Promotes Neurodegeneration by Impairing Chromatin Remodeling**. *Curr Biol* 2017, **27**(23):3579-3590 e3576.

2. Zeisel A, Munoz-Manchado AB, Codeluppi S, Lonnerberg P, La Manno G, Jureus A, Marques S, Munguba H, He L, Betsholtz C *et al*: **Brain structure. Cell types in the mouse cortex and hippocampus revealed by single-cell RNA-seq**. *Science* 2015, **347**(6226):1138-1142.

3. Davie K, Janssens J, Koldere D, De Waegeneer M, Pech U, Kreft L, Aibar S, Makhzami S, Christiaens V, Bravo Gonzalez-Blas C *et al*: **A Single-Cell Transcriptome Atlas of the Aging Drosophila Brain**. *Cell* 2018, **174**(4):982-998 e920.
